# Gemmotherapy Extracts Like the Dog Rose, Lingonberry, Sea Buckthorn, Blackthorn, Common Grape, Hawthorn, Raspberry and Boxwood Feature Variable Yet Excelling Antimicrobial Effects

**DOI:** 10.3390/antibiotics14101052

**Published:** 2025-10-21

**Authors:** Melinda Héjja, Éva György, Ferenc Ádám Lóga, Róbert Nagy, Tünde Pacza, Péter Sipos, György Tankó, Éva Laslo, Noémi Mészáros, Violeta Turcuș, Neli-Kinga Oláh, Endre Máthé

**Affiliations:** 1 Doctoral School of Nutrition and Food Science, Faculty of Agricultural and Food Sciences and Environmental Management, University of Debrecen, Böszörményi Str. 128, H-4032 Debrecen, Hungary; 2 Institute of Nutrition Science, Faculty of Agricultural and Food Sciences and Environmental Management, University of Debrecen, Böszörményi Str. 128, H-4032 Debrecen, Hungary; berettyo.homoktovis@gmail.com (F.Á.L.); nagy.robert@agr.unideb.hu (R.N.); pacza.tunde@unideb.hu (T.P.); siposp@agr.unideb.hu (P.S.); gyorgy_tanko@yahoo.com (G.T.); 3 Department of Food Science, Faculty of Economics, Socio-Human Sciences and Engineering, Sapientia Hungarian University of Transylvania, Libertății Sq. 1., RO-530104 Miercurea Ciuc, Romania; gyorgyeva@uni.sapientia.ro (É.G.); lasloeva@uni.sapientia.ro (É.L.); 4 Department of Life Sciences, Faculty of Medicine, Vasile Goldis Western University of Arad, L. Rebreanu Str. 86, RO-310414 Arad, Romania; meszaros.noemi@uvvg.ro (N.M.); turcus.violeta@uvvg.ro (V.T.); 5 CE-MONT Mountain Economy Center, Costin C. Kirițescu National Institute of Economic Research, Romanian Academy, Petreni Str. 49, RO-725700 Suceava, Romania; 6 Department of Pharmaceutical Chemistry, Faculty of Pharmacy, Vasile Goldis, Western University of Arad, L. Rebreanu Str. 86, RO-310414 Arad, Romania; olah.neli@uvvg.ro; 7 PlantExtrakt Ltd., No. 46, RO-407059 Rădaia, Romania

**Keywords:** gemmotherapy extracts, antimicrobial activity, *Rosa canina*, Vaccinium vitis-idaea, *Hippophae rhamnoides*, *Prunus spinosa*, *Vitis vinifera*, *Crataegus oxyacantha*, *Rubus idaeus*, *Buxus sempervirens*

## Abstract

**Background:** Antibiotic resistance is spreading, and the effectiveness of the most widely used antibiotics is decreasing. These issues are global health and food safety concerns that require immediate attention. One potential solution is the use of various gemmotherapy extracts (GTEs). However, there is a paucity of studies investigating the presumptive antimicrobial activity of GTEs. **Methods**: In this comparative study, we are assessing the antimicrobial properties of eight selected GTEs, as well as their polyphenol content and antioxidant activity, against a panel of microorganisms (Gram-positive and Gram-negative bacteria, yeasts, and molds). We are using the agar diffusion method (ADM) and the broth microdilution method (BMD) to determine the minimum inhibitory concentration (MIC) and the minimum bactericidal concentration (MBC). **Results**: Among the analyzed extracts, dog rose, lingonberry, sea buckthorn, blackthorn, and common grape GTEs showed the highest total phenolic content, antioxidant activity, and the most relevant antimicrobial activity including certain differences with respect to the microbiostatic and/or microbicidal properties. These results demonstrate the relative strength of the antimicrobial effects of specific GTEs against certain microbial species, which could facilitate the use of these GTEs in personalized and/or specific antimicrobial therapies.

## 1. Introduction

Many studies have been conducted to prove that some plants are alternatives to antibiotics because they have antimicrobial effects against several microorganisms. These benefits are attributed to phytonutrients such as flavonoids, phenolics, coumarins, alkaloids, tannins, lectins, terpenoids, essential oils, polypeptides, and polyacetylenes [1].

Most studies on plant-based antimicrobials focus on extracts from differentiated tissues, such as fruits, leaves, bark, and seeds. Interestingly, few address extracts from meristematic tissues, such as buds, young shoots, and root tips, despite their superior bioactive composition. These young shoots contain several nutrients, and metabolites necessary for their optimal development, many of them presenting strong anti-inflammatory and anti-microbial properties. Due to the abundance of these compounds, these bud extracts were suggested to be more effective than extracts from differentiated tissues [2]. This type of phytotherapy was called gemmotherapy and the corresponding plant derived products were named gemmotherapy extracts (GTEs) [3,4].

In a previous study, we investigated the antimicrobial activity of seven different GTEs to assess their effect on microbial species relevant to the food safety and the human microbiome [5]. Among these, olive (*Olea europaea* L.) GTE was the most effective against all tested bacterial strains, even at relatively low concentrations, while the blackcurrant (*Ribes nigrum* L.) GTE had no bactericidal activity, but it did have bacteriostatic activity. Therefore, the blackcurrant GTE is expected to have less impact on the gut-specific microbiome. The higher antioxidant capacity of the blackcurrant bud compared to fruit extract suggests that the GTE is more suitable for inducing anti-neuroinflammatory and cardiovascular effects, although the underlying mechanisms pleads for further studies [6,7]. The antimicrobial effect of GTEs was also supported by Okińczyc et al. (2024) showing that poplar (*Populus* spp.) bud extracts were effective against several Gram-positive bacterial strains and *Helicobacter pylori*, while against *Candida* species only moderate effects were detected [8].

In the current study, we analyzed the antioxidant and antimicrobial activities of eight additional GTEs (blackthorn, sea buckthorn, raspberry, dog rose, lingonberry, hawthorn, common grape, and boxwood), and the relevant features of these plant species are presented below.

The blackthorn (*Prunus spinosa* L.—Psp) belongs to the *Rosaceae* family. It is a thorny shrubby plant with bluish-black fruits and it is widespread in Asia minor, northwest Africa and Europe, except the northeast and extreme north parts [9,10]. Studies have reported that in folk medicine, its fruits, flowers, bark, and root are used [11]. The fruits could treat colds, respiratory diseases, urinary tract diseases, diabetes and could be used externally for inflammation of the oral cavity [12,13]. In addition, it has antidiarrheal, astringent, and diuretic effects, and it is recommended for dysentery, stomach pain and cardiovascular diseases [14]. Marchelak et al. (2017) reported, that the flower extract protected human plasma components against peroxynitrite-induced damage and increased the overall antioxidant status of plasma [15].

Sea buckthorn (*Hippophae rhamnoides* L.—Hrh) belongs to the *Elaeagnaceae* family. It is a hardy, deciduous shrub with orange berries and it is native to Central Asia and north-western Europe, but it is also widespread in the USA and Canada [9,16,17]. In folk medicine, the leaves, berries, and oil have been used for the treatment of different pathological conditions and to prevent diseases. In traditional Chinese medicine, the plant has been used to treat asthma, gastric ulcers, skin diseases, and lung disorders. In Tibetan and Mongolian traditional medicines, the berries have been used to treat coughs and improve the function and the blood circulation of the digestive system [18]. They have also shown positive results in the treatment of damaged mucous membranes of the gastrointestinal tract and they have anti-inflammatory, anti-atherogenic, hypotensive, and hypocholesterolemic effects [19]. The berries contain high level of carotene, potassium, vitamin A, B_2_, C, and E, fatty acids (palmitic acid, oleic acid (omega-9), palmitoleic acid (omega-7), linoleic acid (omega-6), and linolenic acid (omega-3)), other organic acids, and amino acids [18,20].

Raspberry (*Rubus idaeus* L.—Rid) belongs to the *Rosaceae* family. The plant is widespread in Europe, in the South mainly in the mountainous areas, but it also occurs naturally in North Asia and North America [21]. In folk medicine, leaves were used for diarrhea, period cramps, wounds, sore throats, mouth ulcers, and eye inflammation. The berries were recommended for indigestion and rheumatism, and the raspberry juice has been used as a cooling remedy for fevers, children’s illnesses, and cystitis [12,22]. Recent studies have shown that the consumption of raspberry may be beneficial for woman, as it regulates hormones, improves insulin sensitivity, reduces inflammation, and supports weight management [23]. These properties are due to the high levels of certain phytoestrogens, such as genistein, formononetin, and daidzein [24,25]. These reduce androgen levels in Polycystic Ovary Syndrome (PCOS) patients [24] and can relieve menopausal symptoms [26].

Dog rose (*Rosa canina* L.—Rca) species belong to the *Rosaceae* family. It is a perennial and deciduous shrub that grows in wide areas across Europe, North Africa, and West Asia. Its fruit is a pseudo fruit called rosehip, which is rich in polyphenols, carotenoids, and vitamin C, among other compounds [27]. In folk medicine, its leaves are used for colds, flu, cough, and eczema, while the fruits strengthen the immune system [12,28]. The dog rose tincture is suitable for treating diarrhea, colic pain, and coughing [22]. Studies also reveal that rosehip consumption has antidiabetic properties, reduces cardiovascular diseases by decreasing blood pressure and low-density lipoproteins (LDL), and is effective against rheumatoid arthritis and osteoarthritis, and may reduce the proliferation of cancer cells [27,29,30,31]. The fruit has anti-inflammatory properties due to its high levels of galactolipids (GOPO) and polyunsaturated fatty acids, such as ω-3, ω-6, linoleic and α-linolenic acids [28].

The lingonberry (*Vaccinium vitis-idaea* L.—Vid) species belongs to the *Ericaceae* family. It is an evergreen shrub with red, acid berries, native to northern countries such as Russia, Canada, and northern parts of Europe, with a distribution in the southern mountains [32,33]. The berries belong to the “superfruits”, as they contain high levels of vitamin C, A and E, several bioactive compounds such as polyphenols [34,35], and also benzoic acid in quite a high concentration [36]. The plant is used in folk medicine for its medicinal properties. The leaves are beneficial in cystitis, urinary tract infections, and non-insulin dependent diabetes mellitus [22]. In different countries, people have used the leaves or fruits as analgesics and anti-inflammatory agents, especially in urinary system diseases, rheumatoid arthritis, and gastrointestinal disorders [37]. Studies have shown that lingonberry fruit has antioxidant, anticancer, neuroprotective, antidiabetic, anti-obesity, anti-inflammatory, and antimicrobial activity [38,39,40,41,42,43].

Hawthorn (*Crataegus oxyacantha* L.—Cox) belongs to the *Rosaceae* family. It is a thorny shrub with red berries and is widespread in North America, Europe, and the Himalayas [44]. It is listed in the European Pharmacopoeia [45] as an official medicine for the treatment of heart diseases, heart failure, hypertension, and high blood lipids [46]. In various European countries, people have used the berries, flowers, and bark to treat cardiovascular diseases and nervous system conditions [44]. In addition, the berries have astringent and diuretic properties and are effective against diarrhea, urinary retention, and intestinal spasms [47]. The plant is rich in flavonoids (rutin, quercetin, etc.), terpenoids (oleanolic and ursolic acids), organic acids (malic acid and citric acid) and minerals (K, Ca Mg, P) [44,48]. Studies have shown that it exert several cardiovascular pharmacological properties such as antioxidant activity, positive inotropic, anti-inflammatory, anticardia remodeling, antiplatelet aggregation, vasodilating, endothelial protective, antiarrhythmic, and lipid lowering effects [49]. Other studies have shown that different hawthorn extracts have anticataract potential, radioprotective, anti-atherosclerosis, and anticancer effects [47].

Common grape (*Vitis vinifera* L.—Vvi) belongs to the *Vitaceae* family. It is a perennial, woody climbing plant, native to southern Europe and western Asia [50], but nowadays it is distributed almost all over the world [51]. It is one of the world’s largest fruit crops, with an annual production of over 78 million tons (2020) [52]. Around 80% of the harvest is used for winemaking [51]. In folk medicine, flowers, leaves, tendrils, and berries have been used for medicinal and therapeutic purposes. Grapes were recommended for stimulating bowel and kidney functions. Grape juice was used to treat diarrhea and to prevent kidney, bladder, and gallstones. The oil obtained from grape seeds was recommended to help digestion [53]. In addition to these, several other applications can be found in different countries [13,54]. The main compounds in the common grapes are polyphenols, aromatic acids, and stilbenoids (ex. resveratrol) [50,54,55]. Studies have shown that the parts of the grapevine plant possess antioxidant, cardio, and hepatoprotective properties, while anticarcinogenic, neuro, and dermatoprotective features have also been observed next to antidiabetic and anti-inflammatory aspects [50,51,56,57].

The boxwood (*Buxus sempervirens* L.—Bsv) belongs to the *Buxaceae* family. It is an evergreen shrub or small tree found across Europe, Southern Asia, Africa, North, and Central America, and its versatility has made it very popular in ornamental landscaping [58]. The plant is one of the richest plant species in alkaloids, tannins, saponins, and glycosides [59], and it was used in folk medicine for the treatment of rheumatism, fever, arthritis, malaria, and skin ulcers [60]. In addition, it has purgative, laxative, and antipyretic properties [59], and it can be used for wounds, pain, gout, syphilis, dermatitis, coronary diseases, toothache, hernias, bruises, and chest and abdominal disorders. Studies have shown that due to its lupane triterpene, it could be applied for the treatment of drug-resistant malaria [58]. It has also been shown to have anticancer effects, as in one study acetonic extract induced the death of breast cancer cells via apoptosis and autophagy [61], while in another study the hydroalcoholic extract inhibited the proliferation of melanoma, colorectal carcinoma, prostate cancer and skin fibroblast cells [60].

The GTEs specific to the above-mentioned plant species were prepared and microbially evaluated using identical methodologies, and therefore, such a comparative study could indicate the relative strength of their antimicrobial activity, too. The performed experiments were addressing topics like total phenolic content (TPC), total flavonoid content (TFC), and condensed tannin content (CTC) followed by the antioxidant activities of GTEs as revealed by 2,2-diphenyl-1-picrylhydrazyl (DPPH), Ferric Reducing Antioxidant Power (FRAP), and Trolox Equivalent Antioxidant Capacity (TEAC) methods. More importantly the GTEs specific antimicrobial activity was analyzed by looking at fourteen microbial strains such as *Escherichia coli*, *Pseudomonas aeruginosa*, *Salmonella enterica* subsp. *enterica*, *Bacillus cereus*, *Staphylococcus aureus*, *Enterococcus faecalis*, *Listeria monocytogenes*, *Saccharomyces cerevisiae*, *Candida albicans*, *Aspergillus flavus*, *A. niger*, *A. ochraceus*, *Penicillium citrinum*, and *P. expansum*. To demonstrate the concentration-dependency of an antimicrobial effect that might be specific to GTE, we used agar diffusion, minimum inhibitory concentration (MIC), and minimum microbicidal concentration (MMC) methods. In addition, antibacterial susceptibility tests were conducted to analyze the studied bacterial species using antibiotics like tetracycline, kanamycin, and cefotaxime. This experimental setup would provide further insights into the challenges of antimicrobial resistance.

## 2. Results

Among phytonutrients, polyphenols of flavonoid and non-flavonoid types are considered of relevant importance and could generate multiple physiological effects [62]. Moreover, the phytonutrient profile of some GTEs revealed anti-inflammatory, antidiabetic and antimicrobial effects that have been related to their polyphenol content [2,5,6,7].

We carried out qualitative and quantitative evaluations to analyze whether the polyphenol content could be correlated with the antimicrobial effects of some GTEs.

### 2.1. Comparative Analyses of GTEs Specific Total Phenolic Content (TPC), Total Flavonoid Content (TFC) and Condensed Tannin Content (CTC)

**Figure 1:**
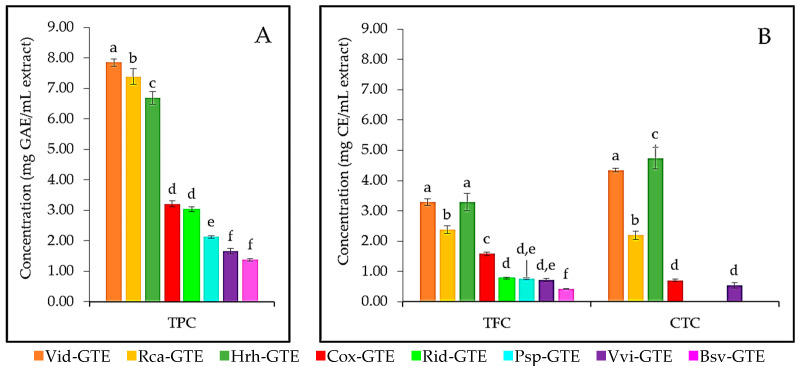
(**A**) The total phenolic (TPC), (**B**) total flavonoid (TFC) and condensed tannin content (CTC) specific to the studied GTEs: blackthorn (Psp-GTE), boxwood (Bsv-GTE), common grape (Vvi-GTE), dog rose (Rca-GTE), hawthorn (Cox-GTE), lingonberry (Vid-GTE), raspberry (Rid-GTE) and sea buckthorn (Hrh-GTE); GAE—gallic acid equivalent, CE—catechin equivalent. Values with different letters (a–f) within one method are statistically different at *p* < 0.05, according to Tukey’s test.

In the present study, we determined the total phenolic, flavonoid, and condensed tannin content of eight GTEs, including blackthorn (Psp-GTE), boxwood (Bsv-GTE), common grape (Vvi-GTE), dog rose (Rca-GTE), hawthorn (Cox-GTE), lingonberry (Vid-GTE), raspberry (Rid-GTE) and sea buckthorn (Hrh-GTE), (for details see Materials and Methods). The results for TPC and TFC are shown in Figure 1. Interestingly, the lingonberry (Vid), dog rose (Rca), and sea buckthorn (Hrh) GTEs were the richest sources of polyphenols, containing four to two times more polyphenols than the other GTEs (Figure 1). The hawthorn (Cox), raspberry (Rid), blackthorn (Psp), common grape (Vvi) and boxwood (Bsv) type of GTEs showed decreasing TPC values compared to the lingonberry (Vid), dog rose (Rca), and sea buckthorn (Hrh) GTEs.

The TFC followed an almost identical trend with the TPC (see Figure 1A). Statistical analyses showed that in the case of TPC there were no significant differences between hawthorn (Cox)—raspberry (Rid) and the common grape (Vvi)—boxwood (Bsv) pairs of GTEs. Furthermore, in case of TFC, the GTE pairs like lingonberry (Vid)—sea buckthorn (Hrh), raspberry (Rid)—blackthorn (Psp), and blackthorn (Psp)—common grape (Vvi) would contain similar concentration of flavonoid. Similarly to TFC, GTEs like lingonberry (Vid), sea buckthorn (Hrh), and dog rose (Rca) exhibited the highest tannin concentration. Hawthorn (Cox) and common grape (Vvi) GTEs had significantly similar tannin content. However, raspberry (Rid), blackthorn (Psp), and boxwood (Bsv) GTEs did not contain detectable amounts of tannins.

The highest TPC and TFC content was found in lingonberry (Vid) GTE with 7.85 mg GAE/mL and 3.28 mg CE/mL, respectively, and it features the second highest CTC with 4.34 mg CE/mL. The dog rose (Rca) GTE showed the second highest TPC with 7.39 mg GAE/mL, and the third highest TFC and CTC with 2.39 mg CE/mL and 2.20 mg CE/mL, respectively. The sea buckthorn (Hrh) GTE showed the third highest TPC (6.69 mg GAE/mL), while the second highest TFC (3.29 mg CE/mL) and the highest CTC with 4.74 mg CE/mL.

### 2.2. The Specific Antioxidant Potential Evaluation of the Investigated GTEs

Polyphenols are known for their antioxidant properties, and a rich polyphenol content has been observed to generate pronounced antioxidant capacity and eventual antimicrobial activity [63]. It has also been shown that the richer the flavonoid and polyphenol content, the more pronounced the antimicrobial effect [64]. To evaluate the antioxidant capacity of the analyzed GTEs, several methods, such as DPPH, FRAP, and TEAC, were employed to determine the direct or indirect antioxidant properties of specific polyphenols (see Section 4). Direct antioxidants have redox activity that must be regenerated. Indirect antioxidants have variable redox properties and activate the Keap1/Nrf2/ARE pathway, which induces the expression of phase 2 cytoprotective enzymes. The performed antioxidant tests partially shed light on the dual protective role of indirect antioxidants: the prompt, direct scavenging of hazardous oxidants, and the induction of an antioxidant cellular response. The latter requires genomic and biochemical studies [65].

**Figure 2:**
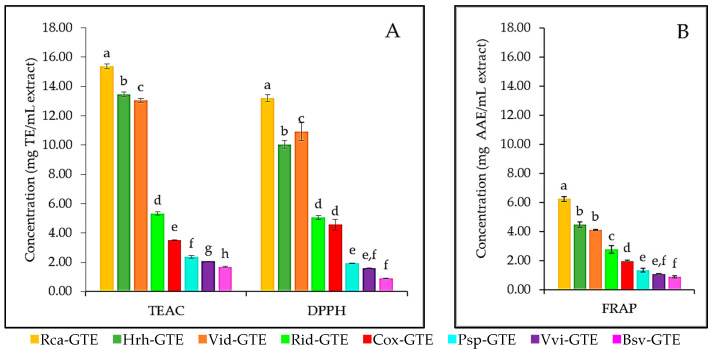
Evaluation of GTE-specific antioxidant potential using three methods for blackthorn (Psp) GTE, boxwood (Bsv) GTE, common grape (Vvi) GTE, dog rose (Rca) GTE, hawthorn (Cox) GTE, lingonberry (Vid) GTE, raspberry (Rid) GTE, and sea buckthorn (Hrh) GTE. (**A**) TEAC and DPPH methods, (**B**) FRAP method; TE—Trolox equivalent, AAE—Ascorbic acid equivalent. Values with different letters (a–h) within one method are statistically different at *p* < 0.05, according to Tukey’s test.

All three methods used to study the antioxidant potential demonstrated that the dog rose (Rca), lingonberry (Vid), and sea buckthorn (Hrh) GTEs showed the highest antioxidant capacity, while blackthorn (Psp), common grape (Vvi), and boxwood (Bsv) GTEs featured the most reduced antioxidant activities, which would be 6–10 times less than the dog rose (Rca) GTE (see Figure 2). The dog rose (Rca) GTE displayed values like 15.36 mg TE/mL for TEAC and 13.2 mg TE/mL for DPPH, while 6.24 mg AAE/mL for FRAP. Lingonberry (Vid) GTE shared second/third place with the highest values at 13.05 mg TE/mL for TEAC and 10.91 mg TE/mL for DPPH, while 4.10 mg AAE/mL for FRAP. The second/third highest antioxidant capacities were also seen in the case of the sea buckthorn (Hrh) GTE at, and for TEAC (13.47 mg TE/mL), DPPH (10.04 mg TE/mL), and FRAP (4.47 mg AAE/mL). As seen on Figure 2, all other GTEs have a significantly (less than 50%) lower concentration of antioxidants.

In the case of the DPPH method, there is no significant difference between the antioxidant capacity of raspberry (Rid) and hawthorn (Cox), and common grape (Vvi) and blackthorn (Psp) GTEs. The FRAP method did not reveal significant differences among GTE pairs like sea buckthorn (Hrh)—lingonberry (Vid), common grape (Vvi)—blackthorn (Psp) and blackthorn (Psp)—boxwood (Bsv) featuring similar antioxidant capacities (see Figure 2B). Moreover, in the case of the raspberry (Rid) GTE, almost identical antioxidant potential was recorded by TEAC and DPPH methods.

### 2.3. Phytochemical Composition of GTEs

Generally, the main compounds identified are caffeic acid and chlorogenic acid, as well as quercetin and its derivatives, hyperoside and rutoside. Each GTE has its own specific characteristics. The Psp-GTE sample proved to be the richest in the bioactive components examined, particularly chlorogenic acid, catechin, and rutoside. The Rid-GTE and Vid-GTE samples stood out for their high ellagic acid content, while Cox-GTE showed a favorable phenolic profile mainly due to its chlorogenic acid and rutoside content. The other samples (Hrh-GTE, Rca-GTE, Bsv-GTE, Vvi-GTE) contained bioactive components in smaller amounts (see Table 1, for chromatograms and the corresponding MS spectra, see Supplementary Material).

One of the most prominent components of Rid-GTE is ellagic acid (2.246 mg/mL), which is accompanied by moderate amounts of chlorogenic acid and rutoside.

Psp-GTE contained exceptionally high concentrations of chlorogenic acid (7.546 mg/mL), rutoside (6.385 mg/mL) and caffeic acid (1.759 mg/mL). In addition, significant amounts of quercetin, hyperoside, and several other flavonoids were also detected in significant amounts.

The composition of Hrh-GTE was more modest, but the presence of catechin (5.849 mg/mL) and carnosic acid (1.009 mg/mL) is significant. Moderate concentration of caffeic acid and rutoside is worth mentioning.

Several phenolic compounds were found in low concentrations in the Rca-GTE, including hyperoside (0.785 mg/mL), chlorogenic acid (0.476 mg/mL), catechin (0.443 mg/mL) and rutoside (0.347 mg/mL).

One characteristic of Vid-GTE is the presence of ellagic acid (2.084 mg/mL). In addition, it contained measurable amounts of chlorogenic acid (1.680 mg/mL), hyperoside (1.446 mg/mL), arbutoside (1.330 mg/mL) and rutoside (1.210 mg/mL), giving Vid-GTE a balanced phenolic profile.

Cox-GTE contained a relatively high concentration of chlorogenic acid (3.865 mg/mL), hyperoside (1.822 mg/mL), and rutoside (0.902 mg/mL).

Bsv-GTE contained smaller amounts of phenolic compounds, but it is worth noting the presence of rutoside (0.608 mg/mL), hyperoside (0.178 mg/mL), and chrysin (0.118 mg/mL).

Several compounds were detected in low concentrations in Vvi-GTE, such as caffeic acid (0.813 mg/mL), quercetin (0.413 mg/mL), and rutoside (0.378 mg/mL).

### 2.4. Antimicrobial Activity Evaluation of GTEs

#### 2.4.1. Antimicrobial Activity of GTEs as Revealed by the Agar Diffusion Method (ADM)

The agar diffusion method (ADM) was used to measure the size of the inhibition zones as described in the Materials and Methods. Table 2 shows the inhibition zones that were specific for different GTE concentrations in the case of the assessed Gram-positive bacterial species. Interestingly, the tested Gram-negative bacteria, yeast, and mold species were resistant to the GTEs used, as no inhibition zones were observed.

**Figure 3:**
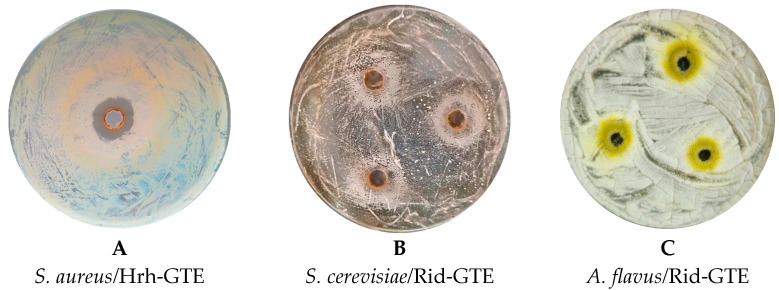
The different sizes of the inhibition zones induced by the GTEs on some tested microorganisms.

According to our data, the most effective GTEs in terms of number of growths inhibited (number of microorganisms) were dog rose (Rca), lingonberry (Vid), sea buckthorn (Hrh), and hawthorn (Cox) GTEs, which were found to be effective against *S. aureus*, *B. cereus*, *E. faecalis*, and *L. monocytogenes*, even at the lowest extract concentrations (see Table 2 for details). Among bacteria, *S. aureus* was the most affected strain out of all the studied ones in this research, while six out of eight GTEs showed certain inhibition at various concentrations (see Figure 3A). The lingonberry (Vid) GTE was even effective at the lowest concentration of 10%. The dog rose (Rca) and hawthorn (Cox) GTEs showed the inhibition at the lowest 20% concentration. Two of the extracts, the boxwood (Bsv) and common grape (Vvi) GTEs, exhibited no inhibition even at the highest concentrations.

The second most affected Gram-positive bacteria was *B. cereus*, where six out of eight GTEs showed some inhibition potential, albeit sometimes at higher concentrations. The dog rose (Rca) GTE proved the most promising since even at 10% concentration exhibited inhibitory effect. Two of the extracts, the raspberry (Rid) and boxwood (Bsv) GTEs had absolutely no effect on this bacterium. However, it should be noted that the common grape (Vvi) GTE seems not to interfere with any of the tested bacteria; the only exception is its 100% concentration, which is able to inhibit *B. cereus.*

*E. faecalis* was affected by roughly 50% of all the examined GTEs, showing complete resistance to raspberry (Rid), blackthorn (Psp), boxwood (Bsv) and common grape (Vvi) GTEs. Once again, the lingonberry (Vid) GTE proved to be the most effective, whereas a concentration as low as 30% showed an inhibitory effect during our experiment. Two of the extracts, like sea buckthorn (Hrh) and dog rose (Rca) GTEs showed effectiveness at 50% concentration, and hawthorn (Cox) GTE featured inhibitory potential at a 60% concentration.

Among the tested Gram-positive bacteria, the *L. monocytogenes* seemed to be the most resistant towards the assessed GTEs (see Table 2). Only three of them were effective, and these were, in order of their antimicrobial strength the dog rose (Rca), lingonberry (Vid) and the sea buckthorn (Hrh) GTEs. The dog rose (Rca) GTE showed the most relevant antimicrobial potential as the lowest inhibition concentration was 10%, but some bacterial outgrowth was apparent in the inhibition zone, and therefore, it cannot be considered as a perfect inhibition. While sea buckthorn (Hrh) GTE was effective, it showed inhibitory potential only at 100% concentration, but the lingonberry (Vid) GTE was more effective starting from 40% concentration. All other GTEs provided no inhibition zones for *L. monocytogenes*.

Statistical tests often show that there is no statistical difference between different concentrations within a sample, so for example, for *S. aureus* and dog rose (Rca) GTE, the inhibition zones of 100, 90 and 80% were statistically identical, meaning that any of the three concentrations can be used and the resulting inhibitory effect will be almost identical. It seems likely that the antimicrobial components present in the extracts can behave equally efficiently up to a given dilution.

#### 2.4.2. Testing of the Antimicrobial Activity of GTEs Against Yeast and Mold Using the Agar Diffusion Method (ADM)

The antimicrobial activity of the studied GTEs on various yeast and mold species was assessed similarly to bacteria, and several interesting phenomena were observed (see Table 3).

Interestingly, no clear inhibition zones were evident. In some cases, they appeared to be replaced by a premature sporulation region (see Figure 3C). *S. cerevisiae* growth was supported by 100% raspberry (Rid) GTE in the 24.55 mm zone (Figure 3B). In the case of the *Aspergillus* species, instead of an inhibition, a spore zone was observed around the agar hole, where the spore formation started earlier than elsewhere (Figure 3C). This means that although GTE did not have a proper antimicrobial effect, it most likely induced spore formation through an unfavorable environmental change, causing them to sporulate much earlier. No antimicrobial or premature sporulation effects were observed for the *Penicillium* species or *Candida albicans*.

In the case of *A. niger*, the antifungal effect was evident for blackthorn (Psp), dog rose (Rca), hawthorn (Cox) and raspberry (Rid) GTEs (Table 3). All aforementioned extracts lead to premature sporulation, but to a variable extent and forming a lot of spores.

*A. ochraceus* was susceptible to three extracts: blackthorn (Psp-GTE), boxwood (Bsv-GTE), and hawthorn (Cox-GTE). Despite sporulation induction, many spores were formed only with blackthorn (Psp) GTE (Table 3).

*A. flavus* was the least affected mold species. It was inhibited by only the raspberry (Rid) and dog rose (Rca) GTEs. Both also assisted in premature sporulation, albeit to a lesser extent. The remaining studied GTEs did not exhibit any antifungal effects on the mold species examined, as revealed by the agar diffusion assay.

#### 2.4.3. Assessing GTEs Minimum Inhibitory Concentration (MIC) and Minimum Microbicidal Concentrations (MMC)

The agar diffusion method is suitable for assessing microbial growth. However, it is a qualitative assay and cannot be used to quantify the antimicrobial activity of a substance/extract based on the size of the inhibition formed during analysis. Although diffusion techniques can be used for antimicrobial screening, they should never be considered a definitive method [66]. This inconvenience was overcome by using the broth microdilution method (BMM) to determine the minimum inhibitory and microbicidal concentrations. The minimum inhibitory concentration (MIC) is the lowest concentration of an antimicrobial agent (in our case the GTE) that inhibits the visible growth of the microorganism after an overnight incubation. Minimum microbicidal concentration (MMC) refers to the lowest concentration of an antimicrobial agent that prevents the growth of the organism after subculture onto antibiotic-free media [67]. The minimum microbicidal concentration may be referred to as the minimum bactericidal concentration (MBC) or the minimum fungicidal concentration (MFC), depending on the microorganisms assessed.

##### Minimum Inhibitory Concentrations (MIC) of GTEs

**Figure 4:**
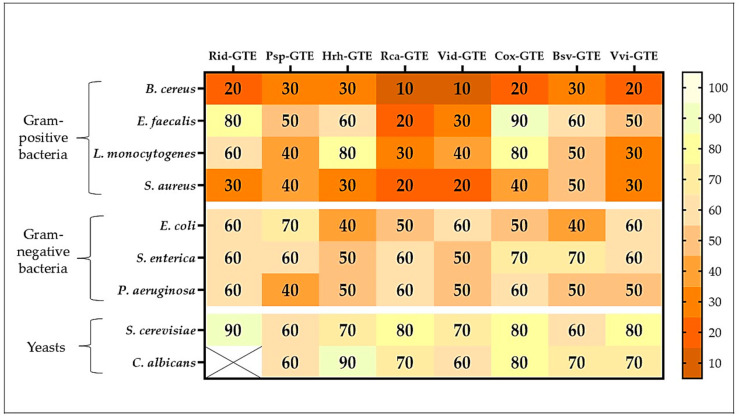
The MIC (%) of GTEs against Gram-positive, Gram-negative bacteria, and yeasts. (x: no effects, the darker the color, the more effective the extract). The % represents dilutions of a stock type of GTE.

The studied GTEs exhibited various inhibition effects (see Figure 4). Raspberry (Rid) GTE was effective against *B. cereus* and *S. aureus* even at low concentrations, with as little as 20–30% extract inhibiting microbial growth. Inhibition was generally observed above a 60% concentration for Gram-negative bacteria, while no or only slight inhibition was observed for yeasts. The growth of the yeast *Candida albicans* was not inhibited at all by the raspberry (Rid) GTE.

A concentration of 30–40% blackthorn (Psp) GTE effectively inhibited the growth of *B. cereus*, *L. monocytogenes*, *S. aureus*, and *P. aeruginosa*. However, extracts only above 60% produced a similar effect on yeasts.

The sea buckthorn (Hrh) GTE inhibited the growth of *B. cereus*, *S. aureus*, and *E. coli* at concentrations of 30% and 40%, while extracts at concentrations higher than 50% were required for the other microbes.

Among the Gram-positive bacteria tested, the dog rose (Rca) and lingonberry (Vid) extracts were the most effective, with even low concentrations (10–30%) inhibiting microbial growth.

Hawthorn (Cox) GTE was only effective against *E. faecalis*, *L. monocytogenes*, *S. enterica*, and yeasts at concentrations of 70–80%, which cannot be considered efficient. For *B. cereus*, the 20% extract was effective, but for the other bacteria, only the concentrations of 40% and above were effective.

Overall, boxwood (Bsv) GTE showed moderate efficacy, with concentrations of 40–60% and above having an inhibitory effect.

The common grape (Vvi) GTE was considered moderately effective. Extract concentrations above 30% were effective against Gram-positive bacteria, above 50% against Gram-negative bacteria, and above 70% against yeasts.

As shown in Figure 4, a 30% concentration of blackthorn (Psp), sea buckthorn (Hrh), and boxwood (Bsv) GTEs inhibited the growth of Gram-positive bacteria, such as *B. cereus*. For this bacterium, an inhibitory effect was observed with 20% raspberry (Rid), hawthorn (Cox), and common grape (Vvi) GTEs, while dog rose (Rca) and lingonberry (Vid) GTEs were effective at concentrations as low as 10%. The concentration requirements are much higher for *E. faecalis*. The highest concentrations are required for GTEs with hawthorn (Cox) (90%) and raspberry (Rid) (80%). The lowest concentration required was for the dog rose (Rca) GTE, for which inhibition was observed at 20%. *L. monocytogenes* exhibits high resistance to most GTEs. Extracts with sea buckthorn (Hrh) and hawthorn (Cox) inhibited growth at a concentration of 80%, while dog rose (Rca) and common grape (Vvi) GTEs exhibited the same effect at a concentration of 30%. Similarly, in the case of *S. aureus*, even lower concentrations of GTEs were found to be effective. While extracts of dog rose (Rca) and lingonberry (Vid) were effective at concentrations as low as 20%, most other extracts at concentrations of 30–40% and boxwood (Bsv) GTE was only effective at 50%.

In general, Gram-negative bacteria showed higher resistance to GTEs. No GTE was effective against *E. coli* at concentrations below 40%, at which concentration extracts of sea buckthorn (Hrh) and boxwood (Bsv) were effective. Dog rose (Rca) and hawthorn (Cox) GTEs were effective at concentrations of 50%, raspberry (Rid), lingonberry (Vid), and common grape (Vvi) GTEs were effective at 60% or higher while blackthorn (Psp) GTE was effective at 70% or higher concentration.

*S. enterica* was even less sensitive to GTEs. Sea buckthorn (Hrh) and lingonberry (Vid) GTEs were effective at concentrations of 50% or higher, while hawthorn (Cox) and boxwood (Bsv) GTEs were only effective at concentrations above 70%.

In the case of *P. aeruginosa*, the required concentrations were slightly lower. Blackthorn (Psp) GTE was effective at a concentration of 40%, while raspberry (Rid), dog rose (Rca), and hawthorn (Cox) GTEs were only effective at concentrations above 60%.

In the case of yeasts, the required concentrations were even higher, and no extract was effective below 60%. Surprisingly, the raspberry (Rid) GTE had a positive effect on the *C. albicans* species, showing synergistic effects and promoting growth. Boxwood (Bsv), blackthorn (Psp) and lingonberry (Vid) GTEs exhibited inhibitory effects at concentrations as low as 60%, while hawthorn (Cox), sea buckthorn (Hrh), and dog rose (Rca) GTEs were effective at concentrations above 80%.

##### Minimum Microbicidal Concentrations (MMC) of the Studied GTEs

**Figure 5:**
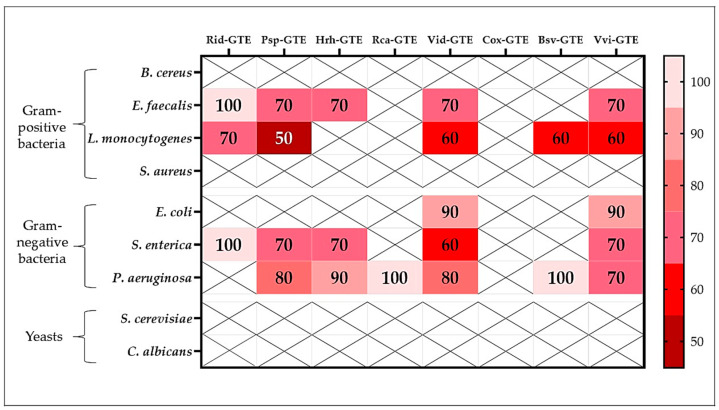
The MBC (%) of GTEs against Gram-positive, Gram-negative bacteria, and yeasts. (x: no effect; the darker the color, the more effective the extract). The % represents dilutions of a stock type of GTE.

The microbicidal effect was insignificant; none of the GTEs produced bactericidal effects (directly killing of bacteria) on *B*. *cereus*, *S. aureus*, *S. cerevisiae* and *C. albicans* (see Figure 5).

The most effective extracts from this point of view were from blackthorn (Psp), lingonberry (Vid), and common grape (Vvi), as concentrations of 60–70% produced a bactericidal effect on *E. faecalis*, *L. monocytogenes*, *E. coli*, *S. enterica*, and *P. aeruginosa*. The next most effective extract in the series was sea buckthorn (Hrh), which destroyed *E. faecalis* and *S. enterica* at a concentration of 70% and *P. aeruginosa* at 90%. A 70% concentration of raspberry (Rid) GTE was lethal to *L. monocytogenes* bacteria. However, the concentrated Rid-GTE had a bactericidal effect on *E. faecalis* and *S. enterica*. The dog rose (Rca) and hawthorn (Cox) GTEs were the least effective in this regard, demonstrating virtually no microbicidal activity against any of the tested microbes.

#### 2.4.4. Antimicrobial Susceptibility of Studied Microbial Strains

**Figure 6:**
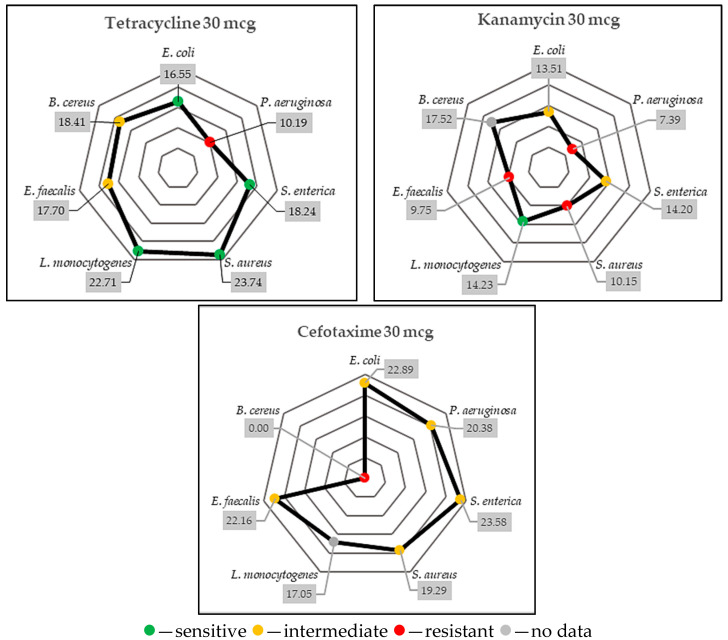
Evaluation of the antimicrobial susceptibility based on our results and the zone size interpretative table (Table A1) (mm).

In addition to the identification of antimicrobial GTEs, it would be equally important to verify the antimicrobial susceptibility of the tested microbial strains. Considering the obtained results, the specificity of the antimicrobial effects could be deducted for the analyzed GTEs, but it is equally important to study the antimicrobial susceptibility of the analyzed bacterial strains. Testing for antimicrobial susceptibility to certain antibiotics increases our confidence in the accuracy of our results. Such data could further improve the use of current antibiotics. Three common antibiotics (tetracycline, kanamycin, and cefotaxime) were used to determine the susceptibility of the tested bacterial strains used. Manufacturers of antibiotic discs have defined inhibition zone intervals that can be used to determine if a bacterium is sensitive, intermediate, or resistant to a particular antibiotic (See Appendix A). The size of the zone of inhibition provides information on the susceptibility of the bacteria. The magnitude of the zone directly correlates with the degree of sensitivity exhibited by the microbe to the antibiotic. Figure 6 shows the relationship between the seven types of bacteria studied (*S. aureus*, *L. monocytogenes*, *E. faecalis*, *B. cereus*, *E. coli*, *P. aeruginosa*, *S. enterica*) and the three antibiotics of choice.

The tetracycline resistance was visible for *P. aeruginosa*, while kanamycin inefficiency was seen in case of *P. aeruginosa*, *S. aureus*, and *E. faecalis* and the cefotaxime resistance was evident for *B. cereus.*

According to Table A1, *E. coli* (>15 mm), *S. enterica* (>15 mm), *S. aureus* (>19 mm), and *L. monocytogenes* (>13.8 mm) were sensitive to tetracycline, while intermediate effects were observed against *E. faecalis* (15–18 mm) and *B. cereus* (19–22 mm). Kanamycin showed good antimicrobial activity against *L. monocytogenes* (>11 mm), and it had intermediate effects against *E. coli* (14–17 mm) and *S. enterica* (14–17 mm). Cefotaxime showed intermediate effects against almost all bacteria, except *B. cereus*, which was resistant to it. These results are very important for our research because they allow us to test the efficacy of the assessed GTEs against some antibiotic-resistant strains.

This experiment demonstrated that the microbial strains studied exhibited varying levels of susceptibility to antimicrobials. This calls into question the relevance of antimicrobial studies in the context of antibiotic resistance. Did the tested microbe acquire antibiotic resistance before or during the experiment? Answering this question might be relevant for the antimicrobial intervention.

## 3. Discussion

Antibiotic resistance and antimicrobial susceptibility are major problems requiring efficient solutions, for which natural antimicrobial agents like GTEs could be a plausible option. Although many searches have investigated the antimicrobial activity of different plant parts, the study of GTEs is still in its early yet promising stage. Therefore, our study focuses on identifying GTEs and their phytonutrient profiles relevant to antimicrobial properties that may be either microbiostatic or microbicidal.

### 3.1. The Dog Rose (Rca), Sea Buckthorn (Hrh), and Lingonberry (Vid) GTEs Present Substantial Polyphenol Content and Antioxidant Activity, While Other GTEs, Including Blackthorn (Psp) and Common Grape, Lag Behind

The polyphenols are secondary metabolites of plants and are considered bioactive compounds with health benefits, such as antioxidant, anti-inflammatory, and antimicrobial effects [68]. Therefore, the analysis of any GTE-specific polyphenol content could provide a better understanding of their presumptive health-promoting properties. Considering its specific strengths and limitations, three methods were applied to determine the antioxidant capacity, since these methods would only reveal a limited part of the total capacity. The DPPH method is only suitable for the detection of small hydrophobic antioxidants as the limited space around the nitrogen atom sterically inhibits the binding of bulk radicals to this region. Another limitation is that DPPH radical can interact with other radicals, and carotenoids may interfere at the measurement wavelength. The TEAC method is useful for measuring both hydrophilic and lipophilic antioxidants. The FRAP method is suitable for measuring hydrophilic antioxidants, but substances with a lower redox potential than Fe^3+^/Fe^2+^ interfere with the measurement and prevent quantification of antioxidants with a –SH group [69].

Our study has shown the lingonberry (Vid), dog rose (Rca) and sea buckthorn (Hrh) GTEs featuring the highest, while the other assessed GTEs except for blackthorn (Psp) would contain significantly lower amounts of TPC, TFC, and CTC values (see Figure 1). The blackthorn (Psp) was shown not to contain CT. It was also observed that the CTC values were higher than the flavonoid content in some cases, but this has also been found by other authors [70,71,72]. There could be several reasons for this phenomenon. For instance, the aluminum chloride colorimetric method was not able to measure all flavonoids, so the method slightly underestimates the total flavonoid content [73]. In contrast, the vanillin-HCl assay measures more than just condensed tannins, which may lead to an overestimation of the actual tannin content [74,75].

However, when antioxidant capacity was also evaluated using the DPPH, TEAC, and FRAP methods, the relative order of the GTEs remained almost entirely unchanged in the case of TPC and TFC (see Figure 2), with dog rose (Rca) and sea buckthorn (Hrh) GTEs ranked first and second with the highest antioxidant potential, while lingonberry (Vid) GTE ranked third.

The antioxidant potential revealed through the TEAC method is slightly higher than those being measured with the DPPH method, indicating that the extracts also contain some lipophilic antioxidants. The results of the FRAP method indicate that the concentration of hydrophilic antioxidants is lower than that of hydrophobic antioxidants. Moreover, the relative order of the GTEs regarding their antioxidant capacity, as assessed by the three methods, was identical. The discrepancy between the polyphenol/flavonoid content and the antioxidant potential of lingonberry (Vid), dog rose (Rca), and sea buckthorn (Hrh) GTEs could imply several explanations. In the case of lingonberry (Vid) GTE, it is possible that some of the polyphenols would not exhibit antioxidant properties. It is also plausible that dog rose (Rca) GTE and sea buckthorn (Hrh) GTE could contain additional phytonutrients that do not fall into the polyphenol/flavonoid category with antioxidant properties.

Moreover, some differences were found in the polyphenol and flavonoid content of dog rose (Rca), lingonberry (Vid), and sea buckthorn (Hrh) when specific plant parts were examined. Polumackanycz et al. (2020) analyzed different varieties of dog rose leaf and berry extracts. In their study the antioxidant capacity of berries and leaves varied between 2–21 TE/100 g and 18–35 mg TE/100 g, respectively, while the polyphenol content was 56–177 mg GAE/g for leaves and 44–157 mg GAE/g for berries [76]. In the study of Papuc et al. (2013), the polyphenol content of ethanolic dog rose fruit extract was 220 mg GAE/100 mL, which is significantly lower than the dog rose (Rca) GTE in our study [77].

As reported by Wang et al. (2005), the acetone lingonberry red fruit extract exhibited a relatively low polyphenol content of 663 mg GAE/100 g [78], while in another study the concentrated juice contained only 3.7 mg GAE/100 g [79]. Our results showed that the Vid-GTE had a higher polyphenol content, indicating that fruit is not the best source of polyphenols in the lingonberry plant.

Criste et al. (2020) have analyzed different varieties of sea buckthorn leaf and berry extracts. The TPC values ranged between 10–20 mg GAE/g and 40–50 mg GAE/g in case of berries and leaves, respectively [80]. In our study, the Hrh-GTE displayed a higher content of polyphenols compared to leaves and berries, and similarly to the findings of Criste and their colleagues, most of these polyphenols were of the flavonoid type. Other extracts from roots, bark, and leaves showed similar tendencies in terms of their polyphenol and flavonoid contents [81,82]. The antioxidant capacities of Hrh-GTE were 90, 200, and 270 mg/g for FRAP, DDPH, and TEAC assays, respectively. According to the literature data, among other plant tissues, leaves had the highest antioxidant content with 120–140 mg/g DPPH, and 100–120 mg/g TEAC values [80].

Regarding the composition of different plant parts, no comparative studies have been performed on blackthorn and common gape, though some differences are predictable.

In conclusion, our data indicates that the analyzed GTEs obtained from the buds of dog rose (Rca), sea buckthorn (Hrh), and lingonberry (Vid) do contain higher concentrations of polyphenols and exhibit greater antioxidant potential than other parts of the same plant species.

### 3.2. Antimicrobial Activity of GTEs Varies Along a Broad Spectrum

#### 3.2.1. The Dog Rose (Rca) GTE Appears to Be an Efficient Bacteriostatic Antimicrobial

Dog rose (Rca) GTE showed a relevant antimicrobial potential for almost all the bacteria, forming inhibition zones at even the lowest concentrations for *S. aureus*, and starting from 20% dilution for *B. cereus*, *E. faecalis*, and *L. monocytogenes* (see Table 2). It displayed limited antifungal results among the tested yeast and mold species. Against *A. niger* and *A. flavus* it proved somewhat effective, inducing sporulation zones measured at 23.72 ± 0.79 mm and 11.72 ± 0.18 mm, while on *S. cerevisiae* and *A. ochraceus* no inhibitory effects were visible.

The BMM revealed efficient microbiostatic effects against Gram-positive bacteria, with concentrations of 10%, 20%, and 30% acting as the MIC for *B. cereus*, *E. faecalis*, and *S. aureus*, respectively, and for *L. monocytogenes*, respectively. Gram-negative bacteria were found to be more resistant to the dog rose (Rca) GTE. The minimum effective concentrations were 50% for *E. coli*, 60% for *S. enterica*, and 100% for *P. aeruginosa*. The dog rose (Rca) GTE was also effective against *C. albicans* at 70% and *S. cerevisiae* at 80%. Interestingly, the minimum microbicidal effect of dog rose (Rca) GTE is almost nonexistent for most of the microorganisms studied, being only effective against *P. aeruginosa* at a 100% concentration. All together, these results could be anticipated, as previous antimicrobial studies had also proven the effectiveness of dog rose fruit extract against *S. aureus*, *E. faecalis*, *B. cereus*, *B. subtilis*, *E. coli*, *S. enteritidis*, *E. aerogenes*, *P. aeruginosa*, and *C. albicans* [83,84]. Furthermore, the rosehip tea was effective against some clinical bacterial strains such as *P. aeruginosa*, *A. baumannii*, *E. coli*, *K. pneumoniae*, *E. faecalis*, and methicillin-susceptible and multidrug-resistant *S. aureus* (MSSA and MRSA) [85]. According to the literature, rosehip is a rich source of condensed tannins, which can play a significant role in antimicrobial activity [86,87]. Crude flavonoid extracts from a hydroalcoholic extract of dog rose demonstrated inhibitory effects against *E. coli* and *S. aureus* [88]. Furthermore, our studies on the dog rose (Rca) GTE showed to contain the highest quantity of polyphenols, the second largest quantity of tannins and exhibited exceptional antioxidant capacity (TEAC, DPPH, and FRAP), might suggest a putative correlation between these features and the bacteriostatic activity. According to the LC/MS method it contains moderate concentration of hyperoside, chlorogenic acid, catechin, and rutoside, which can contribute to the antimicrobial activity [89].

#### 3.2.2. The Lingonberry (Vid) GTE Features the Most Pronounced Antimicrobial Effect

Lingonberry (Vid) GTE exhibited substantial antimicrobial potential against all Gram-positive bacteria. It formed inhibition zones at the lowest concentrations against *S. aureus* (10%, 9.85 ± 0.24 mm), and at concentrations of 30% for *E. faecalis* (10.33 ± 0.13 mm), *B. cereus* (11.61 ± 0.3 mm), and *L. monocytogenes* (40%, 9.48 ± 0.23 mm). Unfortunately, lingonberry (Vid) GTE was ineffective against yeasts and molds.

The MIC assay revealed an efficient bacteriostatic effect for Gram-positive bacteria, even 10% concentration acted as the minimum inhibitory concentration for *B. cereus*, 20% for *S. aureus*, 30% for *E. faecalis*, and 40% for *L. monocytogenes*.

As with dog rose (Rca), Gram-negative bacteria were slightly more resistant to lingonberry (Vid) GTE. A minimum concentration of 50% was required for *S. enterica* and *P. aeruginosa* to be effective, while *E. coli* required at least 60%. The lingonberry (Vid) extract was also effective at somewhat lower concentrations than the dog rose (Rca) GTE against *C. albicans* (60%) and *S. cerevisiae* (70%). Unlike dog rose (Rca) GTE, the lingonberry (Vid) GTE exhibited microbicidal activity against nearly all the studied microorganisms. At higher concentrations, they were effective against *S. enterica* and *L. monocytogenes* (60%), *E. faecalis* (70%), *P. aeruginosa* (80%), and *E. coli* (90%).

Several studies have demonstrated the relevant antimicrobial potential of lingonberries. Others have found that the fruit extract is effective against *S. cerevisiae*, *A. niger*, *E. coli*, *Serratia marcescens*, *Proteus mirabilis*, *Bacillus subtilis*, *Bifidobacterium* species, and *Clostridium* species [38]. Commercially available lingonberry concentrate inhibited the growth of fungi such as *Absidia glauca*, *Penicillium brevicompactum*, *Saccharomyces cerevisiae*, *Zygosaccharomyces bailii*, *Penicillium*, and *Eurotium* species [40]. Furthermore, lingonberry supernatant was effective against *P. aeruginosa*, *E. coli*, *S. aureus*, *B. cereus*, *S. marcescens*, *B. subtilis*, *Pseudomonas fluorescens*, *L. monocytogenes* [41]. The lingonberry (Vid) GTE performed best among TPC, TFC, and CTC, and had the third highest antioxidant potential. Condensed tannins are the most important type of flavonoids with procyanidin A and B oligomers present in the lingonberry. Ho et al. studied the antimicrobial properties of a tannin extract derived from lingonberries. This extract exhibited antibacterial activity against pathogens such as *Porphyromonas gingivalis* and *Prevotella intermedia* [39]. Other research carried out by Kylli et al. reported strong antimicrobial effects against *S. aureus* using polymeric tannin extracts from lingonberry; however, there were no other bacteriostatic or bactericidal effects found [90]. In another study, ethyl leaf extracts showed very high tannin level and high antimicrobial activity against clinical and multidrug-resistant *S. aureus* strains, combined with antibiofilm formation properties [43].

Therefore, it is important to note that the lingonberry (Vid) GTE specific phytonutrient content and antioxidant potential seem to support both bacteriostatic and microbicidal types of antimicrobial activities efficiently. According to the LC/MS method, it contains a relatively high concentration of ellagic acid, chlorogenic acid, hyperoside, arbutoside, and rutoside, which can contribute to the antimicrobial activity [89,91,92,93].

#### 3.2.3. The Sea Buckthorn (Hrh) GTE Acts Like Another Efficient Antimicrobial

The studied sea buckthorn (Hrh) GTE was effective against some Gram-positive bacteria, forming inhibition zones at a concentration of 40%. For *B. cereus* and *S. aureus*, the zones measured 10.71 ± 0.13 mm and 10.06 ± 0.6 mm, respectively. No antimicrobial activity was detected against Gram-negative bacteria and fungi species.

The MIC results appeared more promising. Among the Gram-positive bacteria, a concentration of 30% is the MIC for *S. aureus* and *B. cereus*, 60% for *E. faecalis*, and 80% for *L. monocytogenes*. For Gram-negative bacteria, the MIC values ranged from 40% to 50%, with 40% for *E. coli* and 50% for *P. aeruginosa* and *S. enterica*. Furthermore, a minimum inhibitory effect was observed at high concentrations for yeasts, beginning at 70% for *S. cerevisiae* and at 90% for *C. albicans*. These data demonstrated the microbiostatic effect of Hrh-GTE. However, higher concentrations of this GTE were required for the microbicidal effects: 70% for *E. faecalis* and *S. enterica*, and 90% for *P. aeruginosa*, while the other studied microorganisms were not affected.

The reported antimicrobial effect of the sea buckthorn (Hrh) GTE was expected, given that the relevance of sea buckthorn has already been reported for other plant parts. Accordingly, the sea buckthorn pulp-related antimicrobial activity against *S. aureus*, *B. subtilis*, *S. typhimurium*, and *E. coli* has been reported [94]. In another study, the juice and by-product of sea buckthorn were effective against *Klebsiella pneumoniae*, *S. enterica*, *P. aeruginosa*, *Acinetobacter baumannii*, *Proteus mirabilis*, multidrug resistant *S. aureus*, *E. faecalis*, *E. faecium*, and *B. cereus* [95]. Chauhan et al. (2007) reported that aqueous extract of the seed showed antimicrobial effects against *L. monocytogenes* and *Yersinia enterocolitica* [96]. The leaf extract was effective against *B. subtilis* and *Bacillus thuringiensis*, while the seed extract exhibited significant antimicrobial activity against the former two bacteria, as well as against *Agrobacterium tumefaciens*, *Mucor indicus*, and *Tilletia indica* [97]. Moreover, the seed, pulp, and leaf extracted essential oils have shown antimicrobial activity against *B. subtilis*, *B. cereus*, *B. coagulans*, *S. aureus*, and *E. coli* [98]. Michel et al. (2012) found that the ethanolic extract of sea buckthorn leaf, stem, root, and seed were effective against *B. cereus*, *P. aeruginosa*, *E. coli*, *S. aureus*, *E. durans*, and *C. albicans* [99]. According to the literature, ethanolic extracts of leaf, stem, and seed showed antibacterial effects against *S. aureus*, *B. cereus*, and *E. durans* with inhibition percentages at 60–70% [99]. Other research has reported significant effects against *P. aeruginosa*, and *E. faecalis*, contributing this effect to the polyphenol content of sea buckthorn extracts [100]. Despite the antimicrobial effects of sea buckthorn mentioned earlier, few studies have explored features like the MIC and MMC. The sea buckthorn (Hrh) GTE has microbiostatic and microbicidal properties, though the underlying mechanisms remain unclear. Nonetheless, the sea buckthorn (Hrh) GTE features the highest condensed tannin content and the second most relevant antioxidant potential as assessed by TEAC and FRAP methods. According to our data it contains high concentration of catechin and carnosic acid, which can contribute to the antimicrobial activity [89,101].

#### 3.2.4. Blackthorn (Psp) and Common Grape (Vvi) GTEs Are Potent Antimicrobials That Exhibit Microbiostatic and Bactericidal Activities

Our study has proven that blackthorn (Psp) GTE, when assessed by the ADM, showed inhibition zones only for *S. aureus* and *B. cereus* starting at a high concentration of 70% (11.48 ± 0.91 mm and 9.84 ± 0.21 mm, respectively). The MIC and MMC assays revealed antimicrobial effects for many of the microbes studied. For Gram-Positive bacteria, a concentration of 40% is the minimum inhibitory concentration. For Gram-Negative bacteria, it is slightly higher (60–70%), except for *P. aeruginosa*, which showed effectiveness at 40%. The blackthorn (Psp) GTE exhibited inhibitory effects against yeasts at concentrations of 60% or higher. Higher concentrations were required for the fungicidal effects. For *L. monocytogenes*, the concentration was 50%; for *E. faecalis*, 70%; for *S. enterica*, 70%; and for *P. aeruginosa*, 80%, while for the remaining microorganisms studied the blackthorn (Psp) GTE had no effect.

Previous studies on blackthorn reported that the fruit extracts were effective against *E. coli*, *S. aureus*, *P. aeruginosa*, *E. faecalis*, *Salmonella enteritidis*, *Salmonella abony*, *Salmonella enterica ser. typhimurium*, *Enterobacter aerogenes* and *C. albicans* [102,103,104]. Dedić et al. (2021) found that ethanolic extracts of blackthorn fruit, leaf and flower had antimicrobial activity against *S. aureus*, *B. subtilis*, *E. faecalis*, *S. enterica*, *E. coli*, *P. aeruginosa* and *C. albicans* [105]. These data indicate that fruit and gemmotherapy extracts have similar antimicrobial activity.

During our studies, the common grape (Vvi) GTE showed limited antimicrobial effects through the ADM, being only effective against *B. cereus* at 100% concentration. Furthermore, it did not form any measurable inhibition zones for the analyzed yeast and mold species. For the MIC assay, we could conclude that the VVi-GTE proved somewhat more effective against Gram-Positive bacteria. At a 20% concentration, the common grape (Vvi) GTE was effective against *B. cereus*; at a 30% concentration, it was efficient against *S. aureus* and *L. monocytogenes*; and at a 50% concentration, it inhibited *E. faecalis*. At lower concentrations, the Vvi-GTE appeared to be less effective at inhibiting the growth of Gram-Negative bacteria. The 50% concentration proved effective against *P. aeruginosa*, while the 60% concentration was effective against *E. coli* and *S. enterica*.

The microbicidal effectiveness of the common grape (Vvi) GTE has been well confirmed in the MMC assay. In this assay, Vvi-GTE showed great potential, nearly equaling the effectiveness of the lingonberry (Vid) GTE. It was effective against all microorganisms studied. This effect is curious given that common grape (Vvi) GTE had one of the lowest measured antioxidant capacities, so the composition of its compounds warrants further analysis.

Previous studies have shown that the white grape juice was effective against several Gram-positive bacteria including *Moraxella catarrhalis*, *B. subtilis*, *Enterococcus durans*, *E. hirae*, *L. monocytogenes*, *S. aureus*, *S. epidermidis*, *S. mutans*, *S. pyogenes*, and *S. pneumoniae* and reduced the biofilm formation of *E. coli* and *P. aeruginosa* [106]. Grape pomace and seed contain high levels of condensed tannins, which can contribute to the antimicrobial effects. Merlot grape pomace extract inhibited the growth of *S. aureus* and *B. cereus*, while *E. coli*, *P. aeruginosa*, and *C. albicans* were more resistant [107]. Grape skin and seed extracts were effective against *S. aureus*, *K. pneumoniae*, *H. pylori*, *L. monocytogenes*, *E. faecium*, *E. faecalis*, and *Brochothrix thermosphacta* [51,108,109,110]. In another study the leaf extract of common grape inhibited the growth of *E. coli*, *P. aeruginosa*, *S. aureus*, and *E. faecalis* [111]. Xu et al. researched grape pomace for its antimicrobial effects against Gram-positive and negative bacteria strains and found significant antibacterial effect against *L. monocytogenes* and *Staphylococcus aureus*, and higher susceptibility against Gram-positive bacteria. Grape seed tannin extract showed 80% inhibition rate against *Plasmopara viticola* sporulation. The extract contained 4,55 mg/g catechin, and procyanidins between 0.5 and 1.8 mg/g [112].

According to the LC/MS measurement the blackthorn contained high concentration of chlorogenic acid, rutoside and caffeic acid, while the common grape contained moderate concentration of caffeic acid, quercetin, and rutoside. These components could contribute to the antimicrobial effects [89,113].

#### 3.2.5. Boxwood (Bsv), Hawthorn (Cox), and Raspberry (Rid) GTEs Exhibit Moderate Antimicrobial Activity

The boxwood (Bsv) GTE did not interfere with the ADM; therefore, no inhibition zones were detected for any of the microorganisms studied. However, one notable exception was *A. ochraceus*, which had a sporulation zone of 18.38 ± 1.01 mm and produced many spores. The MIC assay showed moderate effectiveness against Gram-positive bacteria. The 30% Bsv-GTE inhibited *B. cereus*; the 50% solution inhibited *S. aureus* and *L. monocytogenes*; and the 60% solution interfered with *E. faecalis*. Curiously, somewhat lower concentrations proved effective as a bacteriostatic against Gram-negative bacteria: 40% against *E. coli*, 50% against *P. aeruginosa*, and 70% against *S. enterica*. It also reduced the growth of *S. cerevisiae* to 60% and *C. albicans* to 70%. However, the bactericidal effects were negligible, only acting against *L. monocytogenes* at 60% and *P. aeruginosa* at 100%.

The boxwood derived extracts were shown by others to be effective against *Plasmodium falciparum* (tropical malaria) and *Trypanosoma brucei rhodesiense* (East African sleeping sickness) [114,115]. More studies had shown the boxwood efficient against *S. aureus*, *S. epidermidis*, *P. aeruginosa*, *Enterobacter* sp., *B. subtilis*, *B. cereus*, *Streptococcus* sp., *E. coli*, *E. faecalis*, *Proteus mirabilis*, *Proteus vulgaris*, *Serratia marcescens*, *Yersinia enterocolitica*, *K. pneumoniae*, *Salmonella adobraco*, *S. typhymurium*, *Campylobacter jejuni*, *Listeria ivanovii*, *Listeria innocua*, *L. monocytogenes*, *Corinebacterium xerosis*, *Rhodococcus equi*, *A. niger* and *C. albicans* [59,116].

Hawthorn (Cox) GTE was effective against most of the studied bacteria in the ADM, forming inhibition zones at some of the lowest concentrations: 20% for *S. aureus* (9.24 ± 0.13 mm), 50% for *B. cereus* (11.01 ± 0.4 mm), and 60% for *E. faecalis* (9.51 ± 0.17 mm). Unfortunately, there was no measurable effect on other types of microorganisms. The formation of a sporulation zone occurred in the case of *A. niger*, measuring 19.08 ± 0.48 mm, resulting in a significant number of spores. A similar outcome was observed in *A. ochraceus*, where a measurement of 16.67 ± 1.42 mm was recorded, also yielding a substantial spore count. The MIC assay produced mixed results. For the Gram-positive bacteria *B. cereus*, a concentration of 20% acts as the minimum inhibitory concentration, while 40% is required for *S. aureus*, 80% for *L. monocytogenes*, and 90% for *E. faecalis*. It proved to be somewhat more effective against Gram-negative bacteria, requiring lower concentration to be effective: at least 50% against *E. coli*, 60% against *P. aeruginosa*, and 70% against *S. enterica*. Interestingly, the hawthorn (Cox) GTE showed a minimum inhibitory effect at 80% concentrations against *C. albicans* and *S. cerevisiae*. Moreover, the hawthorn (Cox) GTE showed no microbicidal effects on any microorganisms studied.

Others studying the ethanolic hawthorn fruit extract were able to detect some antimicrobial effectiveness against *S. abony*, *E. coli*, *P. aeruginosa*, and *C. albicans* [117]. According to the literature, hawthorns are a rich source of condensed tannins, which can play a significant role in antimicrobial activities. Flavonoids extracted from the hawthorn fruit and fruit powder inhibited the growth of *S. aureus* and *E. coli* [86,118]. Another study reported that the essential oil extracted from hawthorn leaves was effective against *B. cereus*, *S. aureus*, *E. coli*, *P. aeruginosa*, and *S. typhi*. [119].

The raspberry (Rid) GTE studied by the ADM showed a limited antimicrobial effect, forming inhibition zones only at higher concentrations (70%) for *S. aureus*. It showed inhibitory effects against yeasts and molds, including *S. cerevisiae* and *A. niger*. In the case of *A. flavus*, it proved to be more effective, forming sporulation zones measuring 15.41 ± 2.12 mm and 11.56 ± 0.63 mm, resulting in the formation of many spores. Surprisingly, it had no visible effect on *A. ochraceus*. The MIC results looked more promising, with better bacteriostatic results for Gram-positive bacteria. The minimum inhibitory concentration is 20% for *B. cereus*, 30% for *S. aureus*, 60% for *L. monocytogenes*, and 80% for *E. faecalis*. Gram-negative bacteria proved to be slightly more resistant, requiring a minimum concentration of 60% to be effective. Furthermore, the raspberry (Rid) GTE exhibited minimum inhibitory effects against *S. cerevisiae* at concentrations of 90%, while it had no effect on *C. albicans*. The microbicidal effects of raspberry (Rid) GTE are much lower. High concentrations are required, and it is not effective against all microorganisms. It was efficient against *L. monocytogenes* at 70% and against *E. faecalis* and *S. enterica* at 100%.

Previous antimicrobial studies have shown that raspberry fruit extract is effective against *E. coli*, *S. typhimurium*, and *S. Enterica Serovar. infantis*, *P. aeruginosa*, *S. aureus*, *B. cereus*, *L. monocytogenes*, and *S. saprophyticus* [120,121]. Krauze-Baranowska et al. (2014) reported that the ethanolic extract of fruit inhibited the growth of *b-Haemolytic Streptococcus* groups A, B, and G, *Streptococcus pneumoniae* (clinical isolates), *Corynebacterium diphtheriae*, *E. faecalis*, *S. aureus*, *S. epidermidis*, *B. subtilis*, *Clostridium sporogenes*, *Klebsiella pneumoniae* (clinical isolate), *Neisseria meningitidis*, *Moraxella catarrhalis*, *Haemophilus influenzae*, and *H. pylori* [122]. Another study found that raspberry leaf extracts exhibit antimicrobial activity against certain bacterial triggers of autoimmune inflammatory diseases, including *Proteus mirabilis*, *Klebsiella pneumoniae*, *Acinetobacter baylyi*, *Pseudomonas aeruginosa*, and *Streptococcus pyogenes*. Remarkably, the combination of raspberry leaf extract and conventional antibiotics was more effective against *A. baylyi* than raspberry extract or antibiotics alone [123].

Therefore, the aforementioned research data and the antimicrobial properties of the raspberry (Rid) GTE suggest that combining them with certain antibiotics might enhance the effectiveness of traditional antibiotics.

Our results shows that raspberry contains high concentration of ellagic acid, while hawthorn contains chlorogenic acid, hyperoside and rutoside and these components can contribute to the mentioned antimicrobial activity [89,91,93].

### 3.3. A Comprehensive Antimicrobial Assessment Should Be Based on a Combination of Several Methods and Microbes

When studying the antimicrobial effects of GTEs, it is advisable to combine several microbial species and assessment methods to generate a larger dataset related to the topic under study. Based on our observations, we conclude that the broth microdilution method (BMM) with its MIC and MMC assays is more effective than the ADM. The bacterial and yeast strains were more susceptible to the GTEs in the MIC and MMC assays (see Table 4). This may be related to the limitations of the ADM. For instance, the size of the inhibition zone can be affected by the ambient temperature, humidity, and pH because these factors influence the diffusion rate of the tested GTE in agar. Other factors such as the molecular weight and solubility of phytonutrients can also cause uneven diffusion and distribution of antimicrobial agents. Furthermore, due to its reduced sensitivity, the ADM may miss low antimicrobial activity and requires larger amounts of the tested GTEs [124]. Moreover, the MIC indicates the bacteriostatic effect (visible growth inhibition of microorganisms) or the level of sensitivity or resistance of individual bacterial strains to the applied GTE. The MMC represents the lowest concentration of a given GTE that kills 99.9% of the tested microbial population. Therefore, the MMC distinctly indicates a microbicidal/bactericidal effect, which refers to the ultimate antimicrobial effectiveness of a given plant extract. The presented definitions of MIC and MMC substantiate the idea that bactericidal antimicrobials are superior to bacteriostatic ones [125]. Furthermore, the combination of bactericidal and bacteriostatic antibacterials was contraindicated in clinical practice due to antagonism and/or synergism type of interactions. In addition, certain bacteriostatic antibacterials are as effective as their bactericidal counterparts in clinical therapies. This raises the possibility of interactions between these antimicrobials that need to be evaluated in terms of their synergistic, antagonistic, or additive nature, enabling personalized treatment.

Our data have also revealed the hierarchy of sensitivity among the tested microorganisms, starting from the most to the least sensitive in the following order: (1) Gram-positive bacteria, (2) Gram-negative bacteria, (3) yeasts, and (4) molds. Given its structure, it seems logical to presume that the microbial cell membrane and wall act as a natural barrier. This initial point of contact may influence the movement of phytonutrients from the surrounding culture medium into microbial cells. The Gram-positive bacteria have a thick peptidoglycan layer, which, while porous, can still influence the penetration rate and concentration of phytonutrients across the cell wall reaching the cell membrane. Gram-negative bacteria possess a more complex cell wall with a thin peptidoglycan layer sandwiched between an inner and an outer membrane. The outer membrane, containing lipopolysaccharides (LPS), can act as a significant permeability barrier to various molecules, including some antioxidants [126]. Fungal cell walls are more complex, containing polysaccharides, glycoproteins, lipids, cellulose, chitin, and α- and β-glucans. These compounds protect cells against mechanical injuries and block the entry of different macromolecules [127]. Therefore, the susceptibility to the antimicrobial effects of phytonutrients could vary depending on differences in the structure of cell walls. Furthermore, it is also possible that certain phytonutrients bypassing the cell wall/membrane and reaching the interior of the microbial cell could interfere with intracellular pH, energy metabolism and DNA synthesis [128]. In this respect the polyphenols specific functional groups like the OH, alkyl, acetate and aldehydes can substantially influence their antibacterial properties.

The performed antimicrobial studies revealed a complex picture together with some specificities of a given GTE that are summarized in Table 4.

It was also interesting to observe that some of the tested microorganisms presented certain levels of susceptibility when were assessed for antibiotic resistance (see Figure 6). Even though the microorganisms were provided by a certified stock center and were considered normal, their antibiotic resistance was unknown. However, it is also possible that the previously acquired antibiotic resistance in some microbial strains could affect their susceptibility to further antibiotics (Table 4). Remarkably, the GTEs of lingonberry (Vid), dog rose (Rca), and sea buckthorn (Hrh) appeared to be the most efficient antimicrobials based on ADM (Table 4). This was true even in the case of Gram-positive bacterial strains with acquired antibiotic resistance, while the blackthorn (Psp), common grape (Vvi), raspberry (Rid), and boxwood (Bsv) GTEs would reveal mostly only MIC and/or MMC effects. No MMC properties were identified in the case of hawthorn (Cox) GTE. Surprisingly, the ADM was unable to detect any antimicrobial GTE activity among the tested Gram-negative bacteria. Conversely, the raspberry (Rid) GTE increased the viability of *S. cerevisiae* (Table 4). Previous studies have indicated that phytonutrients such as oleic acid, linolenic acid [129], phenylalanine, glutamic acid, serin, threonine [130], biotin [131,132], calcium pantothenate, pyridoxine, and thiamine [133] would facilitate the growth of *C. albicans* and *S. cerevisiae.* Since raspberry (Rid) GTE contains some of the mentioned amino acids, we hypothesize that these compounds could have led to growth-promoting effects (un-published results). Furthermore, and remarkably, some GTEs exhibited antifungal effects. These included dog rose (Rca), blackthorn (Psp), hawthorn (Cox), and raspberry (Rid), which produced inhibition zones, as indicated by the ADM.

Taken together, the ADM, MIC, and MMC data, as well as the antibiotic susceptibility of the tested microbial strains, reveal the complexity and versatility of GTEs in combating microbial infections. Integrating all these aspects poses unprecedented challenges to our current understanding.

### 3.4. The Antimicrobial Properties of GTEs Cannot Be Explained Solely by Their Total Polyphenol Content

Several studies have suggested the antimicrobial relevance of plant-derived polyphenols, invoking possible interactions between polyphenols and bacterial physiology [128] or cell surfaces.

Comparing the GTE polyphenol data to the specific antimicrobial effects on different microorganisms we could conclude that dog rose (Rca), sea buckthorn (Hrh), and lingonberry (Vid) GTEs were particularly effective against Gram-positive types *S. aureus*, *B. cereus*, and *E. faecalis*, and somewhat effective against *L. monocytogenes* (see Table 2), suggesting a putative correlation between the given GTEs specific polyphenol content and the antimicrobial effect on ADM. For the MIC studies, almost the same could be found, where for the Gram-positive bacteria the dog rose (Rca) extract was effective even at 10% for *B. cereus*, 20% for *S. aureus* and *E. faecalis*, and at 30% for *L. monocytogenes*. Lingonberry (Vid) GTE has also shown promising results, being effective even at 10% for *B. cereus*, 20–30% for *S. aureus* and *E. faecalis*, and at 40% for *L. monocytogenes*. Similarly, the sea buckthorn (Hrh) GTE has relatively high polyphenol content and showed relevant antimicrobial activity on MIC assays (Table 4). The blackthorn (Psp), common grape (Vvi), hawthorn (Cox), Raspberry (Rid), and boxwood (Bsv) were displaying variable MIC data across tested bacteria and yeast species, while their total polyphenol content was also variable but substantially reduced as compared to the most efficient dog rose (Rca), sea buckthorn (Hrh), and lingonberry (Vid) GTEs.

A similar distribution pattern could be seen for the total flavonoid content, as well as the total polyphenol content, among the tested GTEs, indicating no striking difference that could be invoked for antimicrobial specificity.

However, among polyphenols, the tannins with their numerous free hydroxyl groups are one of the most significant subgroups regarding antimicrobial activities [134]. Tannins can inhibit both Gram-positive and Gram-negative bacterial growth, but most tannins have shown a bacteriostatic effect rather than a bactericidal activity. The antibacterial effects of tannin-rich extracts vary depending on the specific type of tannins they contain. For instance, mimosa extract, which is abundant in condensed tannins (CT), exhibited bacteriostatic properties, whereas chestnut extract, rich in hydrolysable tannins (HT), demonstrated bactericidal activity [135]. Our experimental data indicate that lingonberry (Vid), dog rose (Rca), and sea buckthorn (Hrh) GTEs have a high polyphenol content based on their TPC, TFC, and CTC values (see Figure 1). These values also suggest that condensed tannins may play a key role in the bacteriostatic antimicrobial effects of the corresponding GTEs (see Table 4). However, we observed that the common grape (Vvi) GTE had a strong microbicidal effect, which was not expected based on its condensed tannin content (Table 4). Similarly, the specific condensed tannin content of sea buckthorn (Hrh) GTE is more relevant; however, it exhibits an important bacteriostatic property in addition to the expected bactericidal effect (Table 4). Another striking discrepancy would refer to the raspberry (Rid), blackthorn (Psp), and boxwood (Bsv) GTEs that do not contain condensed tannins, yet they all have bacteriostatic properties.

Furthermore, there is no direct correlation between the antioxidant activity of the GTEs and the ADM for Gram-negative bacteria, yeasts, or molds since none of the GTEs exhibited antimicrobial properties for the above-mentioned bacterial category. For the MIC essay, we obtained different results, where the highest concentration of condensed tannin content in GTEs produced the best results, as sea buckthorn (Hrh) GTE was effective starting at 40% concentration for *E. coli*. Dog rose (Rca) and lingonberry (Vid) GTEs were only effective at concentrations of 50% or above. However, irrespective of their polyphenol content, all GTEs showed some bacteriostatic effects against yeast, apart from raspberry (Rid) GTE that specifically increases viability of *S. cerevisiae*.

Astonishingly, it also turned out that the GTEs of raspberry (Rid), blackthorn (Psp), common grape (Vvi), and boxwood (Bsv) have significantly lower polyphenol content than the most efficient antibacterial GTEs. However, they still exhibit MIC and MMC properties.

Interestingly, based on our research data no obvious correlation could be established between the polyphenol content of GTEs and the specificity of their antimicrobial activity. The methodology used to assess the antimicrobial effect (ADM, MIC, and MMC) seems relevant. In addition to the current methods, time–kill curves should be considered, as they can distinguish between the bactericidal and bacteriostatic properties of the tested antimicrobial by assessing the speed and extent of antimicrobial activity. There is future for GTEs but further, in-detail antimicrobial studies are needed to fully determine their true value and effectiveness.

## 4. Materials and Methods

### 4.1. Sample Collection

The presented research is based on eight different GTEs corresponding to species such as *Rubus idaeus* L., *Hippophae rhamnoides* L., *Prunus spinosa* L., *Rosa canina* L., *Vaccinium vitis-idaea* L., *Crataegus oxyacantha* L., *Buxus sempervirens* L., *Vitis vinifera* L. Buds and young shoots were collected at various times in Cluj County, Romania. Raspberry, blackthorn, and lingonberry were collected from the Marișel area (46°41′7″ N 23°6′31″ E), dog rose and hawthorn from the Baciu area (46°47′34″ N 23°31′30″ E), sea buckthorn and common grape from the PlantExtrakt company’s own plantation in the Cristorel area (46°57′53″ N 23°27′47″ E), while the boxwood also from the company’s own plantation, but from the Baciu area. Blackthorn buds were collected in March 2022, boxwood in June 2022, sea buckthorn in March 2023, common grape in April 2023, raspberry, dog rose, hawthorn and lingonberry in June 2023. The fresh plant material was processed within a maximum of 6 h after collection or stored in a refrigerator at 4 °C for up to 24 h until processing.

### 4.2. Sample Preparation and Extraction

The extraction method follows the procedure outlined in the European Pharmacopoeia [45]. The extracts were prepared from freshly harvested plant materials, which were preserved in a mixture of 96% (*v*/*v*) ethanol and glycerol (1:1). The solvent amount was calculated to achieve a dry plant-to-solvent ratio of 1:20. The plant-solvent mixture was periodically mixed for 20 days, 2 × 20 min per day. In the next step, the solid and liquid components of the mixture were separated, and the extracted solid plant material was further pressed to increase the extraction yield at a maximum of 400 atm. The two extracted solutions (separated from the solid part and those obtained after pressing the solid part) were combined to form the concentrated extracts, which were then used in further studies. The extracts were stored in a refrigerator at 4 °C until use. The concentrated extract was defined as 100% in the antimicrobial assays, corresponding to a concentration of 50 mg/mL, based on a dry plant-to-solvent ratio of 1:20.

For the antimicrobial analysis, ethanol was removed from the extracts using a rotavapor, operated at a maximum temperature of 40 °C and 200 mbar pressure, to prevent the degradation and loss of bioactive compounds. The evaporated ethanol was immediately replaced with purified water. The resulting samples were stored in a refrigerator at 4 °C until the study was conducted.

### 4.3. Determination of Total Phenolic Content (TPC)

Total phenolic content was determined with the Folin-Ciocâlteu method according to Singleton and Rossi (1965) [136] with minor modifications [137]. A total of 1 mL of GTE was diluted to 10 mL with methanolic distilled water (4:1) and centrifuged at 13,400 rpm for 10 min, using an Eppendorf 5452 MiniSpin centrifuge (Eppendorf SE, Hamburg, Germany). Then, 0.5 mL of each filtrated sample was pipetted into test tubes and 2.5 mL of 0.2 N Folin reagent (VWR International, Leuven, Belgium) was added to each tube, then left to stand for 5 min. A total of 2 mL of 75 g/L Na_2_CO_3_ (VWR International, Leuven, Belgium) was added, and all samples were left to rest in a dark cabinet for 2 h. The blank was made in the same way, except that methanolic distilled water was used instead of the sample. Samples were measured at 760 nm using a spectrophotometer (Ultrospec 2100 pro, Biochrom Ltd., Cambridge, England). To zero the instrument, methanolic distilled water (4:1) was used. 100 mg/L gallic-acid solution (Thermo Fisher, Kandel, Germany) was used as a standard and 5, 10, 20, 50, 100 mg/L calibration solutions were prepared, and the reagents were mixed as described above. The results were expressed in milligrams of gallic acid equivalent (GAE) per mL.

### 4.4. Determination of Total Flavonoid Content (TFC)

The total flavonoid content was measured by aluminum chloride colorimetric method [138] with minor modifications [137]. Sample preparation was the same as described in Section 4.3. When the samples were prepared, 4 mL of pure distilled water were pipetted into the test tubes and 1 mL of each filtered sample was added. Then, 0.3 mL of 5% NaNO_2_ (VWR International, Leuven, Belgium) solution was added. After 5 min 0.3 mL of 10% AlCl_3_ (VWR International, Leuven, Belgium) solution was pipetted and stand for 1 min. After that, 2 mL of 1 M NaOH (VWR International, Leuven, Belgium) and 2.4 mL of distilled water were added. The blank was prepared in the same way, except that methanolic distilled water was used instead of the sample. The samples were measured at 510 nm using a spectrophotometer. Methanolic distilled water (4:1) was used to zero the instrument. A total of 100 mg/L catechin solution (Merck, Darmstadt, Germany) was used as standard for the calibration curve and 20, 40, 60, 80, 100 mg/L calibration solutions were prepared, and the reagents were mixed as described above. Results were expressed as milligram of catechin equivalent (CE) per mL.

### 4.5. Determination of Condensed Tannin Content (CTC)

The condensed tannin content was measured by the vanillin-HCl assay of Price et al. (1978) [139] with minor modifications by Hagerman (2002) [74]. A total of 1 mL of extract was pipetted into the test tubes and 5 mL of working vanillin was added (1% vanillin (Thermo Fisher, Kandel, Germany) in methanol (VWR International, Rosny-sous-Bois, France) mixed with 8% concentrated HCl (VWR International, Fontenay-sous-Bois-cedex, France) in methanol, 1:1). Blank samples were prepared in a similar manner for each sample, except that 4% concentrated HCl in methanol was added instead of working vanillin. The samples were then incubated in 30 °C for 20 min. The samples were measured at 500 nm using a spectrophotometer. Methanol was used to zero the instrument. A total of 0.3 mg/mL catechin solution was used as standard for the calibration curve and 0.06, 0.12, 0.18, 0.24, 0.3 mg/L calibration solutions were prepared, and the reagents were mixed as described above. Results were expressed as milligram of catechin equivalent (CE) per mL.

### 4.6. Determination of Antioxidant Activity Using DPPH Assay

The antioxidant activity was measured using 2,2-diphenyl-1-picrylhydrazyl (DPPH) free radicals (Merck, Darmstadt, Germany) [140]. A total of 1 mL of each GTE was diluted to 10 mL with methanol and left for 20 min with periodic vortexing. They were then centrifuged at 13,400 rpm for 10 min. A 90 mg/L methanolic DPPH solution was prepared and after that 100 μL sample was pipetted into the test tubes. Then, 1400 μL methanol and 1500 μL DPPH solutions were added. The blank wad was made using 1500 μL methanol and 1500 μL DPPH solutions. The mixtures were incubated in the dark for 30 min, and the absorbance was measured at 517 nm using a spectrophotometer. Methanol was used to zero the instrument. A 1 mg/mL methanolic Trolox solution (Thermo Fisher Scientific, Waltham, MA, USA) was used as standard and 0.33, 0.125, 0.1, 0.05, 0.033 mg/mL calibration solutions were prepared, and the reagents were mixed as described above. The results were expressed as milligram of Trolox equivalent (TE) per mL.

### 4.7. Determination of Antioxidant Activity Using TEAC Assay

The antioxidant activity was measured using 2,2′-azino-bis(3-ethylbenzothiazoline-6-sulfonic acid) (ABTS) (Merck, Darmstadt, Germany) stable radicals [141]. Free radicals were made by mixing 4.95 mmol/L potassium persulfate (VWR International, Leuven, Belgium) with 7 mmol/L ABTS in 1:1 ratio keeping it in dark for 12 h. After that, a ten-fold dilution was made with distilled water, and pH was set to 7.7 with HCl. 1 mL of each GTE was diluted to 10 mL with methanol and left for 20 min with periodic vortexing. They were then centrifuged at 13,400 rpm for 10 min. A total of 100 μL sample, 1000 μL distilled water, and 900 μL ABTS free radical solution were mixed. The blank was made using 1000 μL distilled water and 1000 μL ABTS solutions. The mixtures were incubated in the dark for 20 min and the absorbance was measured at 734 nm using a spectrophotometer. Methanol was used to zero the instrument. A 1 mg/mL methanolic Trolox solution was used as standard and 0.2, 0.125, 0.1, 0.05, 0.033 mg/mL calibration solutions were prepared, and the reagents were mixed as described above. The results were expressed as milligrams of Trolox equivalent (TE) per mL.

### 4.8. Ferric Reducing Antioxidant Power (FRAP) Assay

The antioxidant power was measured by FRAP assay [142]. Sample preparation was the same as described in Section 4.6. except that distilled water was used for dilution instead of methanol. The FRAP solution was prepared as follows: A total of 25 mL acetate buffer + 2.5 mL TPTZ solution (2,4,6-tri(2-pyridinyl)-1,3,5-triazine) + 2.5 mL ferric chloride solution. Acetate buffer: 3.1 g sodium-acetate · 3H2O (VWR International, Leuven, Belgium) + 16 mL acetic acid (VWR International, Leuven, Belgium) + 1 L distilled water; ferric chloride solution: 54 mg FeCl_3_ (VWR International, Leuven, Belgium) + 10 mL distilled water; TPTZ solution: 31.23 mg TPTZ (Thermo Fisher Scientific, Waltham, MA, USA) + 10 mL distilled water + 33.5 μL hydrochloric acid (37 *v*/*v*%). When the FRAP is prepared 65 μL distilled water was added to the test tubes. After that, 10 μL samples were added followed by 2250 μL FRAP solvent. The blank was made using 75 μL distilled water and 2250 μL FRAP solution. The mixtures were incubated for 5 min, and the absorbance was measured at 593 nm using a spectrophotometer. Distilled water was used to zero the instrument. A total of 1 mg/mL ascorbic acid solution (VWR International, Leuven, Belgium) was used as standard and 1, 0.5, 0.2, 0.125, 0.1, 0.05, 0.033 mg/mL calibration solutions were prepared, and the reagents were mixed as described above. The results were expressed as milligram of ascorbic acid equivalent (AAE) per mL.

### 4.9. LC/MS Determination of Polyphenols Profile

The LC/MS method was performed on a Shimadzu Nexera I LC/MS—8045 (Kyoto, Japan) UHPLC system equipped with a quaternary pump and autosampler, respectively, an ESI probe and quadrupole rod mass spectrometer. The separation was carried out on a Luna C18 reversed phase column (150 mm × 4.6 mm × 3 mm, 100 Å), from Phenomenex (Torrance, CA, USA). The column was maintained at 40 C degrees during the analyses. The mobile phase (see Table 5) was a gradient made from methanol (Merck, Darmstadt, Germany) and ultrapurified water prepared by Simplicity Ultra Pure Water Purification System (Merck Millipore, Billerica, MA, USA). As an organic modifier was used formic acid (Merck, Darmstadt, Germany). The methanol and the formic acid were of LC/MS grade. The used flow rate was of 0.5 mL/min. The total time of an analyses is 35 min.

The detection was performed on a quadrupole rod mass spectrometer operated with electrospray ionization (ESI), both in negative and positive MRM (multiple reaction monitoring) ion mode (see Table 6). The interface temperature was set at 300 °C degrees. For vaporization and as drying gas nitrogen was used at 35 psi, respectively, at 10 L/min. The capillary potential was set at +3000 V.

They were used as standards the substances from Table 6 and Table 7, purchased from Phytolab, Vestenbergsgreuth, Germany. From each standard, each concentration was injected with 1 mL. The identification was performed by comparison of MS spectra and its transitions between the separated compounds and standards. The identification and quantification are made based on the main transition from the MS spectra of the substance. For quantification purposes, the calibration curves were determined. In Table 7, the calibration curves equations can also be observed, including their correlation factors and the determined limit of detection and quantification. In Table 6, the retention times and the specific MS spectra data for the used standards are also presented.

### 4.10. Studied Microorganisms

The bacterial and fungal strains used in this study were obtained from the National Collection of Agricultural and Industrial Microorganisms (NCAIM, Budapest, Hungary). The antimicrobial activity of different GTEs was assessed on seven bacterial strains: four Gram-positive bacteria (*Staphylococcus aureus* B.01055, *Enterococcus faecalis* B.01054, *Bacillus cereus* B.00076, *Listeria monocytogenes* wild strain) and three Gram-negative bacteria (*Pseudomonas aeruginosa* B.01064, *Escherichia coli* B.00200, *Salmonella enterica* subsp. *enterica* B.00834). Additionally, five mycotoxigenic fungi were tested: *Penicillium citrinum* F.00815, *Penicillium expansum* F.00601, *Aspergillus flavus* F.00048, *A. ochraceus* F.00850, *A. niger* F.00071, and two yeast strains, *Candida albicans* wild strain and *Saccharomyces cerevisiae* Y.00481.

Bacterial strains were cultivated on nutrient agar (distilled water 1000 mL, peptone 10 g, meat extract 10 g, NaCl 5 g, agar 18 g,) at 37 °C for 24 h, while molds and yeasts were cultivated on a complex medium (distilled water 1000 mL, peptone 10 g, yeast extract 10 g, glucose 40 g, agar 20 g) at 28 °C for 72 h. All media materials were obtained from VWR International L.L.C. (Debrecen, Hungary).

### 4.11. Antimicrobial Activity Assessment

The antimicrobial activity to GTEs was investigated by agar-well diffusion method, based on Heatley (1944) [143].

A series of GTE concentrations ranging from 0 to 100% (*v*/*v*) was prepared, with 100% representing the concentrated GTE. The other concentrations were achieved by diluting the concentrated GTE with sterile distilled water.

A bacterial and fungal suspension with an optical density (OD) of 1 was prepared in a turbidity tube using physiological saline solution (NaCl 0.9%). For OD adjustment, a Biolog Turbidimeter 21907 (Biolog Inc., Hayward, CA, USA) was used. From these tubes, 0.1 mL of microbial suspension (~10^8^ CFU/mL) was inoculated onto the surface of the medium (nutrient medium for bacteria, complex medium for fungal species). An 8 mm diameter hole was then cut in the center of the medium, into which 0.1 mL of extracts at various concentrations were pipetted. After incubation (24 h at 37 °C for bacterial strains and 48 h at 28 °C for fungal strains), the diameter of the inhibition zones (including the hole) was measured using a digital caliper [144]. To ensure accuracy, the average of three parallel measurements was calculated.

In general, the tested extract is more effective against a given microorganism, the larger the zone of inhibition.

### 4.12. Antimicrobial Susceptibility Test Assay

For antimicrobial susceptibility test assay three antibiotics were used: tetracycline 30 mcg, kanamycin 30 mcg, and cefotaxime 30 mcg. All antibiotics were purchased from HiMedia Laboratories GmbH (Modautal, Germany).

All experiments were carried out according to Section 4.11., except that instead of cutting holes in the medium, antibiotic disks (6 mm) were placed in the middle of the media. This assay was used only on bacterial strains.

### 4.13. Broth Microdilution Method (BMM)

The assay was conducted using 96-well plates. Each concentrated GTE was serially diluted in the wells of the microplate to obtain a 100-μL mixed solution, using nutrient/complex broth as the dilution medium. The result was a concentration range between 0% and 100%. Each concentration was made in triplicate.

### 4.14. Minimum Inhibitory and Minimum Microbicidal Concentration Assay (MIC and MMC)

The MICs were determined as in our previous article [5], using a method described by Agbebi et al. (2022) [145] and El Baaboua et al. (2022) [146]. In some cases, the color of the extract made it impossible to obtain an accurate result. Therefore, MIC values were determined using both the cell count method and the resazurin method.

### 4.15. Statistical Analysis

All analyses were conducted in triplicate, and the results were expressed as the mean ± standard deviation (SD). Statistical analysis was performed using IBM SPSS Statistics 26. For antimicrobial activity, the data were analyzed using one-way analysis of variance (ANOVA) followed by Tukey’s HSD test to determine significant differences within various concentrations. The difference was considered significant when *p* < 0.05.

## 5. Conclusions

The GTEs are novel remedies used in phytotherapy. The comparative nature of the current study allows for a direct valuation between the GTEs obtained from different plant species. This kind of assessment would comprise features like TPC, TFC, CTC, TEAC, DPPH, FRAP, and antimicrobial properties based on ADM and BMM to determine MIC and MMC.

In general, the lingonberry (Vid), dog rose (Rca), sea buckthorn (Hrh), and hawthorn (Cox) GTEs have high TPC, TFC, and CTC values (see Figure 1), in that order. Following them are the much-reduced TPC and TFC featuring raspberry (Rid), blackthorn (Psp), common grape (Vvi), and boxwood (Bsv) GTEs. The CTC is greatly reduced in the common grape (Vvi), and no condensed tannin was detected in the raspberry (Rid), blackthorn (Psp), or boxwood (Bsv). The in vitro antioxidant capacity, as measured by TEAC, DPPH, and FRAP, appears to be highly correlated with TPC. Dog rose (Rca), sea buckthorn (Hrh), lingonberry (Vid), and raspberry (Rid) GTEs have the highest values, while the others have much lower values (see Figure 2).

The Psp-GTE sample contained the highest levels of chlorogenic acid, rutoside, and caffeic acid. The Rid-GTE and Vid-GTE samples stood out for their high ellagic acid content, while Cox-GTE showed a favorable phenolic profile mainly due to its chlorogenic acid and hyperoside content.

Regarding the antimicrobial assessment using the ADM, the studied GTEs revealed a clear specificity for Gram-positive bacteria, while no inhibitory effects were detected for Gram-negative bacteria (see Table 2 and Table 4). However, when the BMM was applied, MIC effects were mostly observed for both Gram-positive and Gram-negative bacteria, as well as the studied yeast species. Meanwhile, the lingonberry (Rid), common grape (Vvi), blackthorn (Psp), and raspberry (Rid) GTEs displayed variable MMC-based bactericidal effects. Our results demonstrated that the ADM is less efficient than the BMM since both MIC and MMC assays it inhibited growth or had otherwise microbicidal effects even at lower concentrations against the microorganisms studied. Compared to widely used antibiotics, the GTEs studied above have shown their antimicrobial potential, which warrants further, especially clinical, studies regarding their use in fighting antimicrobial resistance.

Results: The analyzed dog rose, lingonberry, sea buckthorn, blackthorn, and common grape GTEs exhibited the highest total phenolic content and antioxidant activity. They also demonstrated notable antimicrobial activity, including variations in microbiostatic and/or microbicidal properties. These results demonstrate the relative strength of the antimicrobial effects of specific GTEs against certain microbial species, which could facilitate the use of certain GTEs in personalized and/or specific antimicrobial therapies.

## Figures and Tables

**Table 1:** Quantitative assessment of the selected phytochemicals among the studied GTEs determined by the LC/MS method.

Identified Compounds	Concentration, mg/mL							
	Rid-GTE	Psp-GTE	Hrh-GTE	Rca-GTE	Vid-GTE	Cox-GTE	Bsv-GTE	Vvi-GTE
Caffeic acid	0.118 ± 0.0052	1.759 ± 0.0952	0.814 ± 0.0207	-	0.831 ± 0.0541	0.813 ± 0.0247	-	0.813 ± 0.0207
Carnosic acid	-	-	1.009 ± 0.0875	-	-	-	-	-
Chlorogenic acid	0.135 ± 0.0087	7.546 ± 0.1355	0.150 ± 0.0085	0.476 ± 0.0243	1.680 ± 0.0821	3.865 ± 0.1749	-	0.151 ± 0.0082
*trans-p*-coumaric acid	-	0.712 ± 0.0407	-	-	-	-	-	-
Ellagic acid	2.246 ± 0.1021	-	-	-	2.084 ± 0.1512	-	-	-
Ferulic acid	-	0.159 ± 0.0098	-	-	-	-	-	-
Gallic acid	0.056 ± 0.0074	-	0.092 ± 0.0078	0.057 ± 0.0041	-	-	-	0.058 ± 0.0012
Salicylic acid	0.507 ± 0.0421	0.161 ± 0.0091	-	0.261 ± 0.0132	0.171 ± 0.0089	-	-	0.090 ± 0.0021
Apigenin	0.018 ± 0.0010	0.016 ± 0.0003	0.034 ± 0.0012	0.027 ± 0.0002	0.017 ± 0.0008	0.008 ± 0.0001	0.019 ± 0.0001	0.027 ± 0.0015
Catechin	-	0.220 ± 0.0109	5.849 ± 0.2865	0.443 ± 0.0288	0.301 ± 0.0221	-	-	0.030 ± 0.0019
Chrysin	0.116 ± 0.0143	0.089 ± 0.0042	0.176 ± 0.0183	0.115 ± 0.0089	0.111 ± 0.0056	0.121 ± 0.0102	0.118 ± 0.0052	0.102 ± 0.0081
Hyperoside	0.220 ± 0.0193	0.837 ± 0.0654	0.151 ± 0.0101	0.785 ± 0.0365	1.446 ± 0.1073	1.822 ± 0.0831	0.178 ± 0.0073	0.199 ± 0.0088
Kaempferol	0.029 ± 0.0017	0.319 ± 0.0187	-	-	0.026 ± 0.0014	-	-	0.041 ± 0.0030
Luteolin	-	0.007 ± 0.0001	0.011 ± 0.0009	-	0.019 ± 0.0007	-	-	0.013 ± 0.0010
Luteolin-7-*O*-glucosid	0.074 ± 0.0041	0.073 ± 0.0019	0.090 ± 0.0062	0.076 ± 0.0018	0.086 ± 0.0024	-	-	0.301 ± 0.0211
Naringenin	0.035 ± 0.0024	0.060 ± 0.0011	0.061 ± 0.0053	0.020 ± 0.0008	0.024 ± 0.0008	0.131 ± 0.0351	0.026 ± 0.0008	0.027 ± 0.0009
Quercetin	0.107 ± 0.0084	0.953 ± 0.0642	0.038 ± 0.0021	0.036 ± 0.0013	-	0.071 ± 0.0009	-	0.413 ± 0.0309
Quercitrin	-	0.305 ± 0.0187	-	0.248 ± 0.0167	-	-	-	-
Rutoside	0.159 ± 0.0088	6.385 ± 0.2431	0.228 ± 0.0184	0.347 ± 0.0203	1.210 ± 0.0845	0.902 ± 0.0411	0.608 ± 0.0125	0.378 ± 0.0238
Vitexin	-	0.033 ± 0.0010	-	-	-	0.056 ± 0.0016	-	0.033 ± 0.0011
Carnosol	0.027 ± 0.0009	0.029 ± 0.0022	-	-	0.026 ± 0.0010	-	-	0.026 ± 0.0020
Arbutoside	-	-	-	-	1.330 ± 0.0852	-	-	-

Note: Rid—raspberry, Psp—blackthorn, Hrh—sea buckthorn, Rca—dog rose, Vid—lingonberry, Cox—hawthorn, Bsv—boxwood, Vvi—common grape, GTE—gemmotherapy extract.

**Table 2:** The antimicrobial activity of GTEs against Gram-positive bacteria as revealed by the ADM (concentrations and inhibition zones).

Bacteria	Conc. %	Rid-GTE	Psp-GTE	Hrh-GTE	Rca-GTE	Vid-GTE	Cox-GTE	Bsv-GTE	Vvi-GTE
*S. aureus*	100	12.15 ± 0.64 ^a^	12.53 ± 1.5 ^a,b^	16.31 ± 1.64 ^a^	14.75 ± 0.42 ^a^	13.96 ± 0.37 ^a^	12.16 ± 0.29 ^a^	nd	nd
	90	11.33 ± 1.03 ^b,c^	12.95 ± 1.21 ^a,d^	14.85 ± 1.46 ^a,b^	14.46 ± 0.27 ^a^	13.43 ± 0.31 ^b^	12.09 ± 0.34 ^a,b^	nd	nd
	80	11.73 ± 1.09 ^a,b^	12.21 ± 0.39 ^b,c^	13.94 ± 2.49 ^b,c^	14.59 ± 0.26 ^a^	13.35 ± 0.32 ^b,c^	12.15 ± 0.31 ^a,b^	nd	nd
	70	11.04 ± 0.47 ^c^	11.48 ± 0.91 ^c,d^	12.92 ± 1.53 ^c,d^	13.51 ± 0.19 ^b^	13.33 ± 0.26 ^b,c^	11.86 ± 0.27 ^b^	nd	nd
	60	nd	nd	11.63 ± 0.64 ^d,e^	13.78 ± 0.52 ^b^	13.04 ± 0.28 ^c^	11.41 ± 0.21 ^c^	nd	nd
	50	nd	nd	10.95 ± 1.71 ^e^	12.84 ± 0.24 ^c^	11.62 ± 0.29 ^d^	11.47 ± 0.22 ^c^	nd	nd
	40	nd	nd	10.06 ± 0.6 ^e^	12.17 ± 0.3 ^d^	12.26 ± 0.23 ^e^	10.64 ± 0.14 ^d^	nd	nd
	30	nd	nd	nd	11.52 ± 0.19 ^e^	11.34 ± 0.29 ^d^	10.38 ± 0.28 ^d^	nd	nd
	20	nd	nd	nd	10.85 ± 0.27 ^f^	10.92 ± 0.15 ^f^	9.24 ± 0.13 ^e^	nd	nd
	10	nd	nd	nd	nd	9.85 ± 0.24 ^f^	nd	nd	nd
*B. cereus*	100	nd	9.88 ± 0.46 ^a,b^	12.44 ± 0.44 ^a^	16.32 ± 0.20 ^a^	15.46 ± 0.28 ^a^	13.14 ± 0.17 ^a^	nd	9.85 ± 0.26
	90	nd	10.09 ± 0.43 ^a^	13.39 ± 1.03 ^b^	14.62 ± 0.27 ^b^	14.46 ± 0.28 ^b^	12.02 ± 0.15 ^b^	nd	nd
	80	nd	9.74 ± 0.26 ^b^	11.65 ± 0.39 ^c^	13.69 ± 0.21 ^c^	14.82 ± 0.53 ^b^	12.37 ± 0.29 ^c^	nd	nd
	70	nd	9.84 ± 0.21 ^a,b^	11.85 ± 0.68 ^a,c^	13.78 ± 0.42 ^c^	13.24 ± 0.21 ^c^	11.48 ± 0.16 ^d^	nd	nd
	60	nd	nd	10.91 ± 0.69 ^d^	13.35 ± 0.26 ^d^	13.23 ± 0.45 ^c^	11.12 ± 0.18 ^e^	nd	nd
	50	nd	nd	10.84 ± 0.97 ^d^	12.78 ± 0.26 ^e^	12.62 ± 0.23 ^d^	11.01 ± 0.40 ^e^	nd	nd
	40	nd	nd	10.71 ± 0.13 ^d^	13.05 ± 0.19 ^d,e^	12.38 ± 0.20 ^d^	nd	nd	nd
	30	nd	nd	nd	12.01 ± 0.22 ^f^	11.61 ± 0.30 ^e^	nd	nd	nd
	20	nd	nd	nd	11.78 ± 0.36 ^f^	nd	nd	nd	nd
	10	nd	nd	nd	9.69 ± 0.13 ^g^	nd	nd	nd	nd
*E. faecalis*	100	nd	nd	12.27 ± 0.58 ^a^	11.84 ± 0.23 ^a^	12.54 ± 0.16 ^a^	10.31 ± 0.25 ^a^	nd	nd
	90	nd	nd	11.39 ± 0.27 ^b^	11.39 ± 0.34 ^b^	11.61 ± 0.13 ^b^	9.87 ± 0.15 ^b^	nd	nd
	80	nd	nd	10.31 ± 0.15 ^c^	11.15 ± 0.25 ^b,c^	11.14 ± 0.19 ^c^	9.79 ± 0.15 ^b^	nd	nd
	70	nd	nd	10.16 ± 0.35 ^c^	10.86 ± 0.15 ^c^	10.9 ± 0.18 ^c,d^	9.7 ± 0.16 ^b,c^	nd	nd
	60	nd	nd	9.61 ± 0.26 ^d^	10.16 ± 0.19 ^d^	10.57 ± 0.18 ^e,f^	9.51 ± 0.17 ^c^	nd	nd
	50	nd	nd	9.16 ± 0.19 ^e^	10.51 ± 0.23 ^d^	10.91 ± 0.39 ^c,d^	nd	nd	nd
	40	nd	nd	nd	nd	10.67 ± 0.24 ^d,e^	nd	nd	nd
	30	nd	nd	nd	nd	10.33 ± 0.13 ^f^	nd	nd	nd
	20	nd	nd	nd	nd	nd	nd	nd	nd
*L. monocytogenes*	100	nd	nd	10.34 ± 0.38 ^f^	* 18.86 ± 0.44 ^a,b,c^	12.82 ± 0.38 ^a^	nd	nd	nd
	90	nd	nd	nd	* 18.95 ± 0.56 ^a,b,c^	11.93 ± 0.23 ^b^	nd	nd	nd
	80	nd	nd	nd	* 18.72 ± 0.52 ^b,c^	11.46 ± 0.17 ^c^	nd	nd	nd
	70	nd	nd	nd	* 19.27 ± 0.32 ^a,b^	11.51 ± 0.12 ^c^	nd	nd	nd
	60	nd	nd	nd	* 19.16 ± 0.19 ^a,b^	10.2 ± 0.15 ^d^	nd	nd	nd
	50	nd	nd	nd	* 19.46 ± 0.37 ^a,b^	10.01 ± 0.17 ^d^	nd	nd	nd
	40	nd	nd	nd	* 18.5 ± 0.22 ^c,d^	9.48 ± 0.23 ^e^	nd	nd	nd
	30	nd	nd	nd	* 18.08 ± 0.44 ^d^	nd	nd	nd	nd
	20	nd	nd	nd	* 17.04 ± 0.34 ^e^	nd	nd	nd	nd
	10	nd	nd	nd	* 15.34 ± 0.47 ^f^	nd	nd	nd	nd

Note: nd—not detectable. Results are given in mm as the mean ± SD. Inhibition zones, including the diameter of the hole (8 mm). Values with different letters (a–g) within one sample with different concentrations are statistically different at *p* < 0.05, according to Tukey’s test. *—inhibition zone with some bacteria.

**Table 3:** The antifungal activity of GTEs against some yeast and mold species.

Extracts	*S. cerevisiae*	*A. niger*	*A. flavus*	*A. ochraceus*
Rid-GTE	24.55 ± 0.87 *	15.41 ± 2.12 (+++) **	11.56 ± 0.63 (++) **	nd
Psp-GTE	nd	23.75 ± 0.58 (+++) **	nd	20.51 ± 0.92 (+++) **
Hrh-GTE	nd	nd	nd	nd
Rca-GTE	nd	23.72 ± 0.79 (++) **	11.72 ± 0.18 (+) **	nd
Vid-GTE	nd	nd	nd	nd
Cox-GTE	nd	19.08 ± 0.48 (+++) **	nd	16.67 ± 1.42 (+) **
Bsv-GTE	nd	nd	nd	18.38 ± 1.01 (+) **
Vvi-GTE	nd	nd	nd	nd

Note: nd—not detectable; *—supported growth zone, **—accelerated/premature sporulation zone; (+)—few spores; (++)—medium number of spores; (+++)—many spores. Results are expressed as the mean ± SD in mm. Inhibition zones, including the diameter of the hole (8 mm).

**Table 4:** The GTEs generated antimicrobial effect in the case of tested microbial strains.

Microorganisms		Rca-GTE			Hrh-GTE			Vid-GTE			Psp-GTE			Vvi-GTE			Cox-GTE			Rid-GTE			Bsv-GTE		
Gram- positive bacteria	*B. cereus* ^R1^	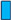	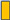	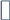	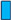	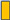	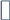	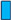	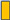	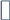	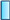	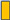	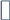	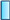	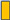	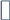	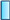	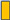	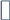	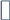	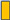	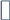	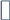	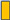	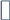
	*E. faecalis* ^R2^	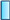	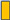	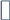	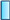	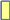	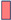	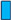	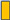	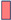	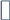	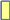	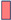	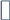	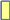	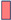	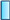	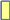	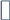	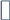	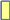	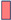	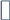	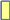	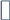
	*L. monocytogenes*	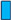	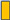	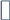	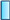	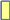	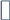	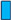	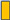	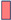	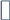	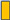	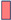	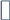	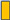	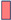	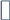	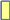	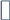	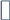	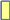	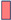	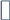	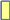	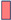
	*S. aureus* ^R2^	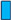	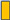	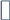	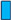	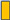	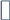	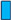	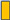	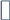	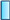	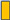	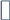	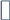	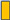	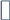	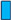	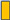	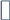	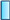	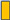	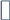	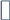	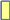	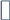
Gram- negative bacteria	*E. coli*	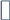	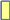	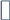	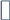	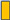	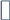	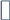	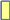	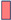	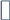	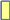	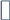	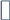	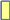	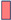	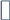	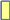	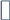	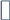	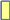	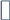	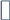	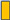	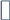
	*S. enterica*	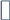	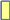	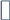	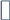	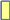	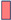	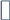	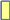	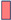	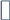	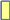	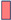	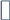	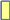	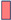	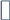	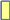	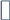	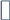	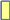	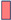	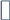	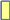	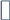
	*P. aeruginosa* ^R2,3^	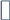	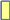	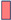	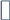	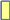	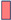	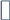	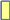	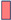	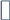	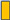	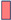	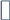	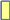	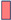	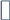	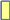	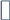	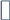	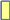	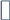	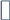	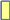	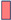
Yeasts	*S. cerevisiae*	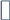	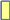	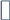	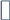	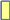	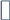	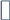	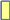	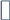	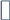	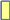	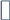	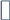	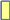	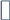	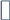	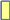	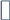	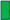	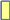	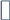	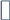	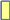	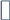
	*C. albicans*	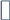	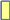	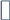	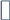	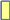	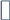	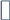	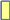	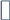	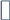	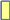	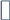	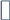	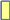	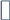	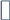	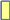	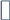	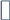	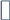	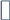	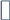	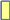	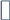
Molds	*A. niger*	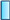	x	x	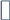	x	x	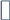	x	x	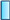	x	x	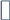	x	x	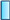	x	x	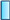	x	x	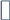	x	x
	*A. flavus*	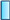	x	x	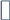	x	x	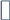	x	x	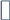	x	x	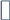	x	x	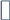	x	x	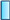	x	x	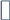	x	x
	*A. ochraceus*	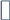	x	x	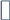	x	x	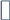	x	x	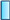	x	x	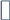	x	x	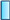	x	x	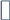	x	x	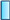	x	x
	*P. citrinum*	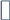	x	x	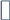	x	x	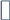	x	x	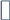	x	x	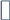	x	x	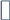	x	x	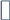	x	x	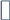	x	x
	*P. expansum*	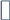	x	x	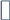	x	x	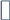	x	x	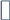	x	x	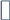	x	x	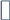	x	x	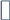	x	x	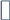	x	x

Note: 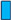 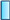 ADM; 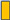 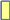 MIC; 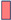 MMC; 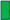 Growth promoting effect (ADM); 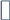 No effect; x—not tested. The concentration of GTEs: darker color: 10–40%, lighter color: 50–100%. ^R^*:* acquired antibiotic resistance of ^1^ cefotaxime, ^2^ kanamycin, ^3^ tetracycline.

**Table 5:** The composition of the mobile phase.

Time, min	Methanol	Water	2% Formic Acid in Water
0.00	5	90	5
3.00	15	70	15
6.00	15	70	15
9.00	21	58	21
13.00	21	58	21
18.00	30	41	29
22.00	30	41	29
26.00	50	0	50
29.00	50	0	50
29.01	5	90	5
35.00	5	90	5

**Table 6:** The main MS transitions of the standards.

Name of Standard	Retention Time, min	*m*/*z*, and Main Transition	MRM	Other Transitions
affeic acid	13.8	179.0 > 135.0	Negative	179.0 > 134.0 179.0 > 89.0
Carnosic acid	32.0	331.2 > 285.1	Negative	
Chlorogenic acid	11.9	353.0 > 191.0	Negative	
*trans-p*-coumaric acid	17.5	163.0 > 119.0	Negative	163.0 > 93.0
Ellagic acid	27.2	301.0 > 185.0	Negative	301.0 > 257.0
Ferulic acid	18.4	193.0 > 134.0	Negative	193.0 > 178.0
Gallic acid	7.0	168.9 > 125.0	Negative	
Salicylic acid	23.5	137.0 > 93.0	Negative	137.0 > 75.0 137.0 > 65.0
Apigenin	28.1	269.0 > 117.0	Negative	
Catechin	10.3	289.0 > 202.9	Negative	
Chrysin	29.7	253.0 > 143.0	Negative	253.0 > 119.0 253.0 > 107.0
Hyperoside	20.3	463.1 > 300.0	Negative	463.1 > 301.0
Kaempferol	27.9	285.0 > 187.0	Negative	285.0 > 151.0 285.0 > 133.0
Luteolin	26.8	287.0 > 153.0	Positive	
Luteolin-7-*O*-glucosid	19.9	447.0 > 284.9	Negative	
Naringenin	26.2	271.0 > 119.0	Negative	271.0 > 107.0
Quercetin	25.4	300.9 > 151.0	Negative	300.9 > 121.0
Quercitrin	22.1	447.0 > 229.9	Negative	
Rutoside	20.2	609.0 > 300.0	Negative	609.0 > 301.0 609.0 > 271.0
Vitexin	18.4	431.0 > 311.0	Negative	
Carnosol	30.7	329.1 > 285.1	Negative	
Arbutoside	6.0	317.1 > 109.0	Negative	317.1 > 271.0 317.1 > 161.0

**Table 7:** The standards used for LC/MS analysis.

Name of Standard	Concentration Range, mg/mL	Calibration Curve Equation	Correlation Factor	Detection Limit, mg/mL	Quantification Limit, mg/mL
Caffeic acid	0.11–1.10	Area = 4 × 10^7^ · conc[mg/mL] − 319,689	0.9998	3.20	4.80
Carnosic acid	0.28–2.80	Area = 10^7^ · conc[mg/mL] − 99,360	0.9994	4.00	6.00
Chlorogenic acid	0.13–1.30	Area = 2 × 10^8^ · conc[mg/mL] − 269,699	0.9997	5.00	8.00
*trans-p*-coumaric acid	0.16–1.60	Area = 3 × 10^7^ · conc[mg/mL] + 36,967	0.9995	2.50	4.90
Ellagic acid	0.107–1.070	Area = 14,987 · conc[mg/mL] − 138.52	0.9982	3.70	5.50
Ferulic acid	0.100–1.000	Area = 5 × 10^6^ · conc[mg/mL] − 50,000	0.9992	4.00	6.00
Gallic acid	0.107–1.070	Area = 8 × 10^6^ · conc[mg/mL] − 37,131	0.9999	1.90	2.80
Salicylic acid	0.16–1.60	Area = 4 × 10^7^ · conc[mg/mL] + 44,120	0.9997	1.50	2.00
Apigenin	0.10–0.98	Area = 2 × 10^8^ · conc[mg/mL] + 15,916	0.9999	0.20	0.30
Catechin	0.10–1.01	Area = 5 × 10^6^ · conc[mg/mL] − 1706	0.9984	1.00	2.00
Chrysin	0.10–1.00	Area = 1 × 10^8^ · conc[mg/mL] – 82,818	0.9997	3.00	5.00
Hyperoside	0.012–0.107	Area = 4 × 10^8^ · conc[mg/mL] − 567,182	0.9986	0.60	0.90
Kaempferol	0.10–1.00	Area = 10^7^ · conc[mg/mL] − 20,574	0.9996	0.80	1.20
Luteolin	0.01–0.10	Area = 2 × 10^8^ · conc[mg/mL] − 2295.4	0.9977	0.05	0.07
Luteolin-*7-O*-glucosid	0.07–0.70	Area = 1 × 10^9^ · conc[mg/mL] − 700,317	0.9990	3.00	4.00
Naringenin	0.16–1.60	Area = 3 × 10^8^ · conc[mg/mL] − 43,443	0.9999	0.60	0.90
Quercetin	0.09–0.91	Area = 5 × 10^7^ · conc[mg/mL] − 9556	0.9964	0.80	1.10
Quercitrin	0.16–1.60	Area = 395,509 · conc[mg/mL] − 1532.9	0.9992	1.60	2.30
Rutoside	0.17–1.70	Area = 2 × 10^8^ · conc[mg/mL] − 191,937	0.9996	4.00	6.00
Vitexin	0.17–1.70	Area = 3 × 10^8^ · conc[mg/mL] − 10^6^	0.9996	1.30	2.00
Carnosol	0.022–0.220	Area = 10^9^ × conc[mg/mL] − 253,279	0.9997	1.00	2.00
Arbutoside	0.11–1.10	Area = 6 × 10^7^ · conc[mg/mL] + 38,705	0.9984	1.30	2.60

## Data Availability

The original contributions presented in this study are included in the article and Supplementary Materials. Further inquiries can be directed to the corresponding authors.

## Supplementary Materials

The following supporting information can be downloaded at: https://www.mdpi.com/article/10.3390/antibiotics14101052/s1. The supporting materials contain the LC/MS chromatograms and MS spectra for boxwood GTE (Figures S1–S5), hawthorn GTE (Figures S6–S14), dog rose GTE (Figures S15–S26), raspberry GTE (Figures S27–S40), lingonberry GTE (Figures S41–S54), common grape GTE (Figures S55–S70), blackthorn GTE (Figures S71–S88) and sea buckthorn GTE (Figures S89–Figure S100).

## Abbreviations

The following abbreviations are used in this manuscript:

AAE	Ascorbic acid equivalent
ABTS	2,2′-azino-bis(3-ethylbenzothiazoline-6-sulfonic acid)
ADM	Agar diffusion method
BMM	Broth microdilution method
Bsv	Buxus sempervirens L. (boxwood)
CE	Catechin equivalent
Cox	Crataegus oxyacantha L. (hawthorn)
CT	Condensed tannins
CTC	Condensed tannin content
DPPH	2,2-diphenyl-1-picrylhydrazyl
GAE	Gallic acid equivalent
GTE	Gemmotherapy extract
Hrh	Hippophae rhamnoides L. (sea buckthorn)
MBC	Minimum bactericidal concentration
MFC	Minimum fungicidal concentration
MIC	Minimum inhibitory concentration
MMC	Minimum microbicidal concentration
OD	Optical density
Psp	Prunus spinosa L. (blackthorn)
Rca	Rosa canina L. (dog rose)
Rid	Rubus idaeus L. (raspberry)
TE	Trolox equivalent
TEAC	Trolox Equivalent Antioxidant Capacity
TFC	Total flavonoid content
TPC	Total phenolic content
TPTZ	2,4,6-tri(2-pyridinyl)-1,3,5-triazine
Vid	Vaccinium vitis-idaea L. (lingonberry)
Vvi	Vitis vinifera L. (common grape)

## Appendix A

**Table A1:** Zone size interpretative table (mm).

Name	Sensitive	Intermediate	Resistant	Source
Tetracycline 30 mcg				
*Enterobacteriaceae*	>15	12–14	<11	HiMedia
*Staphylococcus*, *Enterococcus* spp.	>19	15–18	<14	HiMedia
*Listeria*	>13.8		<13.8	[147]
*Bacillus*	>23	19–22	<18	[148]
Kanamycin 30 mcg				
*Enterobacteriaceae*, *Staphylococcus*	>18	14–17	<13	HiMedia
*Listeria*	>11		<11	[147]
*Bacillus*	No data			
Cefotaxime 30 mcg				
*Enterobacteriaceae*	>26	23–25	<22	HiMedia
*P. aeruginosa*, *Staphylococcus*	>23	15–22	<14	HiMedia
*Listeria*	No data			
