# Supplementary material for: Gemmotherapy Extracts Like the Dog Rose, Lingonberry, Sea Buckthorn, Blackthorn, Common Grape, Hawthorn, Raspberry and Boxwood Feature Variable Yet Excelling Antimicrobial Effects
Source: Antibiotics (Basel). 2025 Oct 21;14(10):1052. doi: 10.3390/antibiotics14101052 (PMC12561055; doi:10.3390/antibiotics14101052)
Supplement: Supplementary file 1 [file antibiotics-14-01052-s001.zip › antibiotics-3881570-supplementary.pdf]

LC/MS chromatograms and associated MS spectra

Boxwood (Bsv) GTE:

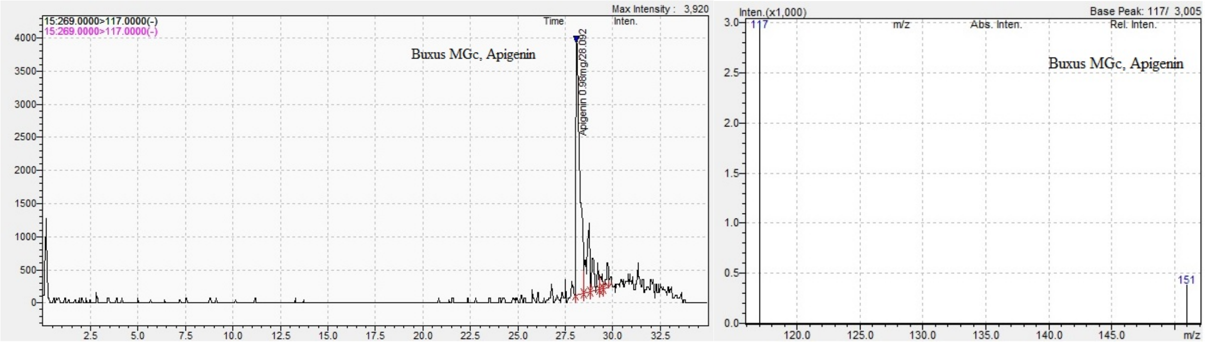

Figure S1. Boxwood (Bsv) GTE chromatogram and associated MS spectrum of apigenin

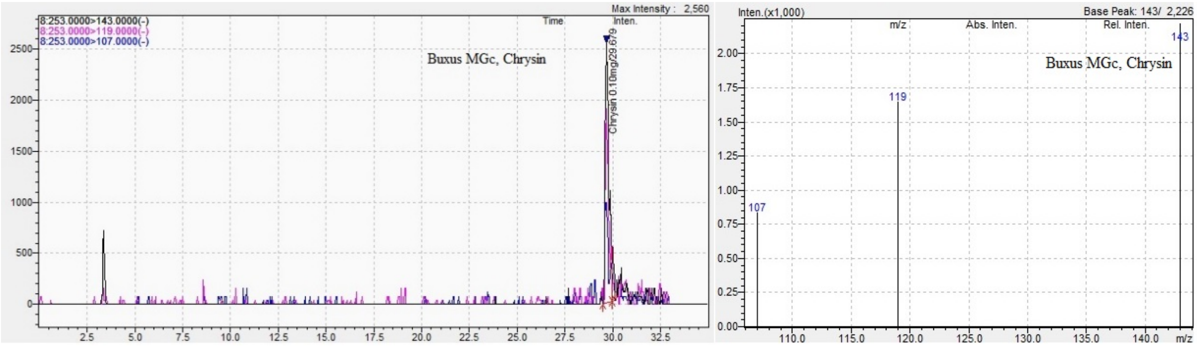

Figure S2. Boxwood (Bsv) GTE chromatogram and associated MS spectrum of chrysin

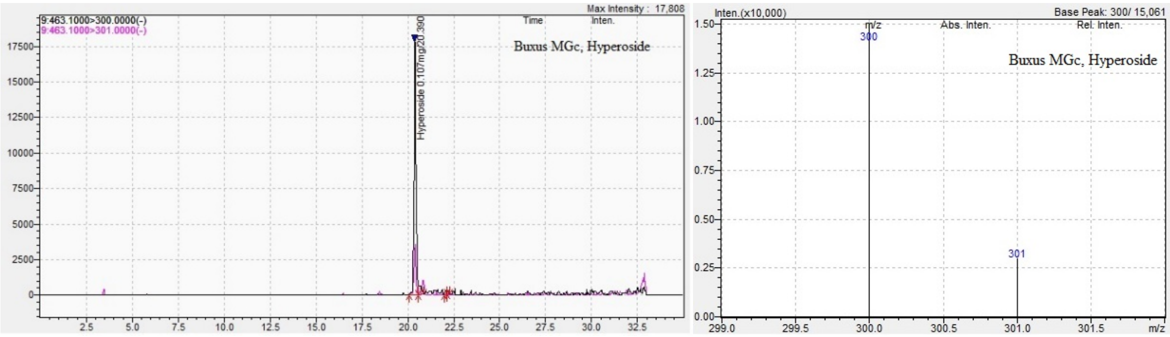

Figure S3. Boxwood (Bsv) GTE chromatogram and associated MS spectrum of hyperoside

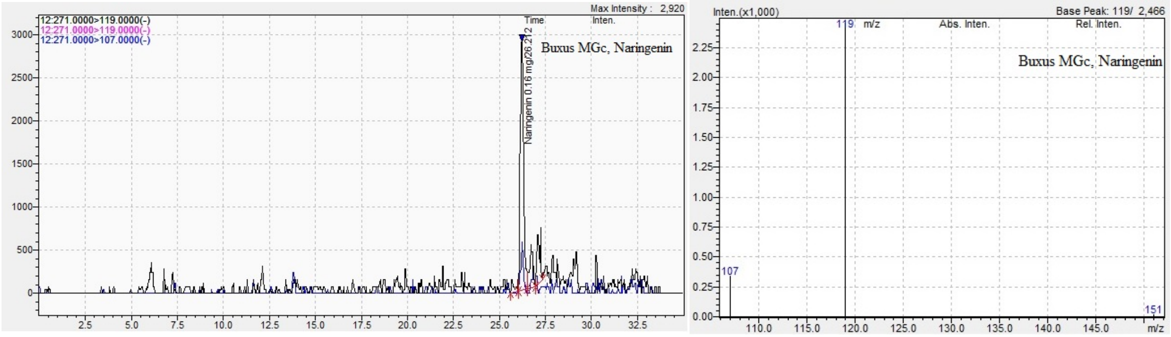

Figure S4. Boxwood (Bsv) GTE chromatogram and associated MS spectrum of naringenin

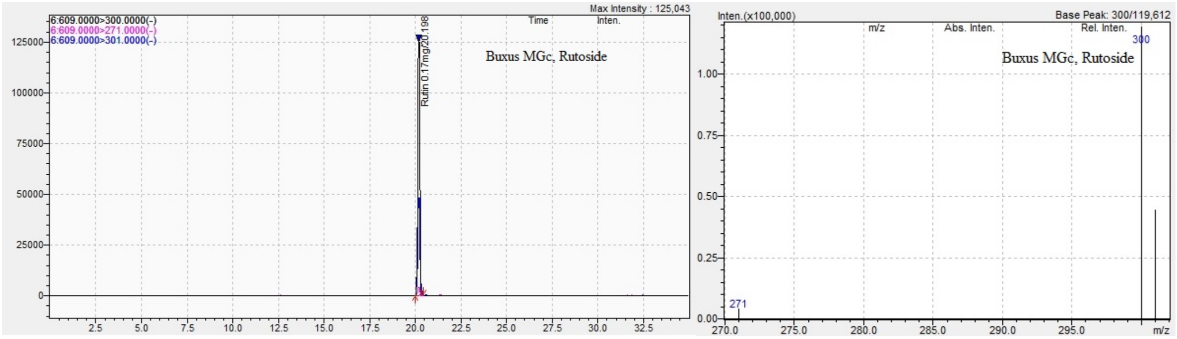

Figure S5. Boxwood (Bsv) GTE chromatogram and associated MS spectrum of rutoside

Hawthorn (Cox) GTE:

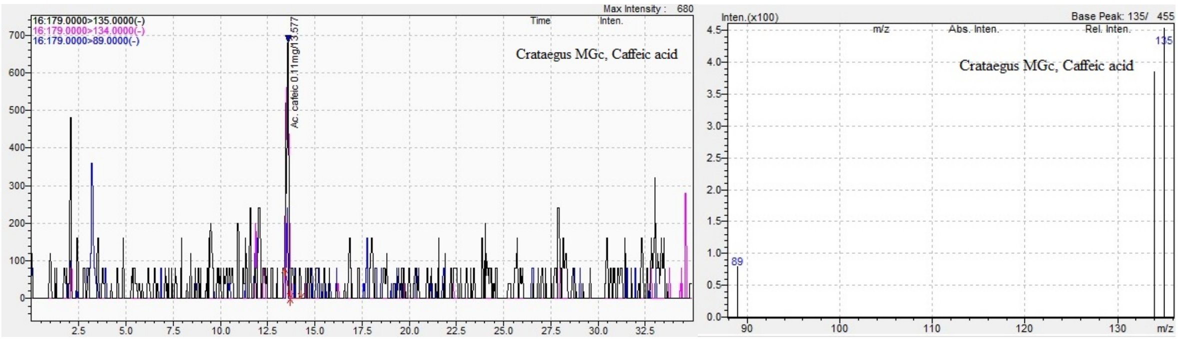

Figure S6. Hawthorn (Cox) GTE chromatogram and associated MS spectrum of caffeic acid

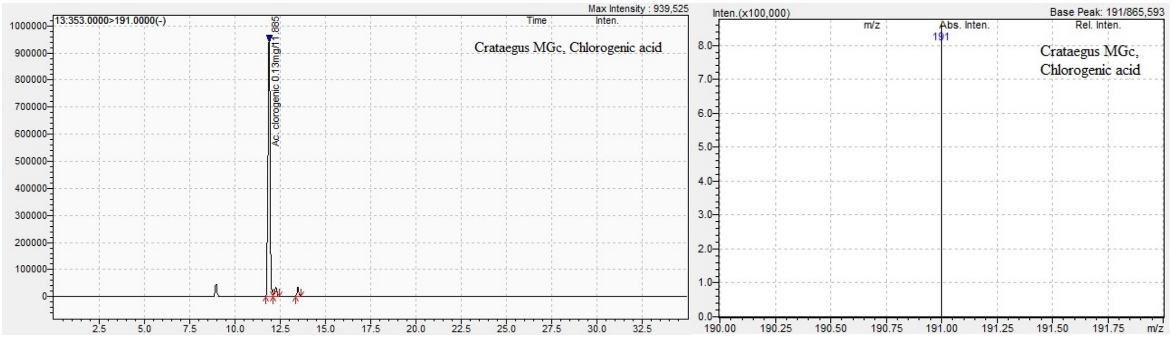

Figure S7. Hawthorn (Cox) GTE chromatogram and associated MS spectrum of chlorogenic acid

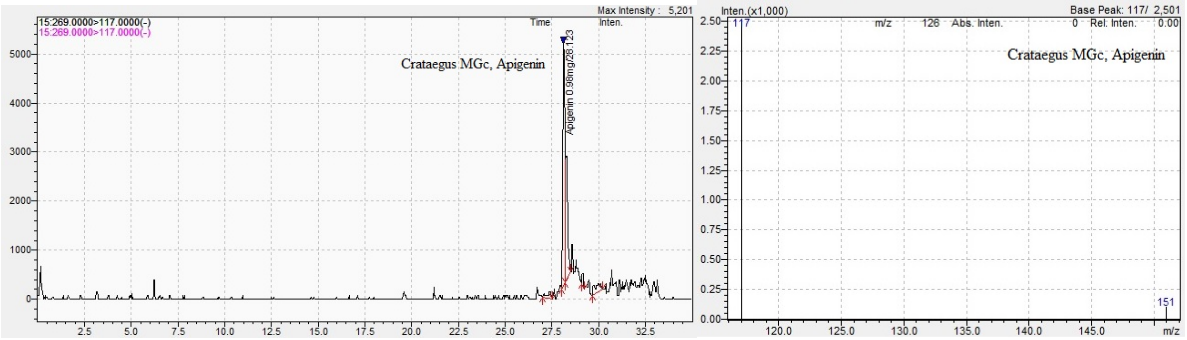

Figure S8. Hawthorn (Cox) GTE chromatogram and associated MS spectrum of apigenin

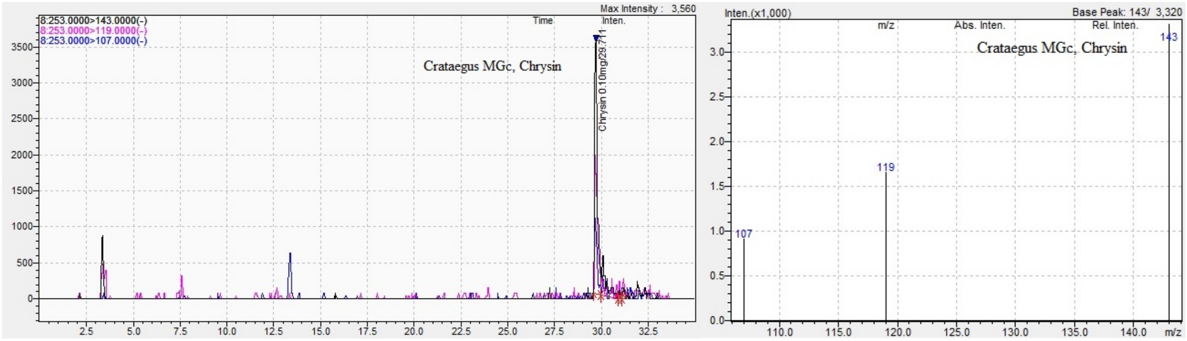

Figure S9. Hawthorn (Cox) GTE chromatogram and associated MS spectrum of chrysin

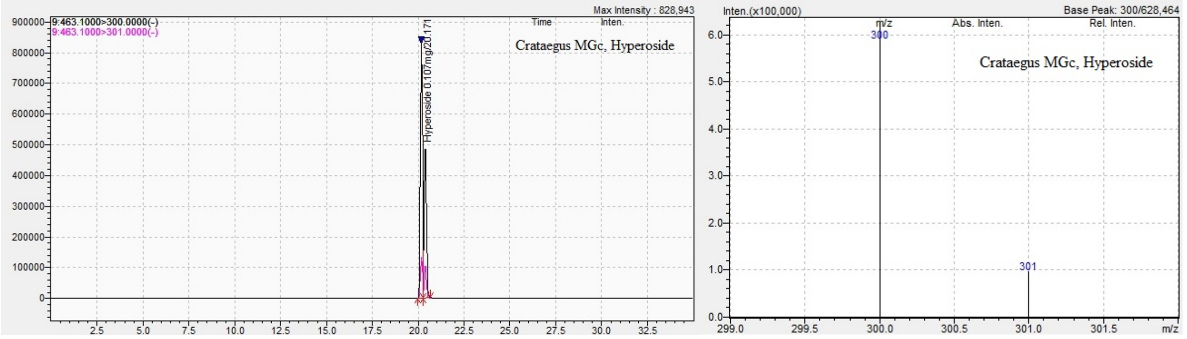

Figure S10. Hawthorn (Cox) GTE chromatogram and associated MS spectrum of hyperoside

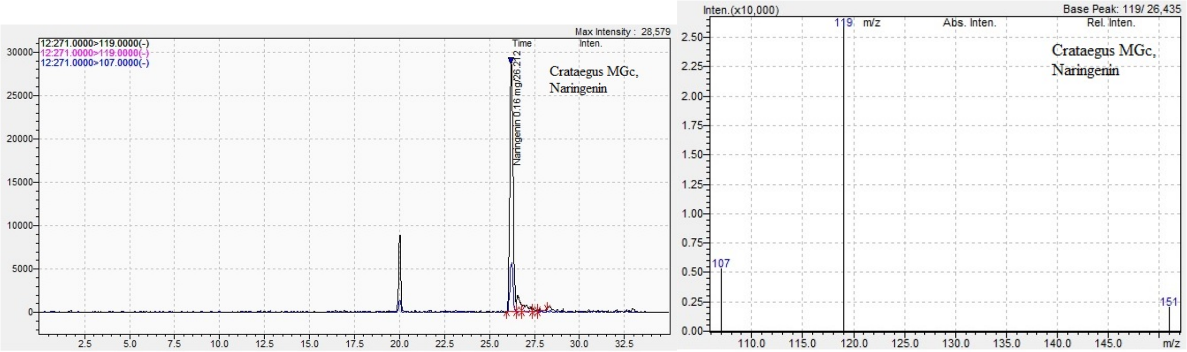

Figure S11. Hawthorn (Cox) GTE chromatogram and associated MS spectrum of naringenin

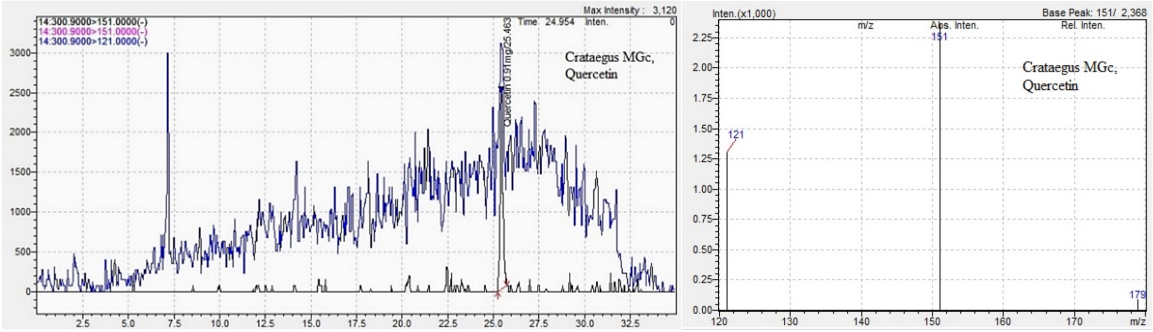

Figure S12. Hawthorn (Cox) GTE chromatogram and associated MS spectrum of quercetin

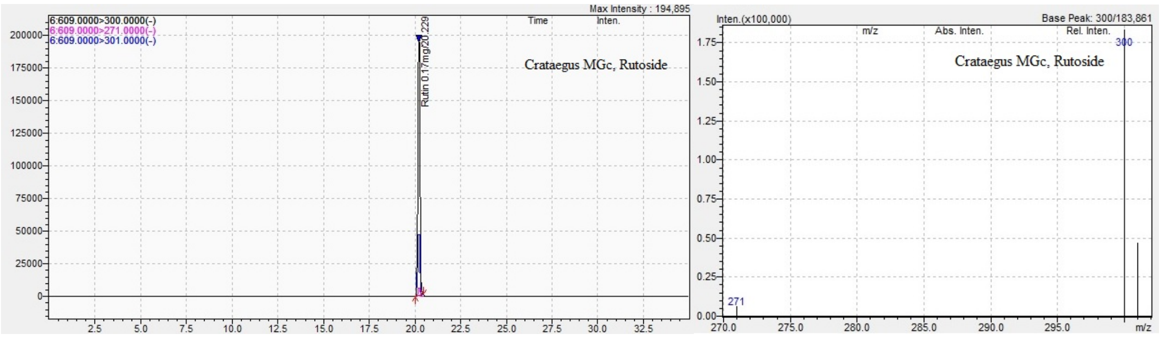

Figure S13. Hawthorn (Cox) GTE chromatogram and associated MS spectrum of rutoside

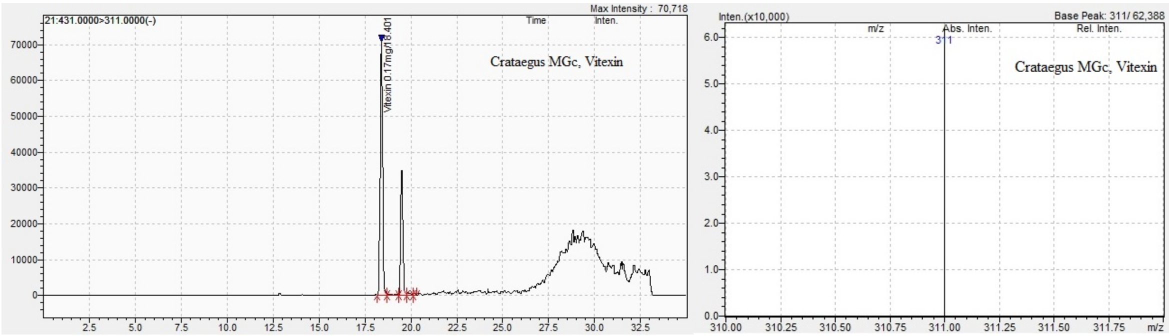

Figure S14. Hawthorn (Cox) GTE chromatogram and associated MS spectrum of vitexin

Dog rose (Rca) GTE:

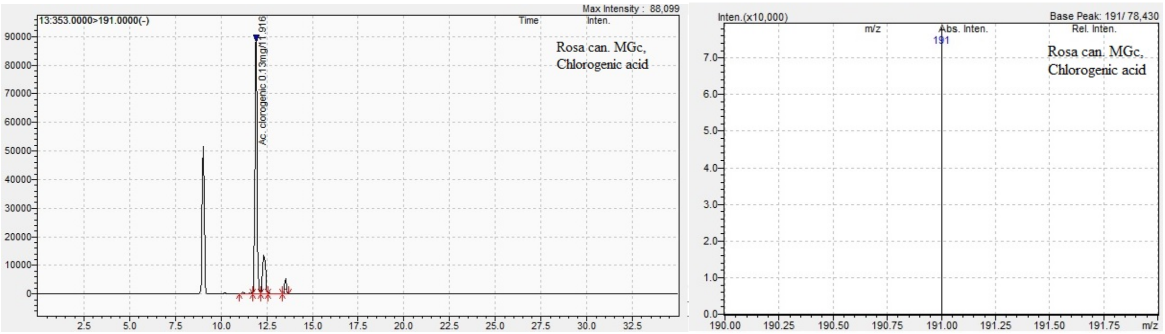

Figure S15. Dog rose (Rca) GTE chromatogram and associated MS spectrum of chlorogenic acid

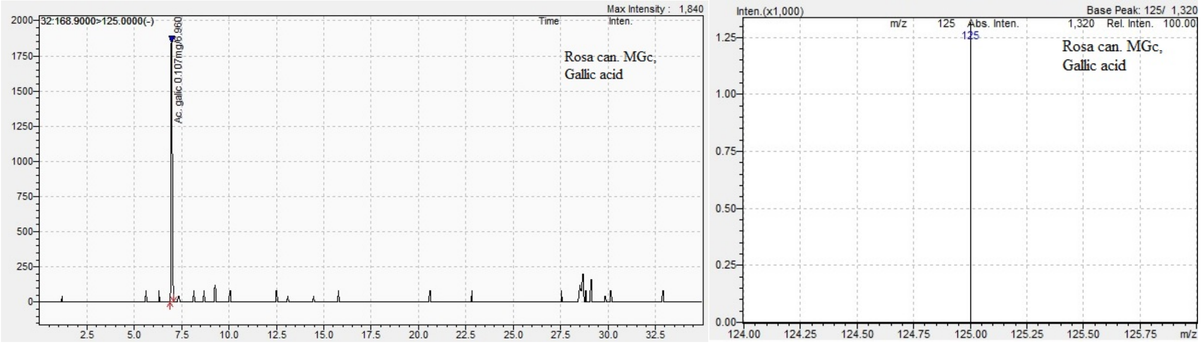

Figure S16. Dog rose (Rca) GTE chromatogram and associated MS spectrum of gallic acid

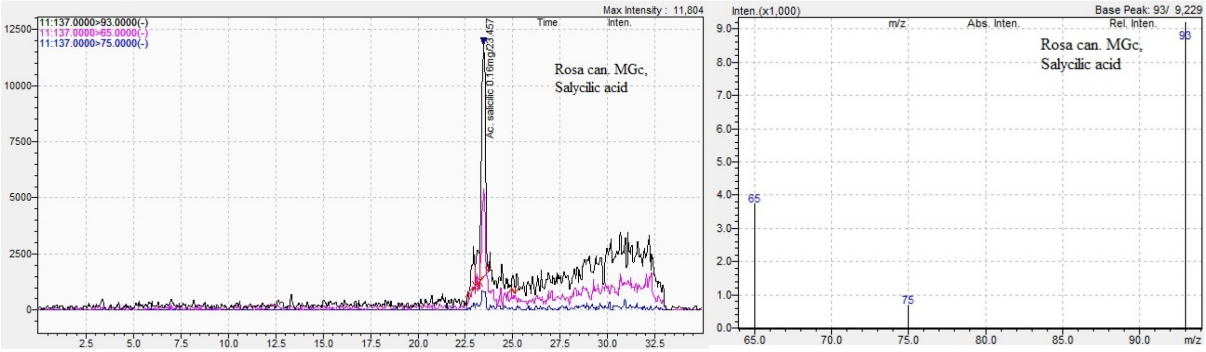

Figure S17. Dog rose (Rca) GTE chromatogram and associated MS spectrum of salycilic acid

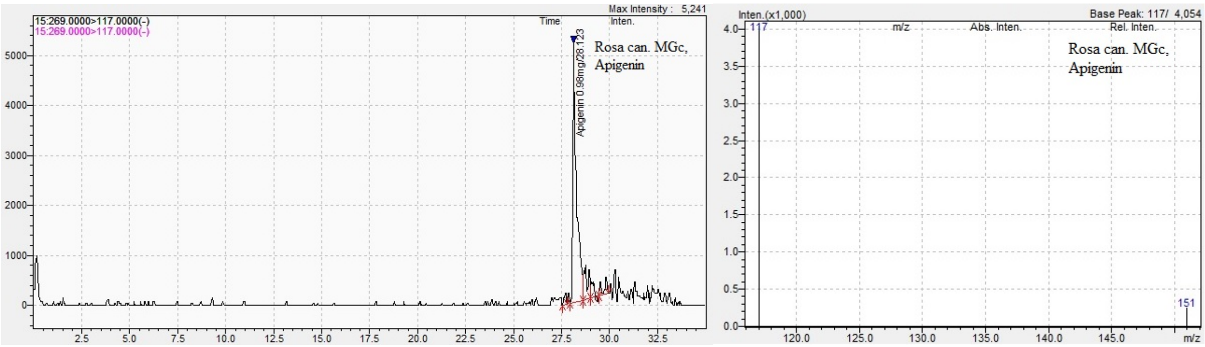

Figure S18. Dog rose (Rca) GTE chromatogram and associated MS spectrum of apigenin

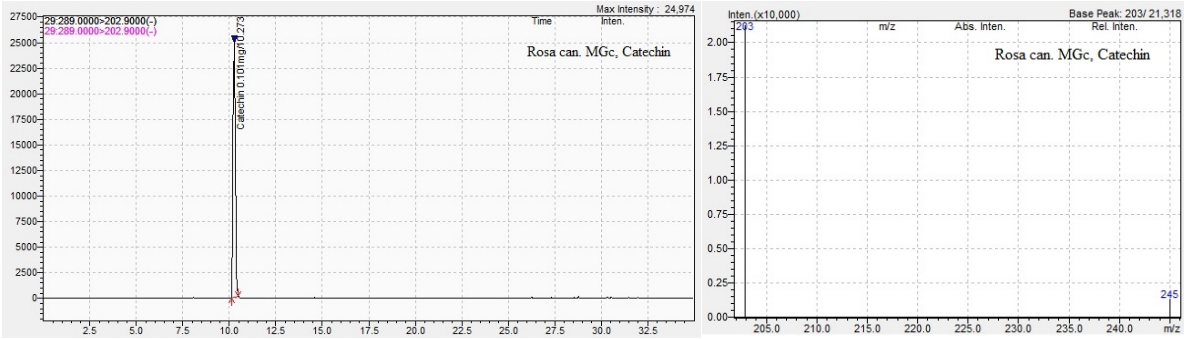

Figure S19. Dog rose (Rca) GTE chromatogram and associated MS spectrum of catechin

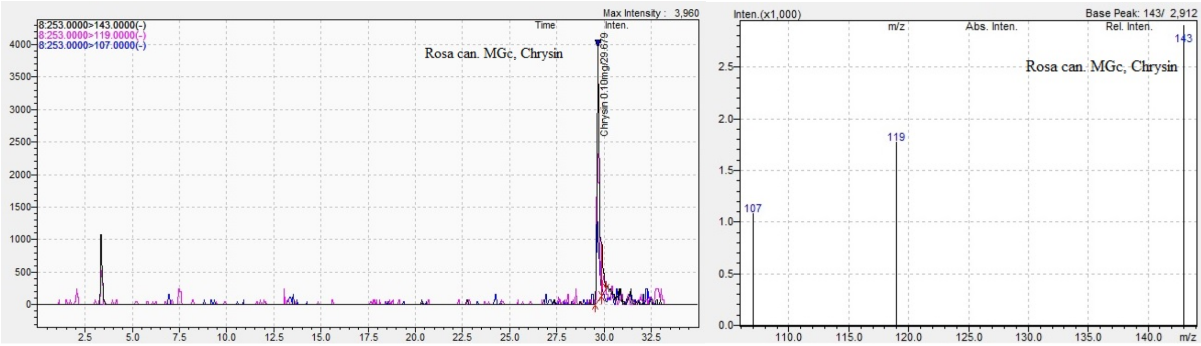

Figure S20. Dog rose (Rca) GTE chromatogram and associated MS spectrum of chrysin

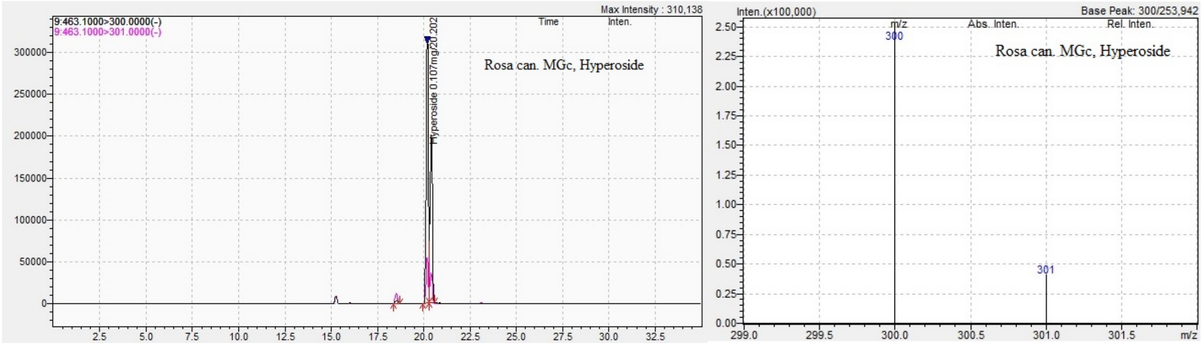

Figure S21. Dog rose (Rca) GTE chromatogram and associated MS spectrum of hyperoside

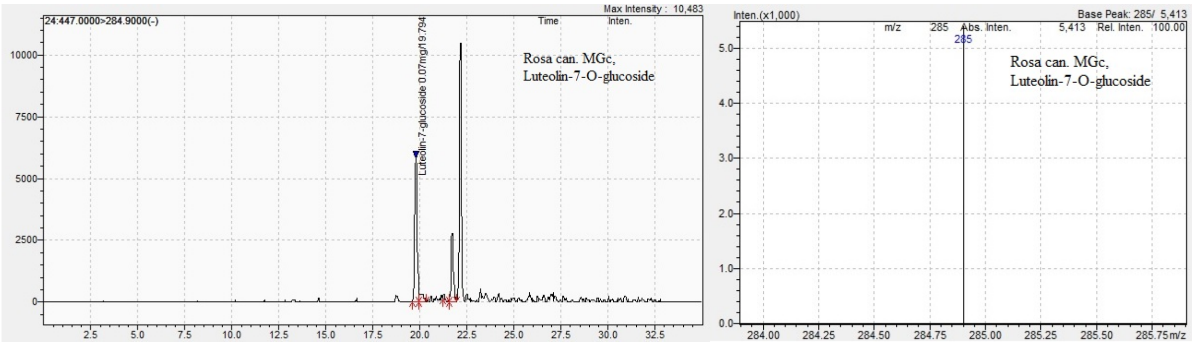

Figure S22. Dog rose (Rca) GTE chromatogram and associated MS spectrum of luteolin-7-O-glucoside

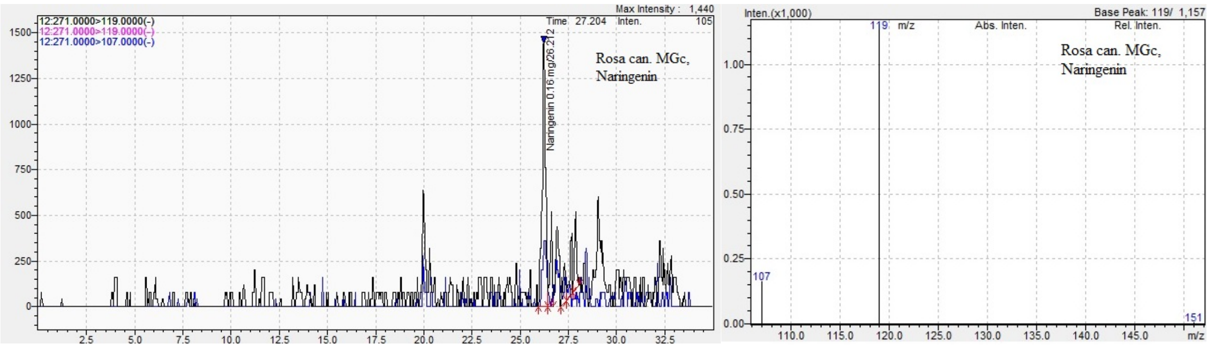

Figure S23. Dog rose (Rca) GTE chromatogram and associated MS spectrum of naringenin

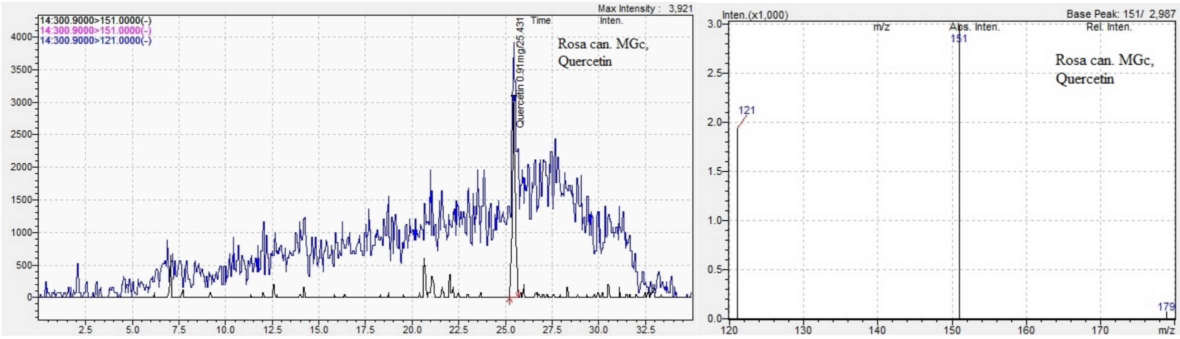

Figure S24. Dog rose (Rca) GTE chromatogram and associated MS spectrum of quercetin

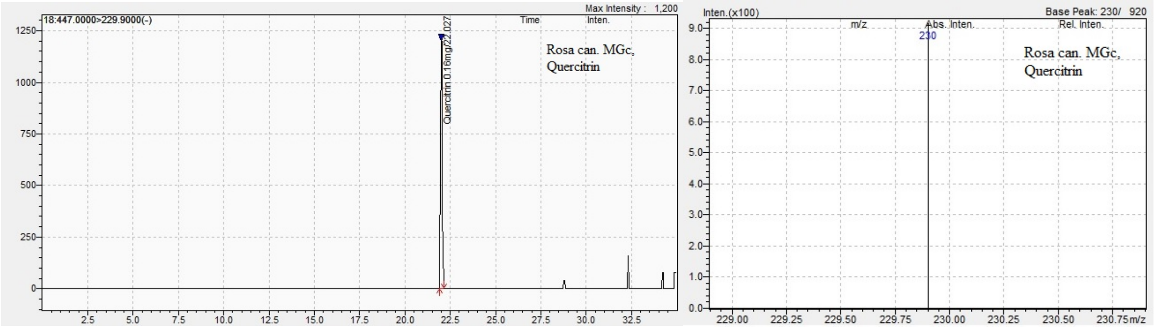

Figure S25. Dog rose (Rca) GTE chromatogram and associated MS spectrum of quercetin

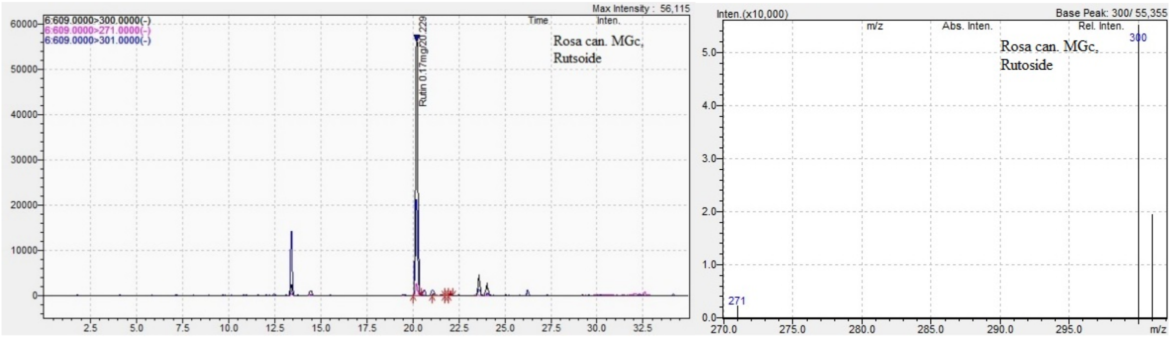

Figure S26. Dog rose (Rca) GTE chromatogram and associated MS spectrum of rutoside

Raspberry (Rid) GTE:

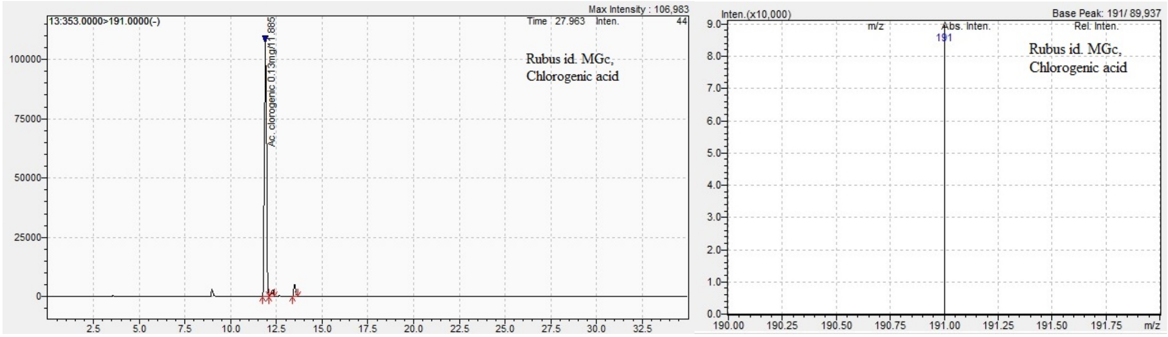

Figure S27. Raspberry (Rid) GTE chromatogram and associated MS spectrum of chlorogenic acid

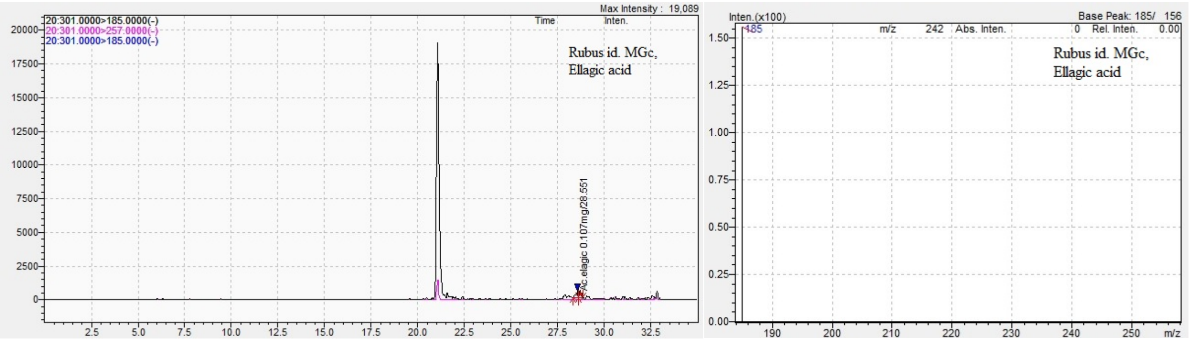

Figure S28. Raspberry (Rid) GTE chromatogram and associated MS spectrum of ellagic acid

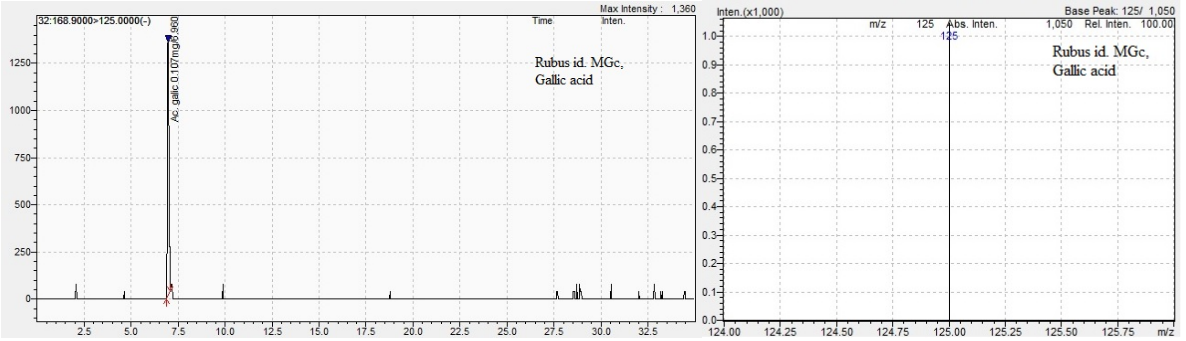

Figure S29. Raspberry (Rid) GTE chromatogram and associated MS spectrum of gallic acid

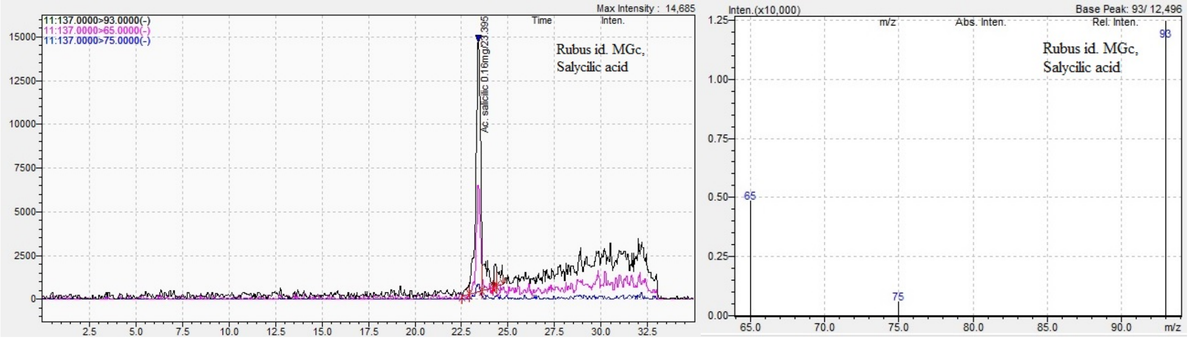

Figure S30. Raspberry (Rid) GTE chromatogram and associated MS spectrum of salycilic acid

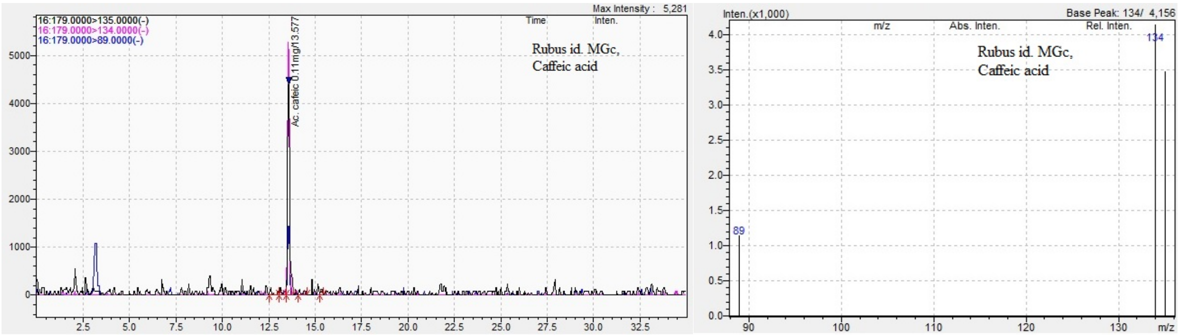

Figure S31. Raspberry (Rid) GTE chromatogram and associated MS spectrum of caffeic acid

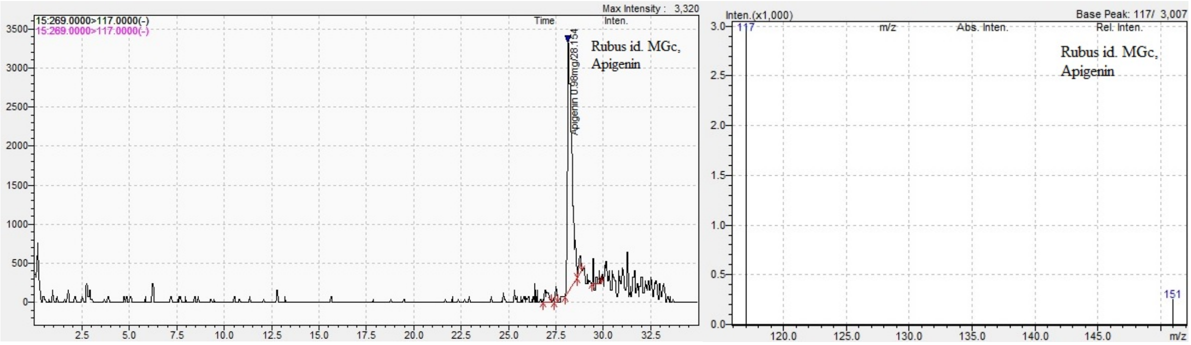

Figure S32. Raspberry (Rid) GTE chromatogram and associated MS spectrum of apigenin

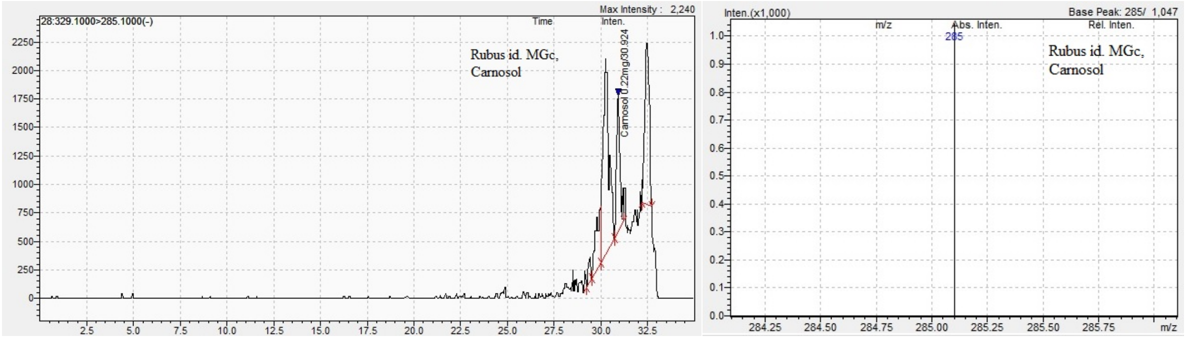

Figure S33. Raspberry (Rid) GTE chromatogram and associated MS spectrum of carnosol

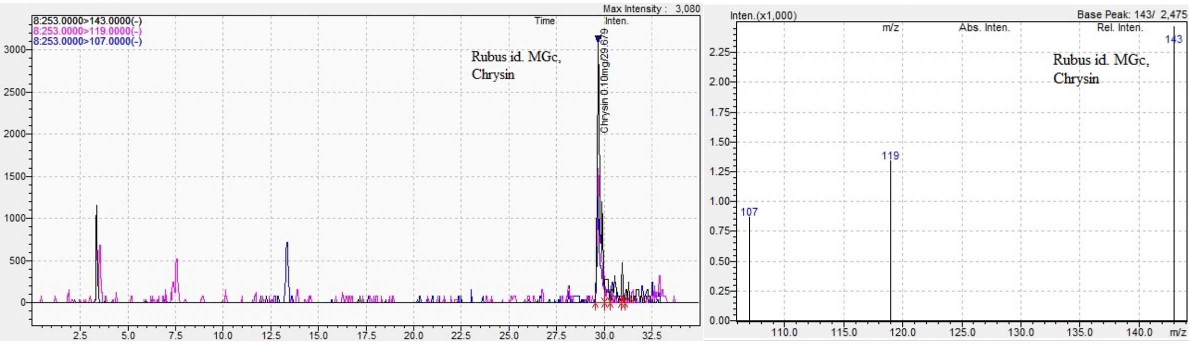

Figure S34. Raspberry (Rid) GTE chromatogram and associated MS spectrum of chrysin

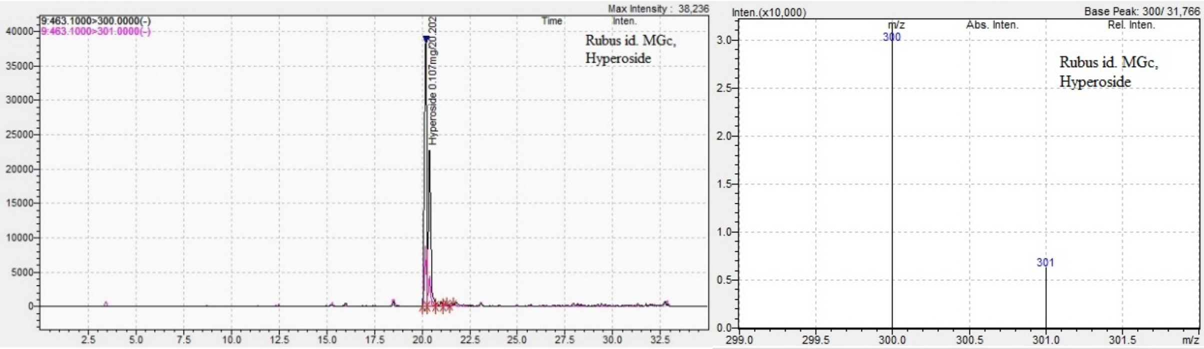

Figure S35. Raspberry (Rid) GTE chromatogram and associated MS spectrum of hyperoside

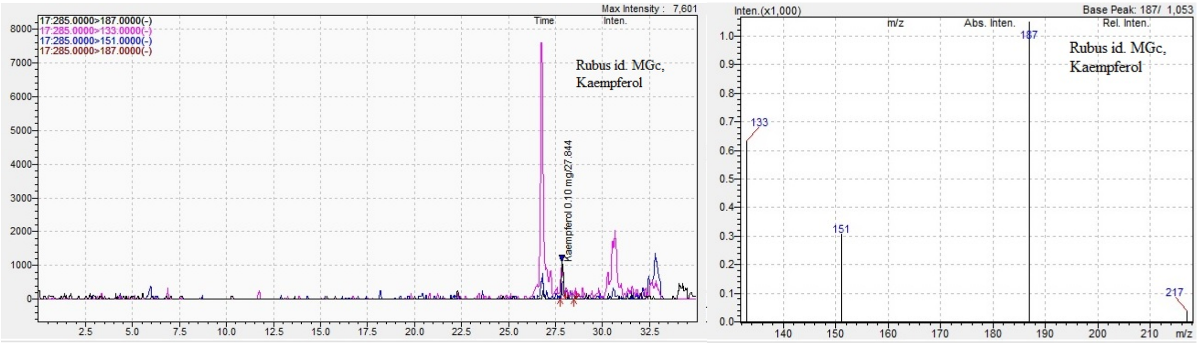

Figure S36. Raspberry (Rid) GTE chromatogram and associated MS spectrum of kaempferol

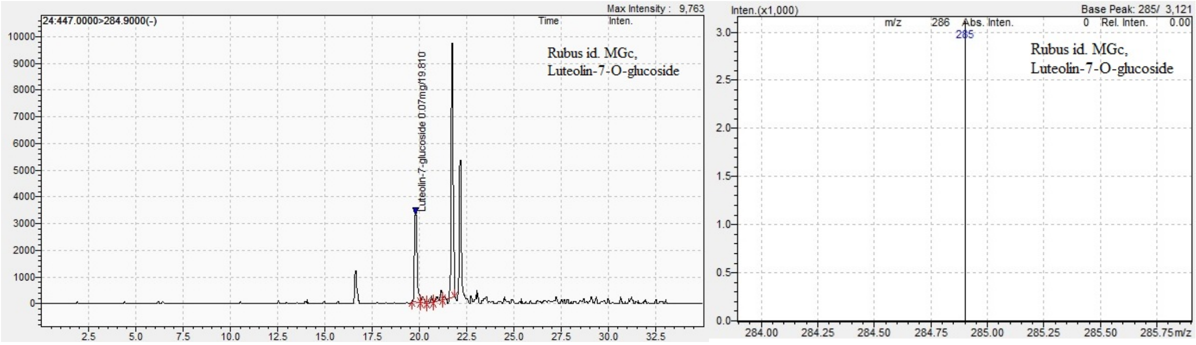

Figure S37. Raspberry (Rid) GTE chromatogram and associated MS spectrum of luteolin-7-O-glucoside

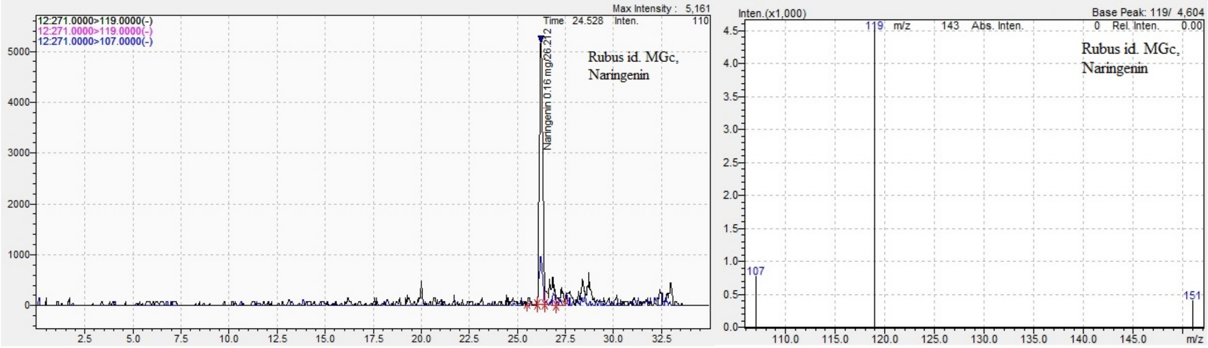

Figure S38. Raspberry (Rid) GTE chromatogram and associated MS spectrum of naringenin

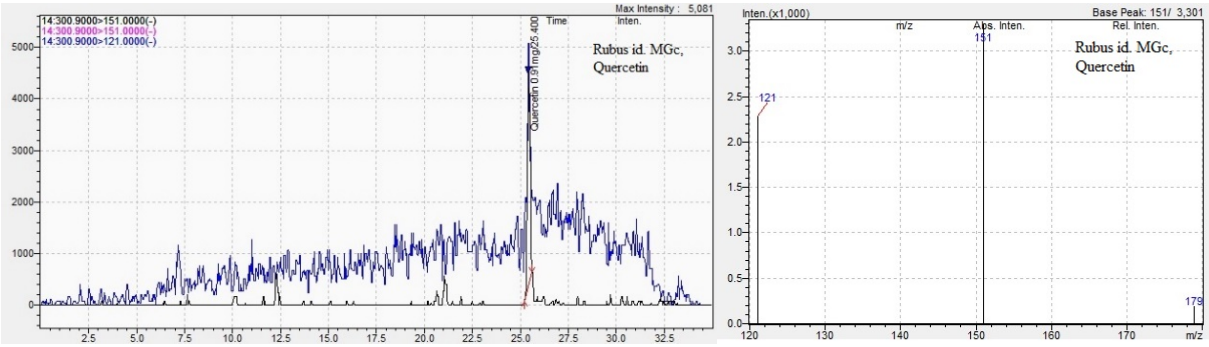

Figure S39. Raspberry (Rid) GTE chromatogram and associated MS spectrum of quercetin

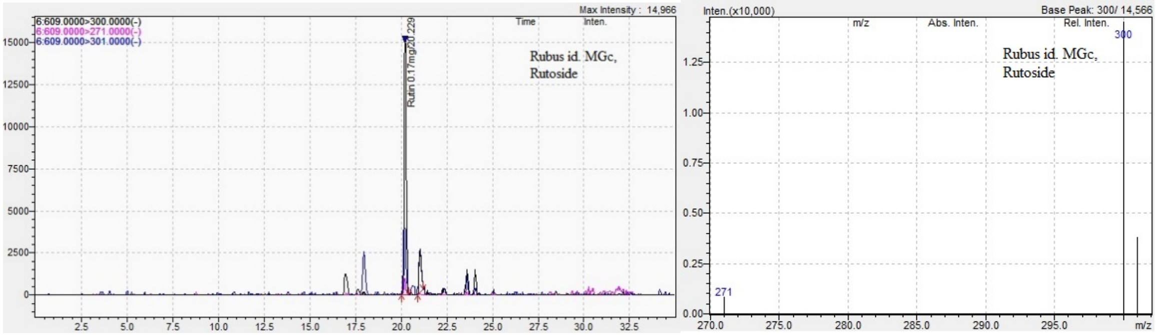

Figure S40. Raspberry (Rid) GTE chromatogram and associated MS spectrum of rutoside

Lingonberry (Vid) GTE:

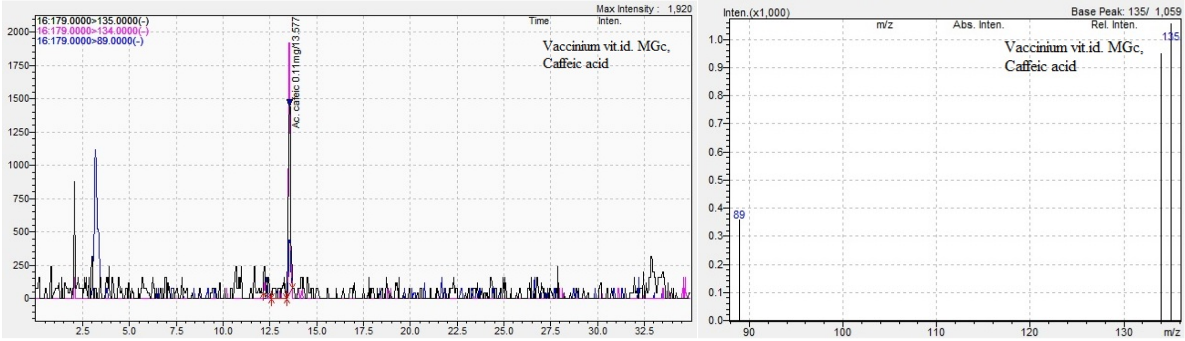

Figure S41. Lingonberry (Vid) GTE chromatogram and associated MS spectrum of caffeic acid

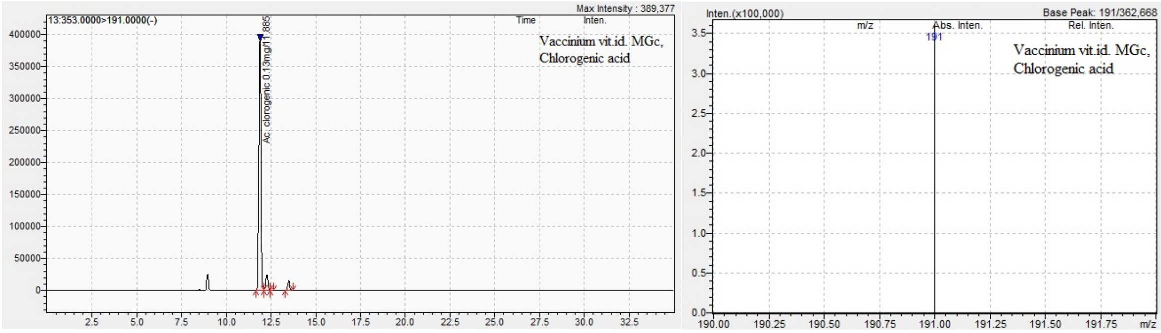

Figure S42. Lingonberry (Vid) GTE chromatogram and associated MS spectrum of chlorogenic acid

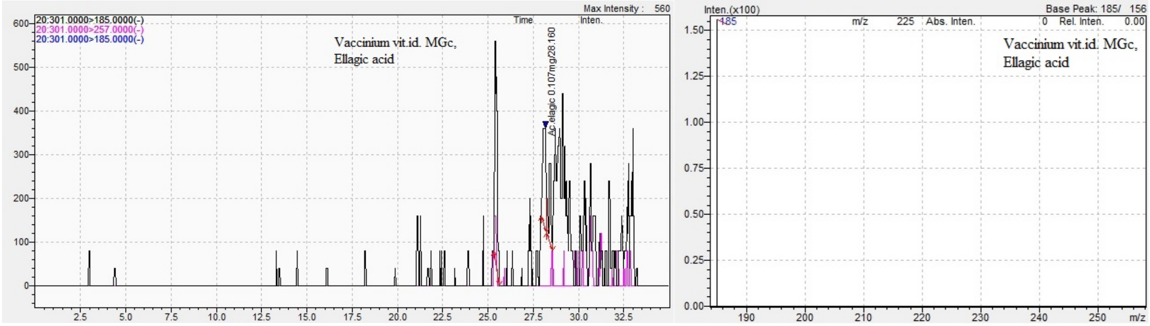

Figure S43. Lingonberry (Vid) GTE chromatogram and associated MS spectrum of ellagic acid

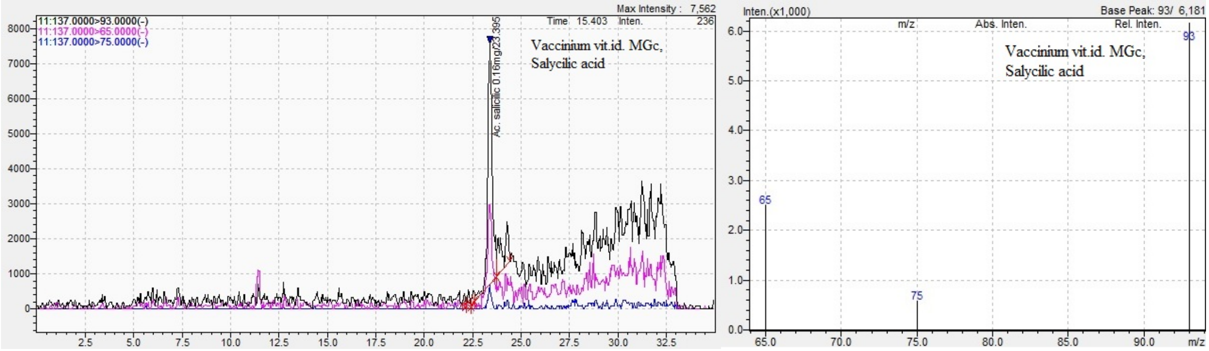

Figure S44. Lingonberry (Vid) GTE chromatogram and associated MS spectrum of salycilic acid

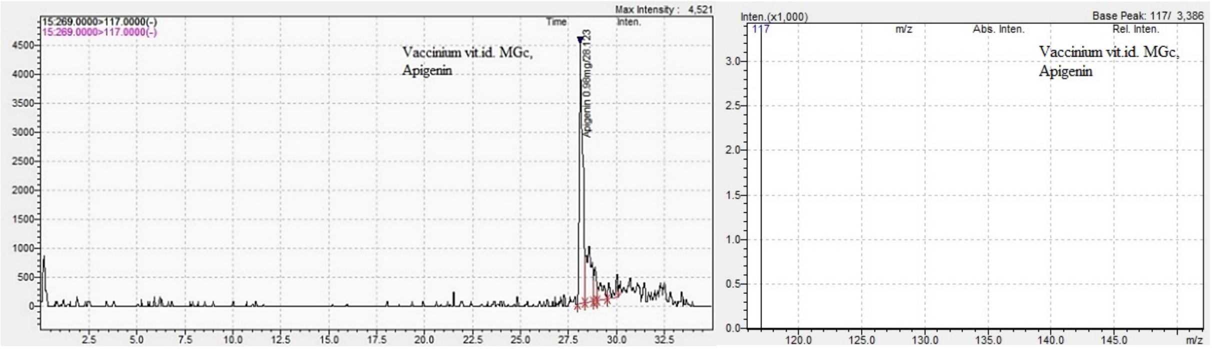

Figure S45. Lingonberry (Vid) GTE chromatogram and associated MS spectrum of apigenin

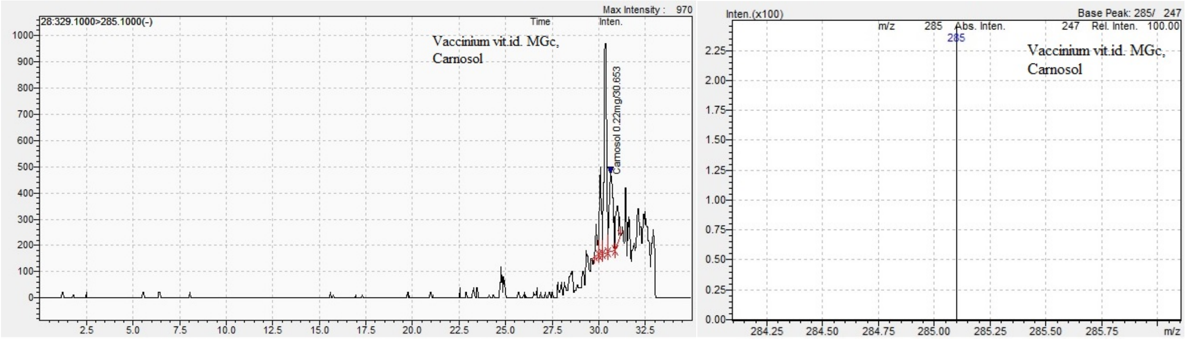

Figure S46. Lingonberry (Vid) GTE chromatogram and associated MS spectrum of carnosol

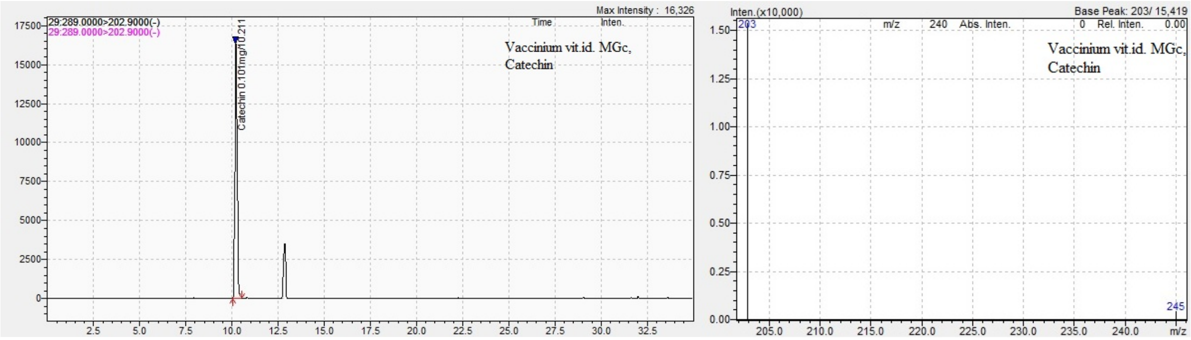

Figure S47. Lingonberry (Vid) GTE chromatogram and associated MS spectrum of catechin

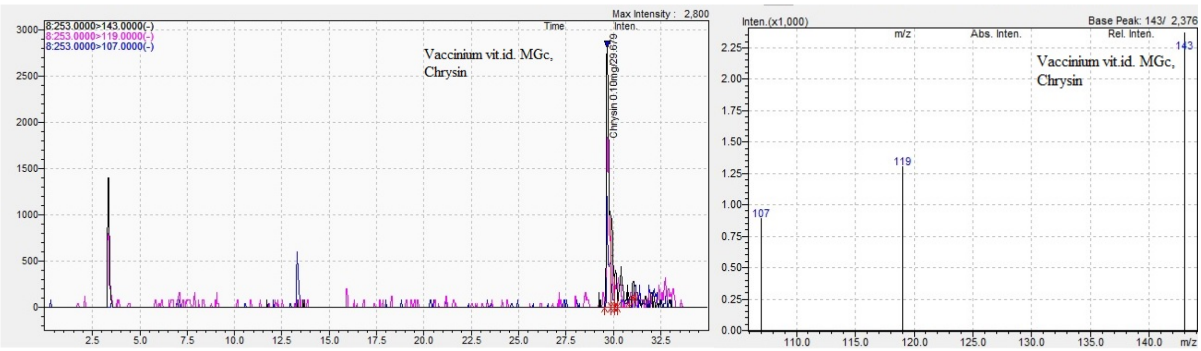

Figure S48. Lingonberry (Vid) GTE chromatogram and associated MS spectrum of chrysin

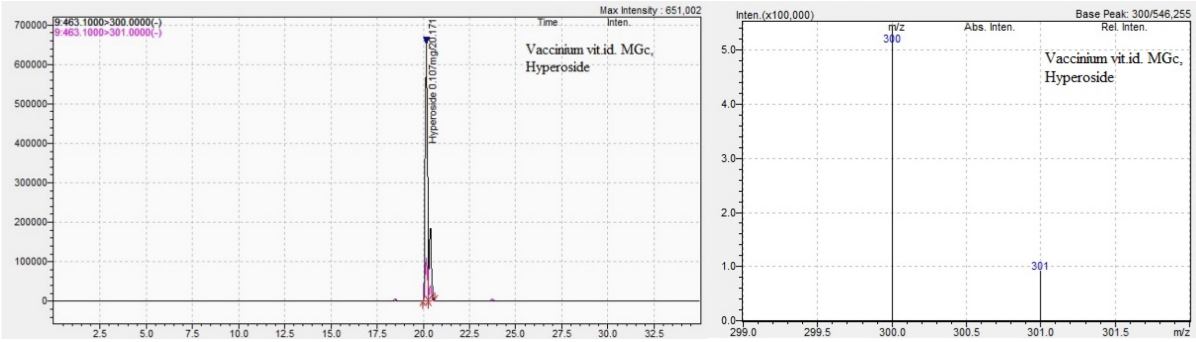

Figure S49. Lingonberry (Vid) GTE chromatogram and associated MS spectrum of hyperoside

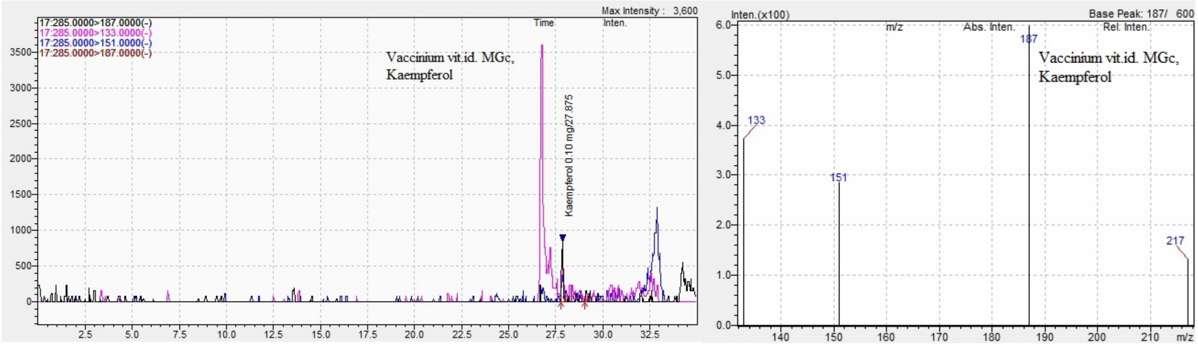

Figure S50. Lingonberry (Vid) GTE chromatogram and associated MS spectrum of kaempferol

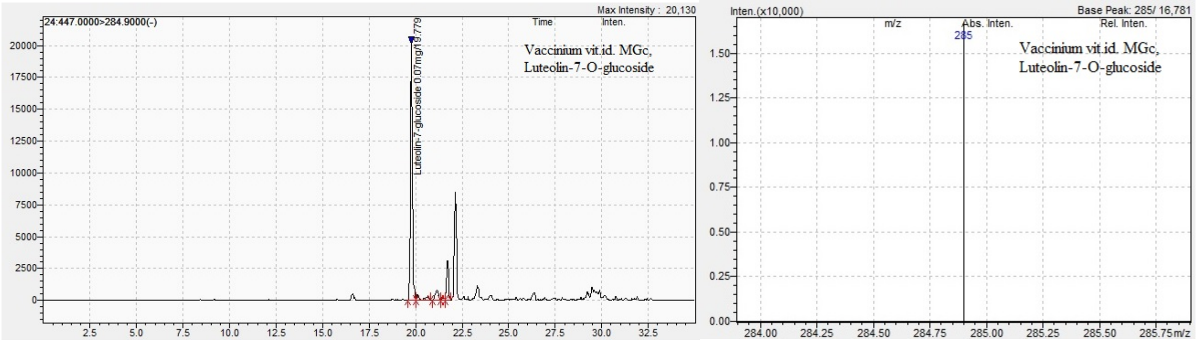

Figure S51. Lingonberry (Vid) GTE chromatogram and associated MS spectrum of luteolin-7-O-glucoside

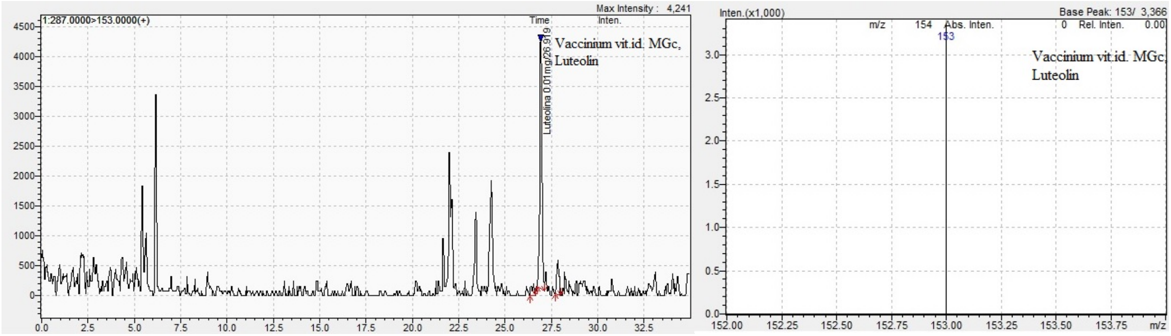

Figure S52. Lingonberry (Vid) GTE chromatogram and associated MS spectrum of luteolin

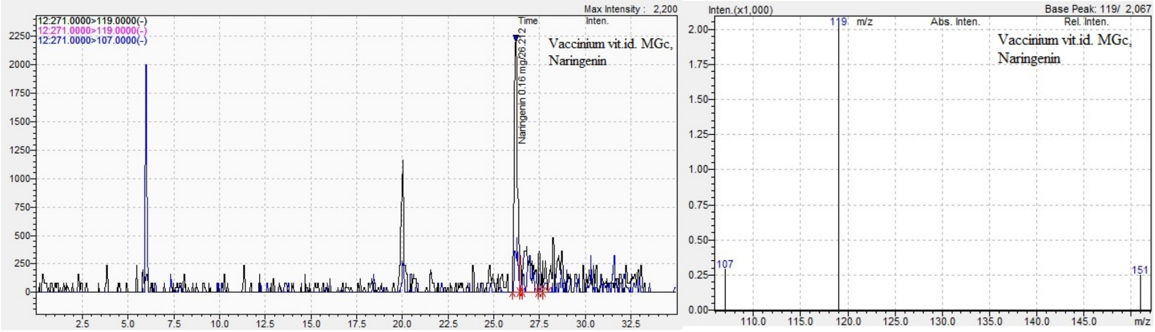

Figure S53. Lingonberry (Vid) GTE chromatogram and associated MS spectrum of naringenin

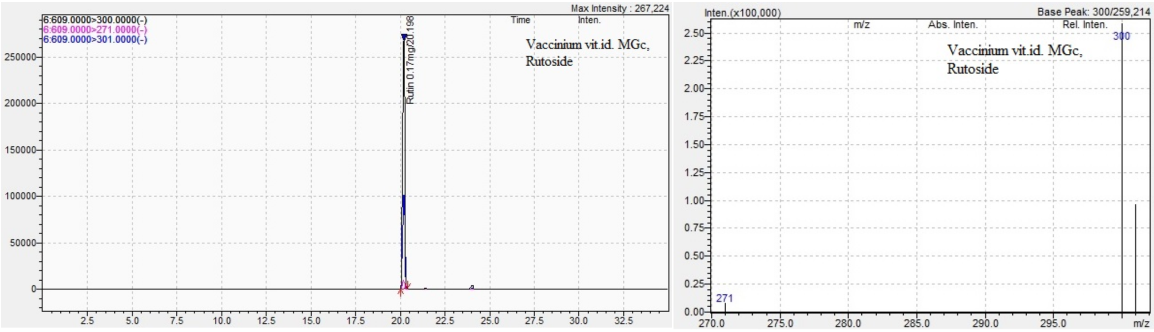

Figure S54. Lingonberry (Vid) GTE chromatogram and associated MS spectrum of rutoside

Common grape (Vvi) GTE:

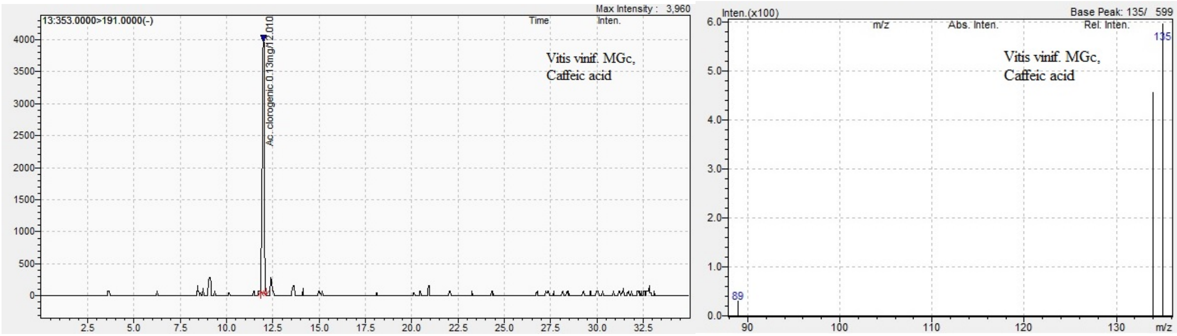

Figure S55. Common grape (Vvi) GTE chromatogram and associated MS spectrum of caffeic acid

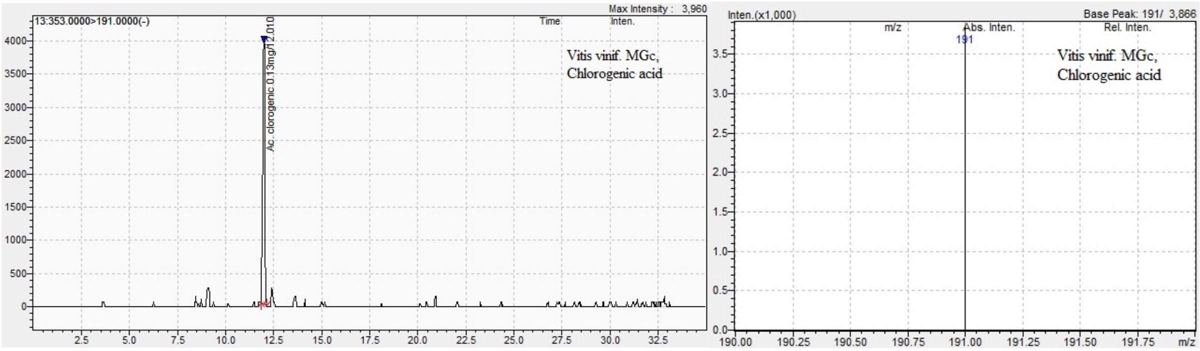

Figure S56. Common grape (Vvi) GTE chromatogram and associated MS spectrum of chlorogenic acid

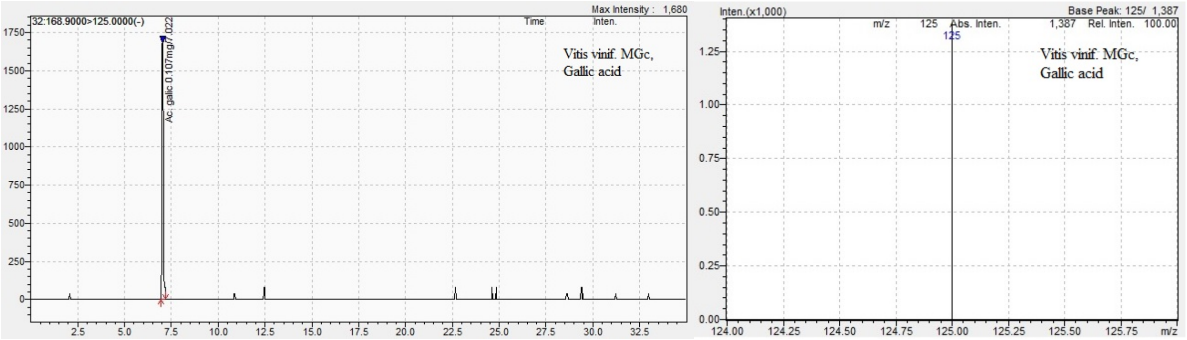

Figure S57. Common grape (Vvi) GTE chromatogram and associated MS spectrum of gallic acid

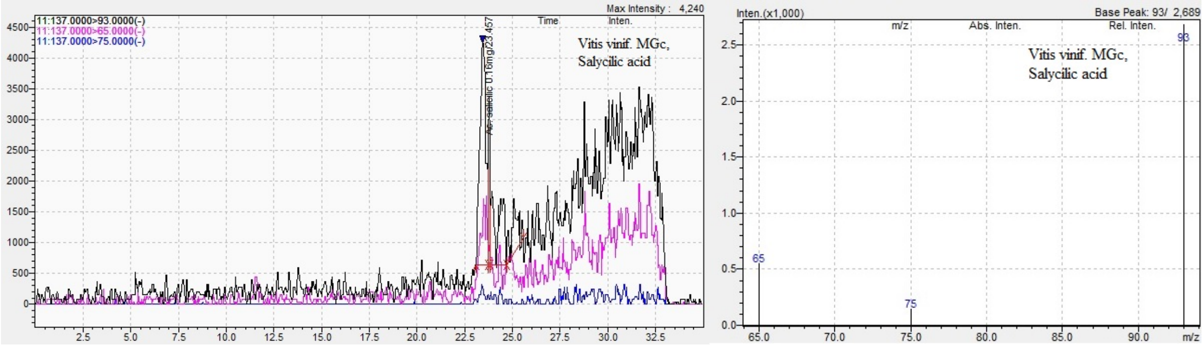

Figure S58. Common grape (Vvi) GTE chromatogram and associated MS spectrum of salycilic acid

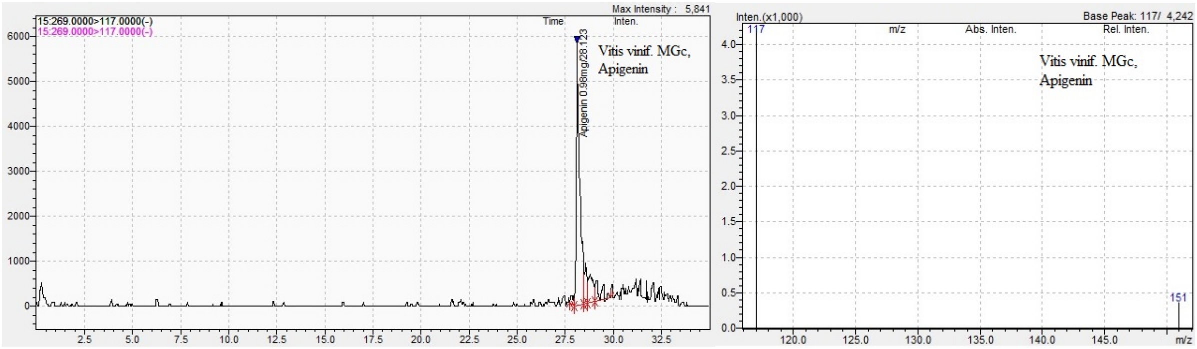

Figure S59. Common grape (Vvi) GTE chromatogram and associated MS spectrum of apigenin

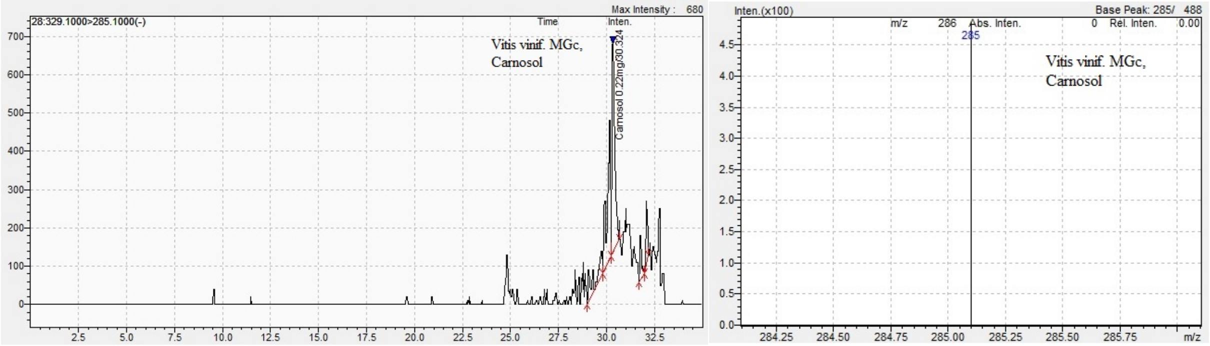

Figure S60. Common grape (Vvi) GTE chromatogram and associated MS spectrum of carnosol

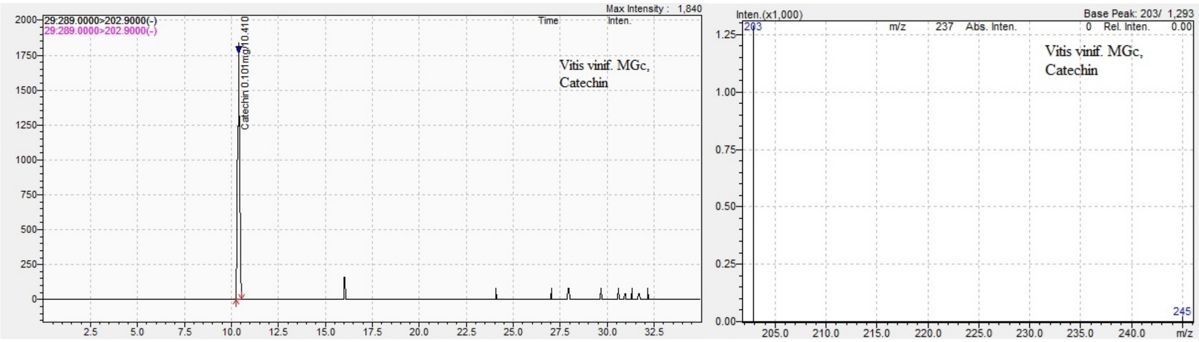

Figure S61. Common grape (Vvi) GTE chromatogram and associated MS spectrum of catechin

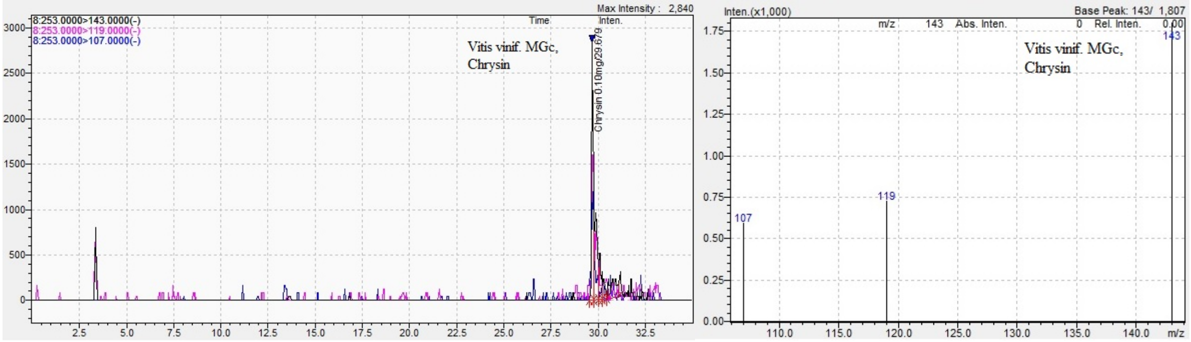

Figure S62. Common grape (Vvi) GTE chromatogram and associated MS spectrum of chrysin

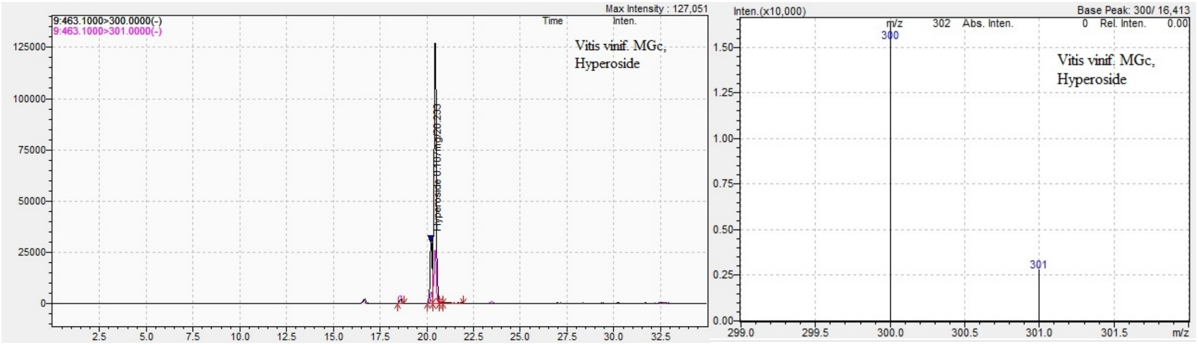

Figure S63. Common grape (Vvi) GTE chromatogram and associated MS spectrum of hyperoside

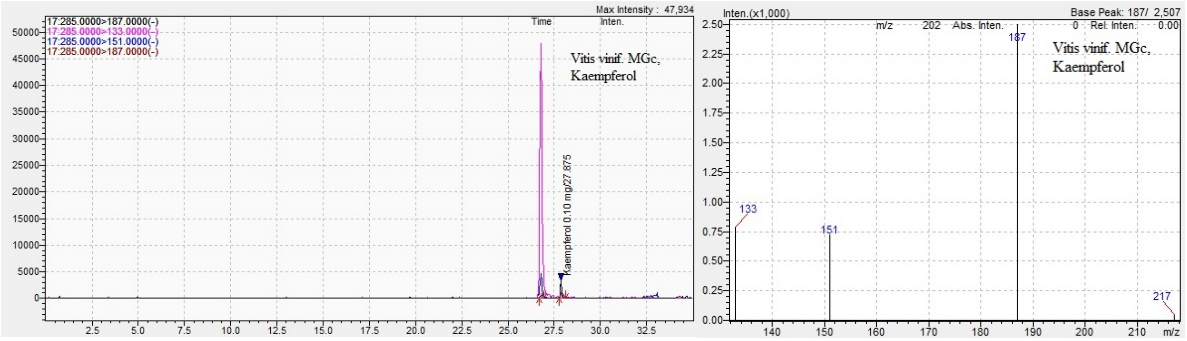

Figure S64. Common grape (Vvi) GTE chromatogram and associated MS spectrum of kaempferol

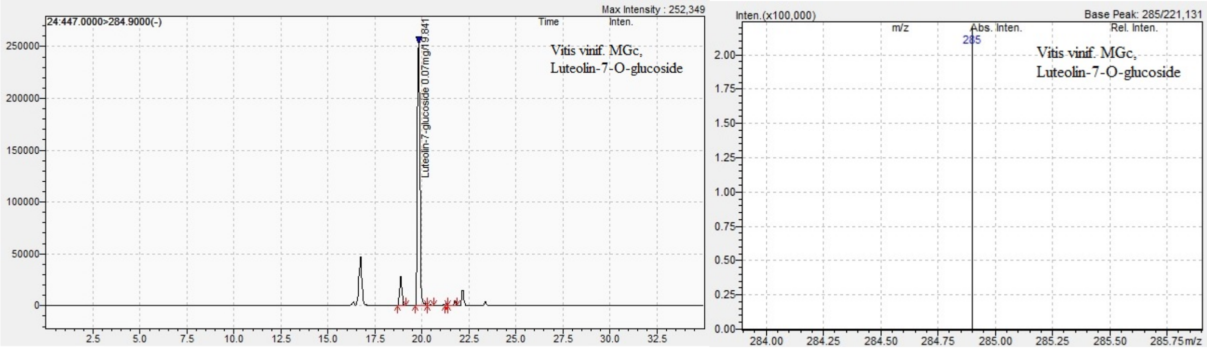

Figure S65. Common grape (Vvi) GTE chromatogram and associated MS spectrum of luteolin-7-o-glucoside

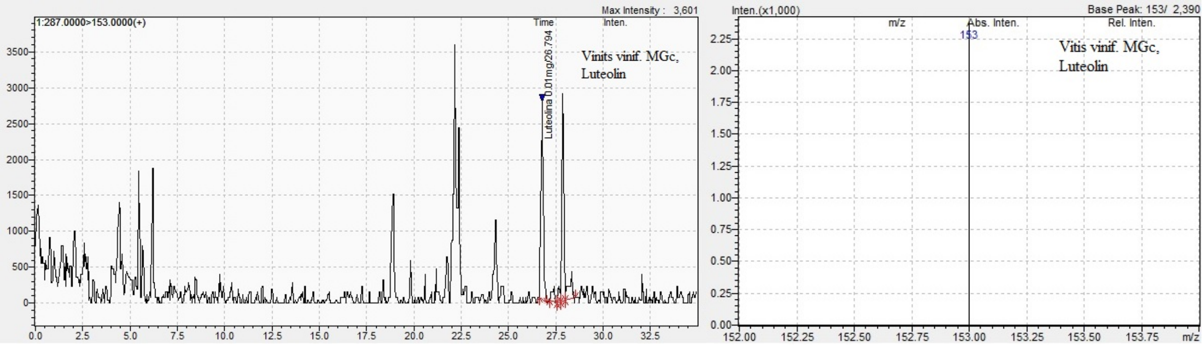

Figure S66. Common grape (Vvi) GTE chromatogram and associated MS spectrum of luteolin

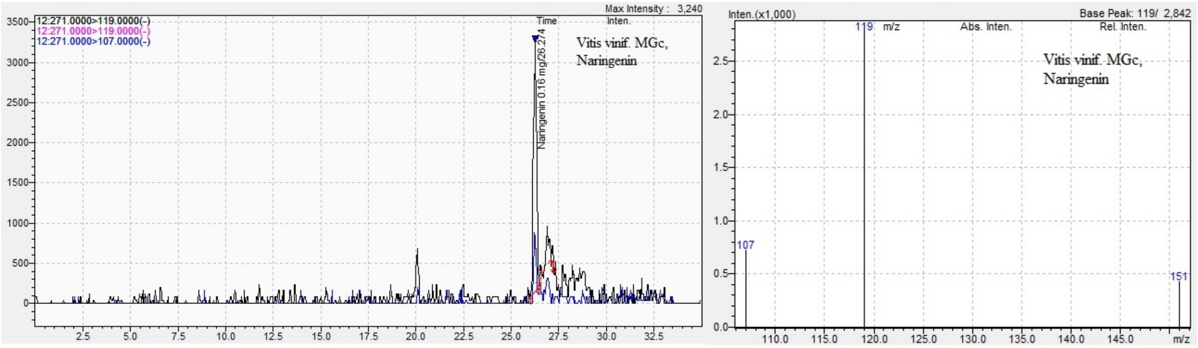

Figure S67. Common grape (Vvi) GTE chromatogram and associated MS spectrum of naringenin

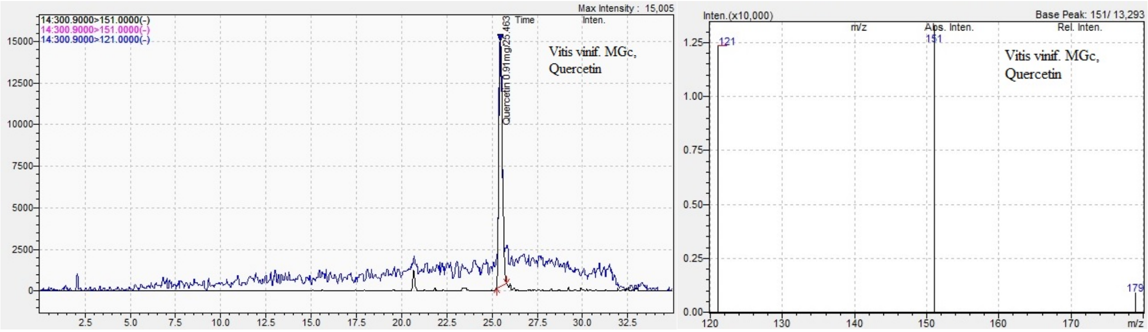

Figure S68. Common grape (Vvi) GTE chromatogram and associated MS spectrum of quercetin

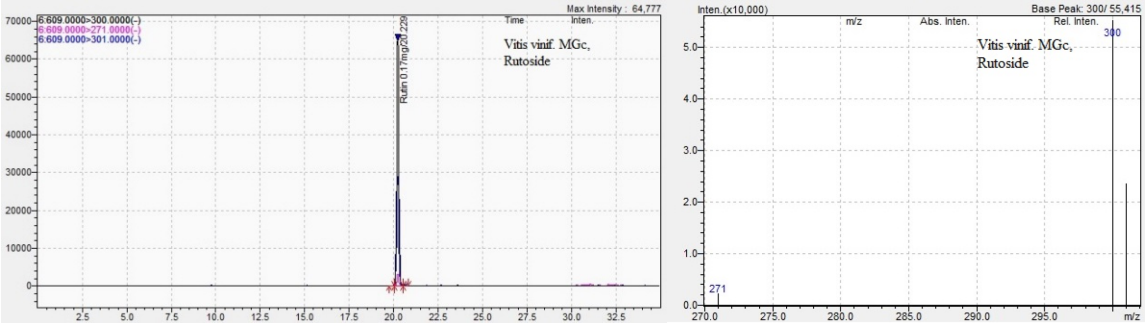

Figure S69. Common grape (Vvi) GTE chromatogram and associated MS spectrum of rutoside

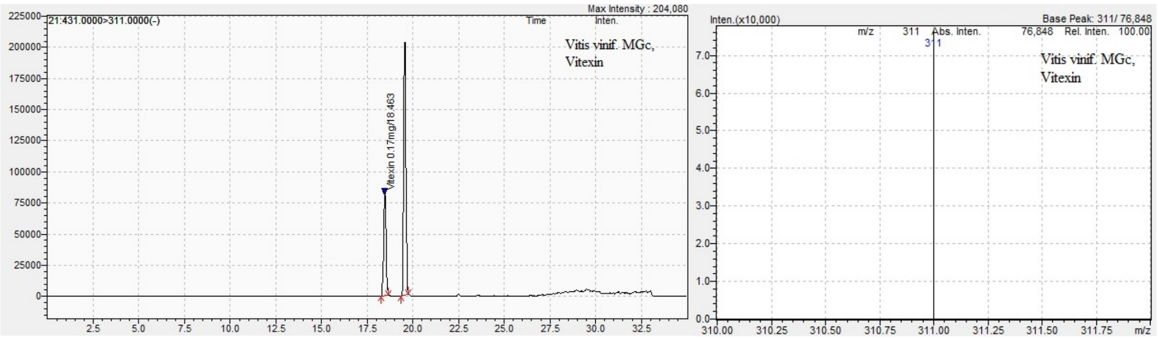

Figure S70. Common grape (Vvi) GTE chromatogram and associated MS spectrum of vitexin

Blackthorn (Psp) GTE:

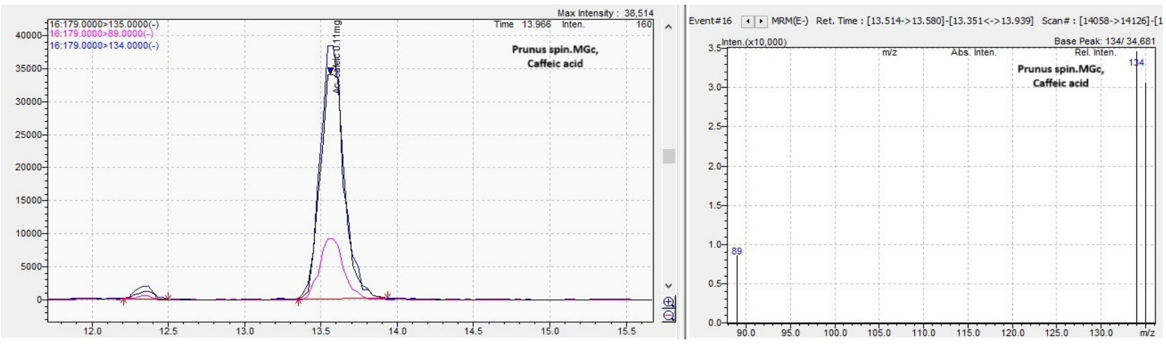

Figure S71. Blackthorn (Psp) GTE chromatogram and associated MS spectrum of caffeic acid

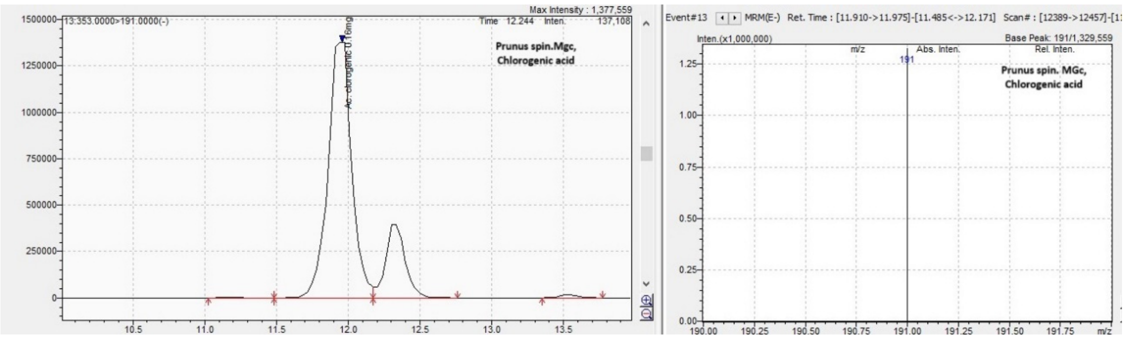

Figure S72. Blackthorn (Psp) GTE chromatogram and associated MS spectrum of chlorogenic acid

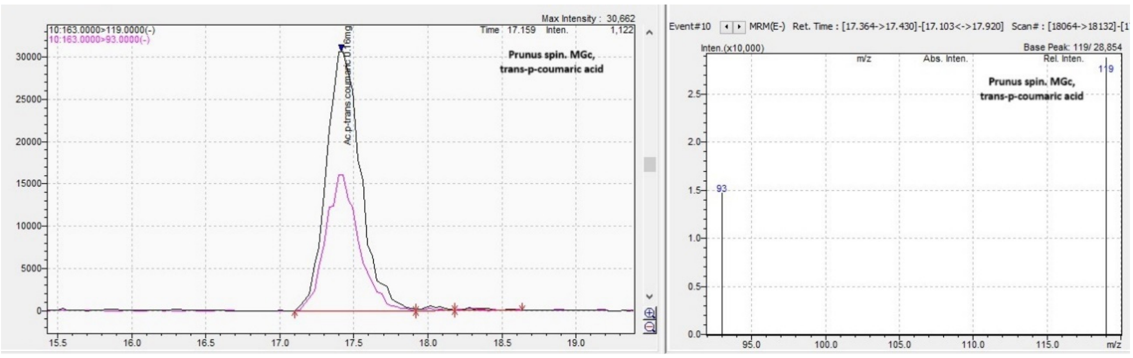

Figure S73. Blackthorn (Psp) GTE chromatogram and associated MS spectrum of trans-p-coumaric acid

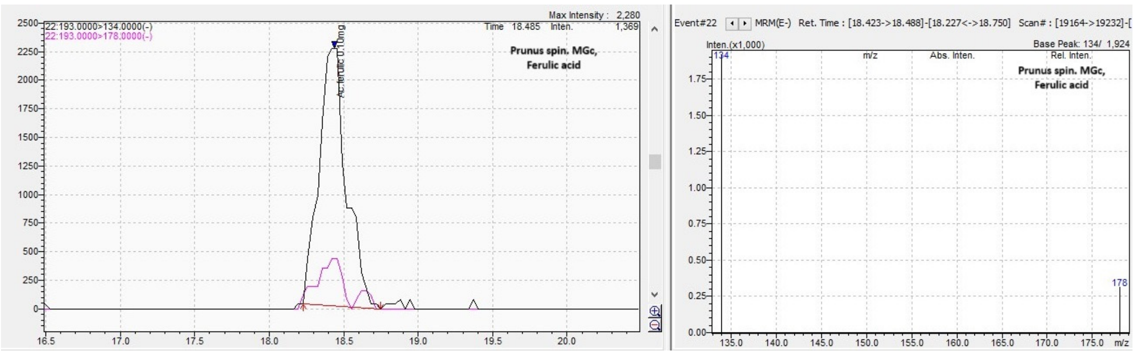

Figure S74. Blackthorn (Psp) GTE chromatogram and associated MS spectrum of ferulic acid

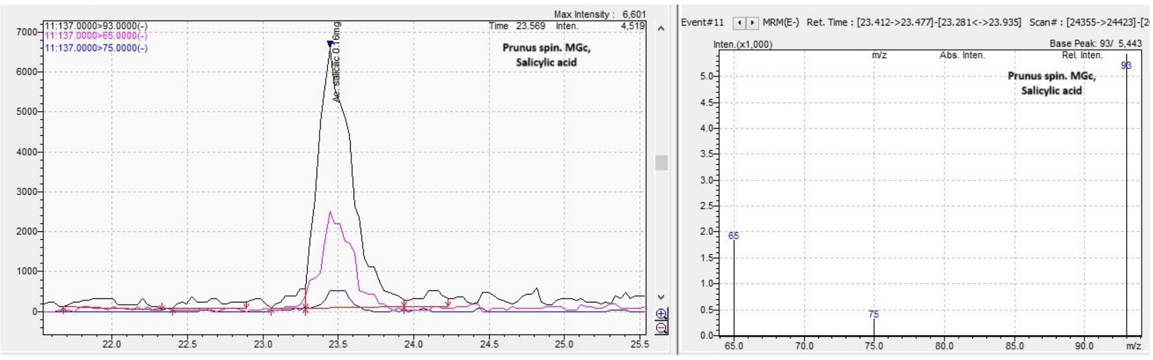

Figure S75. Blackthorn (Psp) GTE chromatogram and associated MS spectrum of salycilic acid

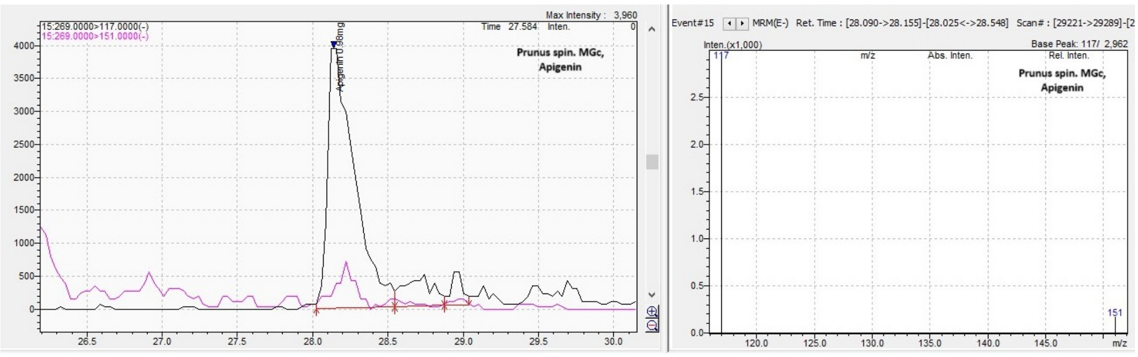

Figure S76. Blackthorn (Psp) GTE chromatogram and associated MS spectrum of apigenin

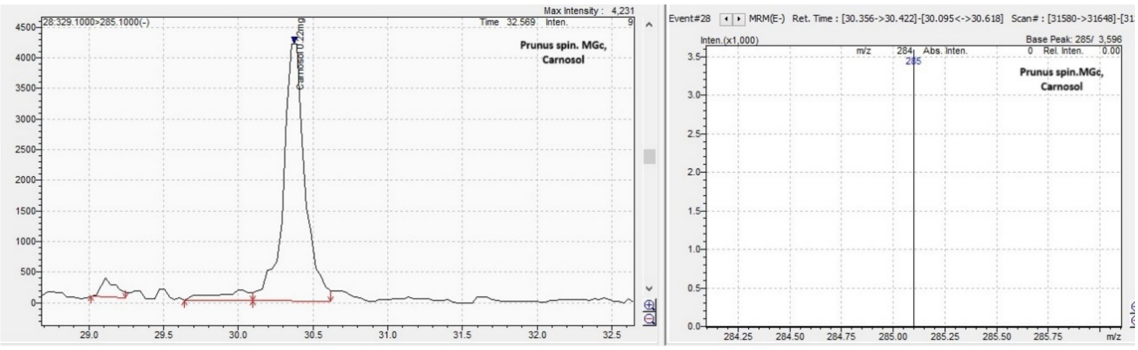

Figure S77. Blackthorn (Psp) GTE chromatogram and associated MS spectrum of carnosol

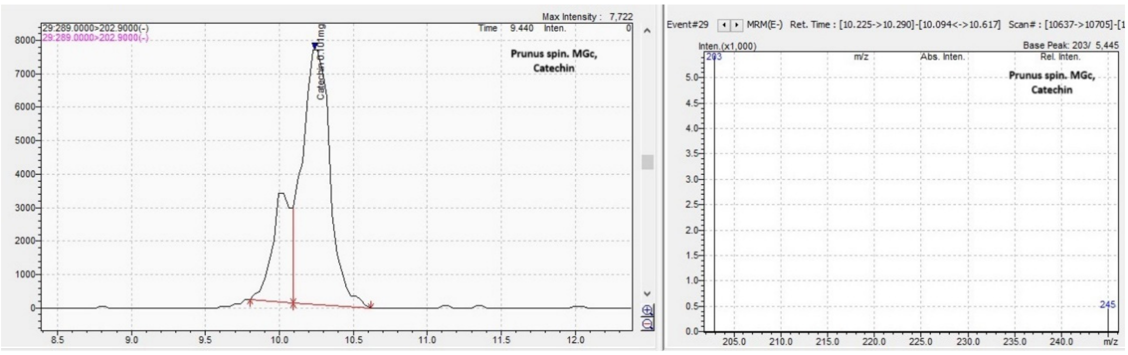

Figure S78. Blackthorn (Psp) GTE chromatogram and associated MS spectrum of catechin

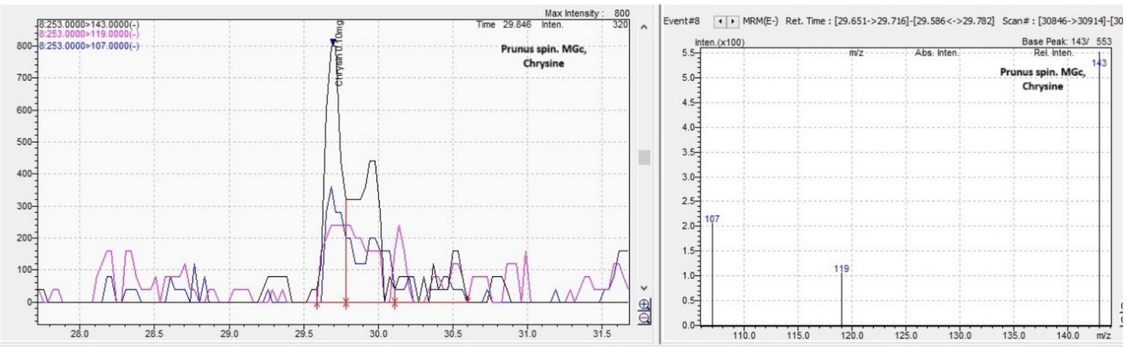

Figure S79. Blackthorn (Psp) GTE chromatogram and associated MS spectrum of chrysin

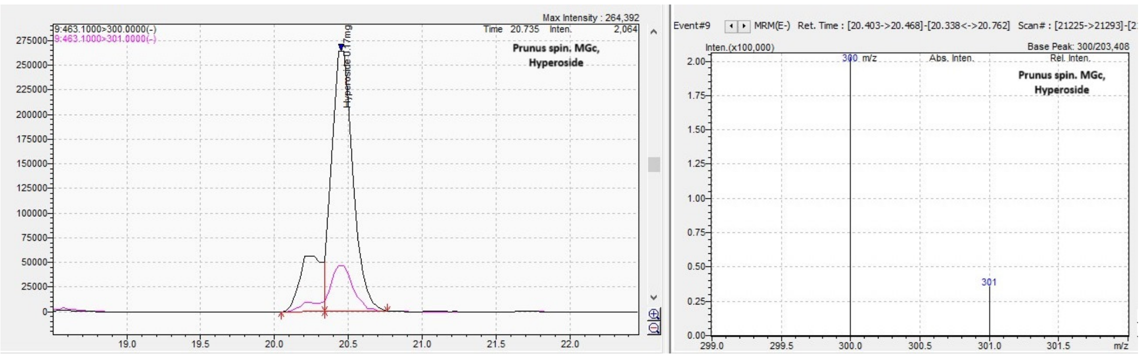

Figure S80. Blackthorn (Psp) GTE chromatogram and associated MS spectrum of hyperoside

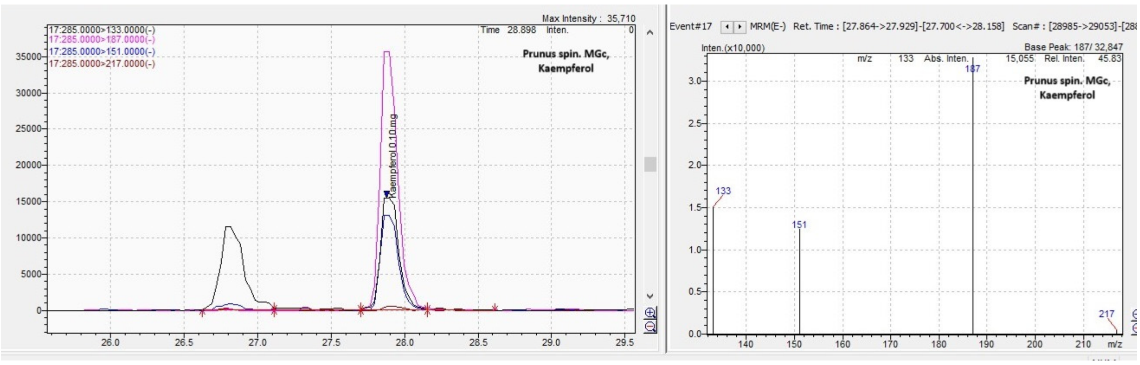

Figure S81. Blackthorn (Psp) GTE chromatogram and associated MS spectrum of kaempferol

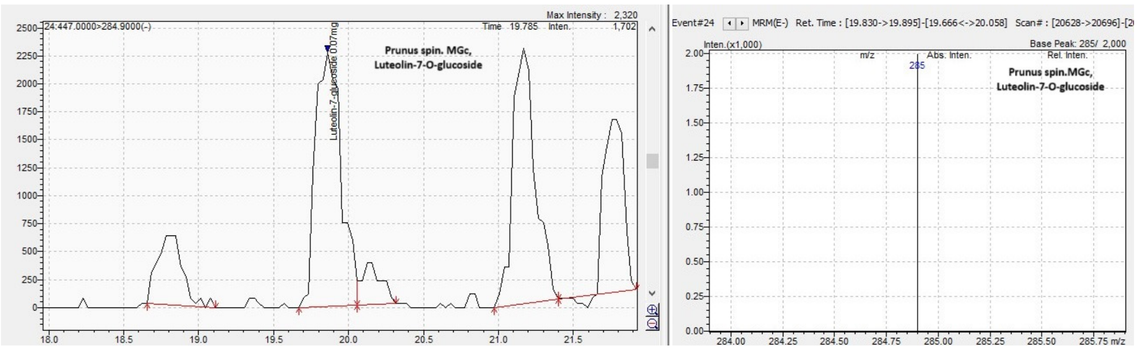

Figure S82. Blackthorn (Psp) GTE chromatogram and associated MS spectrum of luteolin-7-O-glucoside

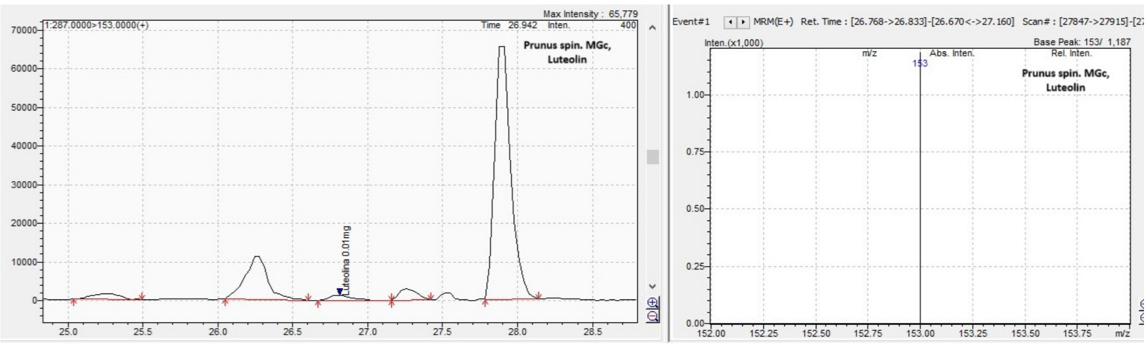

Figure S83. Blackthorn (Psp) GTE chromatogram and associated MS spectrum of luteolin

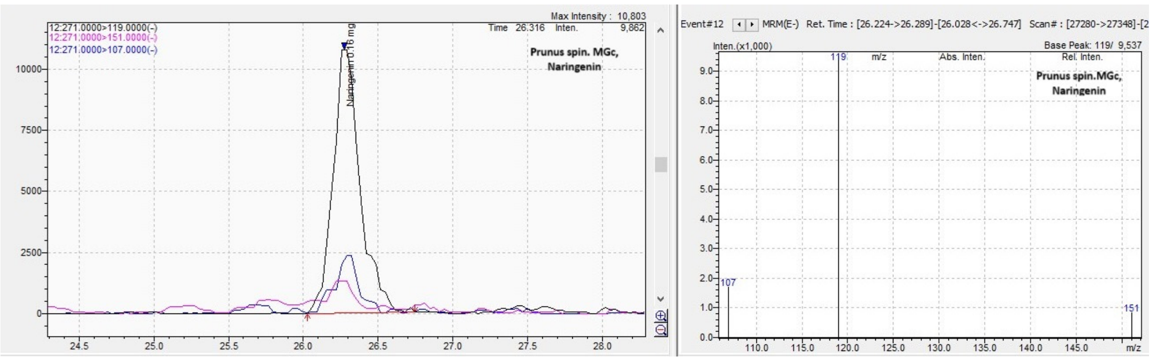

Figure S84. Blackthorn (Psp) GTE chromatogram and associated MS spectrum of naringenin

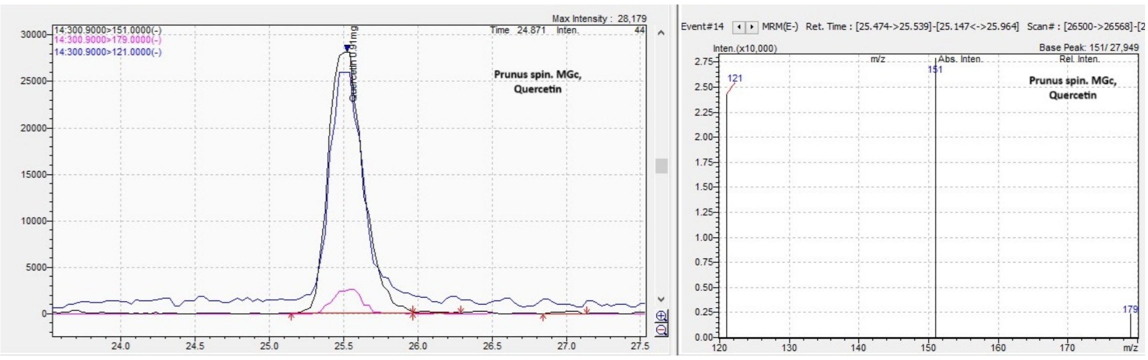

Figure S85. Blackthorn (Psp) GTE chromatogram and associated MS spectrum of quercetin

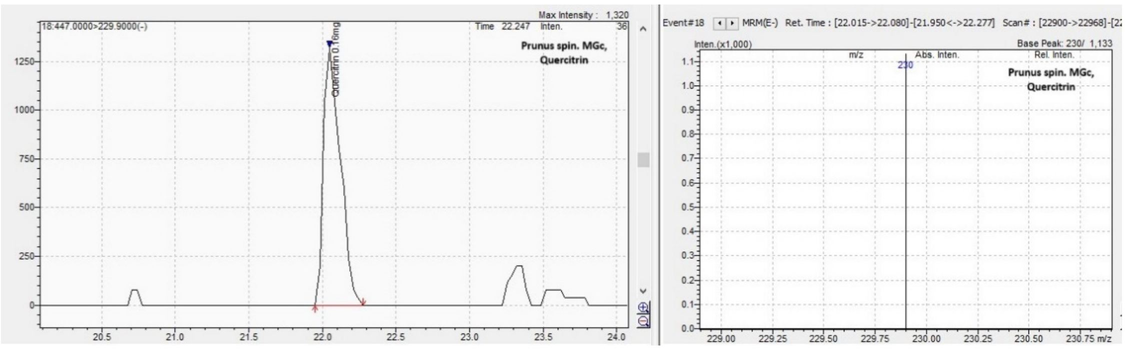

Figure S86. Blackthorn (Psp) GTE chromatogram and associated MS spectrum of quercitrin

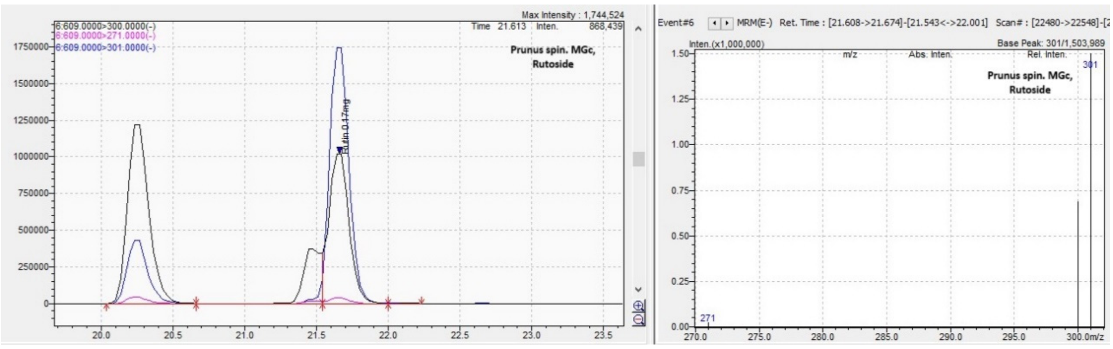

Figure S87. Blackthorn (Psp) GTE chromatogram and associated MS spectrum of rutoside

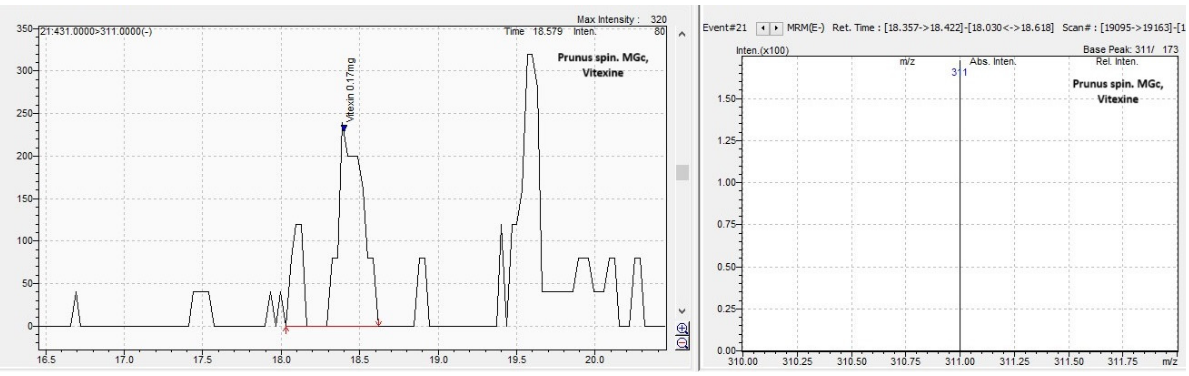

Figure S88. Blackthorn (Psp) GTE chromatogram and associated MS spectrum of vitexin

Sea buckthorn (Hrh) GTE:

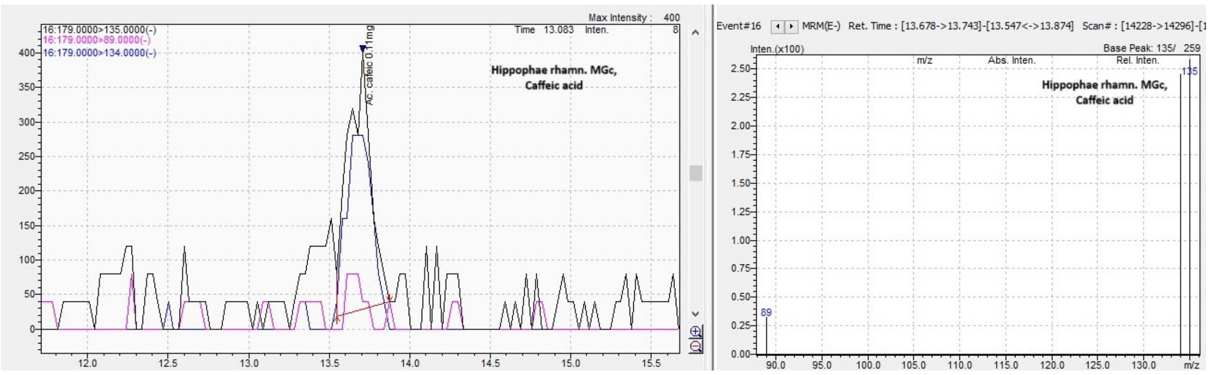

Figure S89. Sea buckthorn (Hrh) GTE chromatogram and associated MS spectrum of caffeic acid

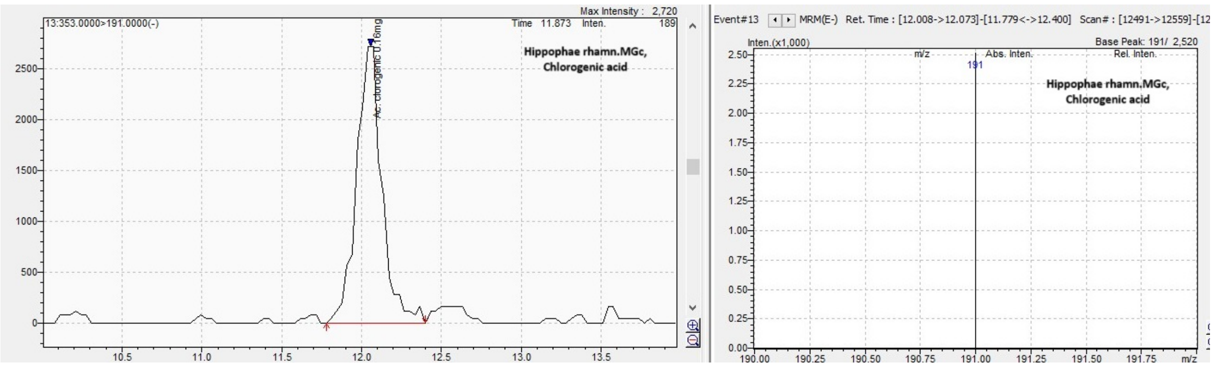

Figure S90. Sea buckthorn (Hrh) GTE chromatogram and associated MS spectrum of chlorogenic acid

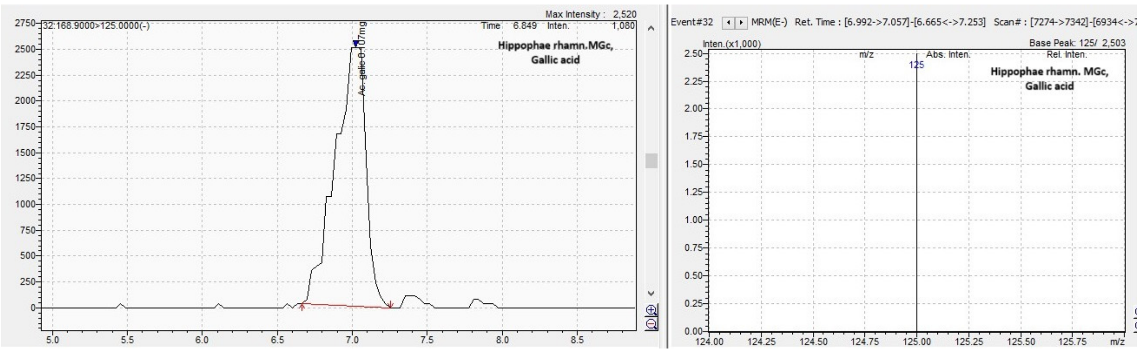

Figure S91. Sea buckthorn (Hrh) GTE chromatogram and associated MS spectrum of gallic acid

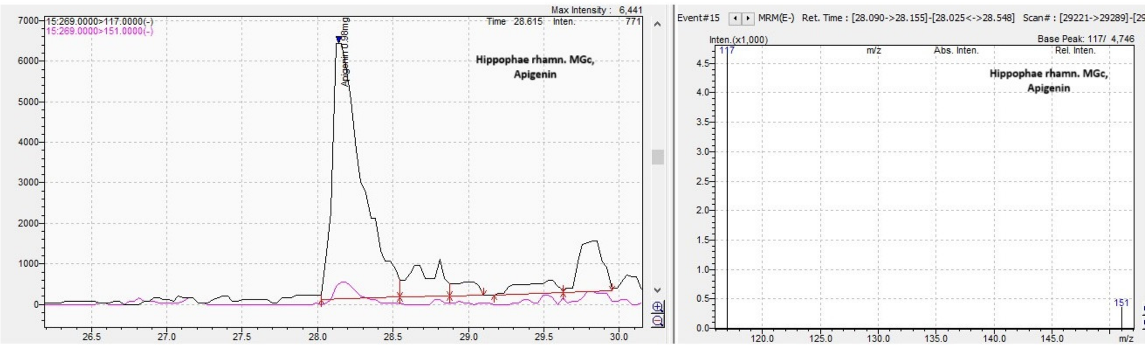

Figure S92. Sea buckthorn (Hrh) GTE chromatogram and associated MS spectrum of apigenin

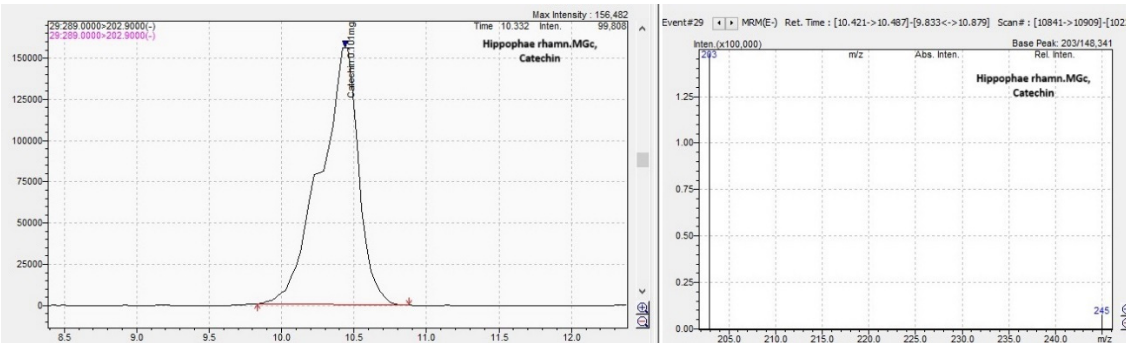

Figure S93. Sea buckthorn (Hrh) GTE chromatogram and associated MS spectrum of catechin

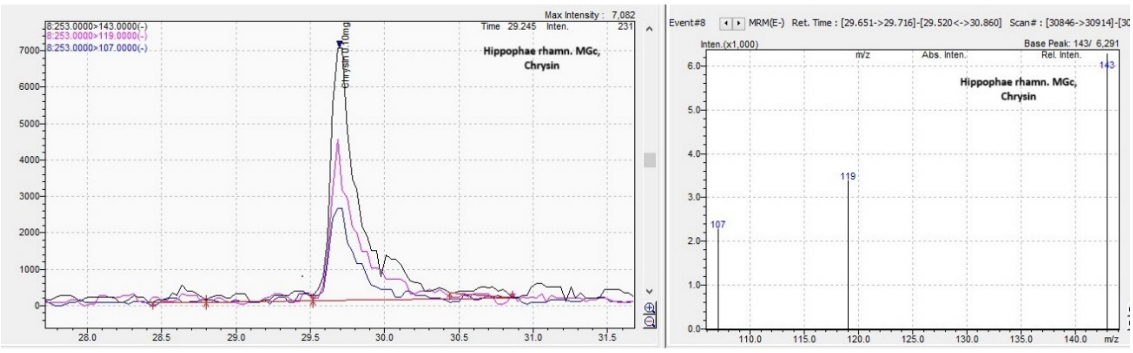

Figure S94. Sea buckthorn (Hrh) GTE chromatogram and associated MS spectrum of chrysin

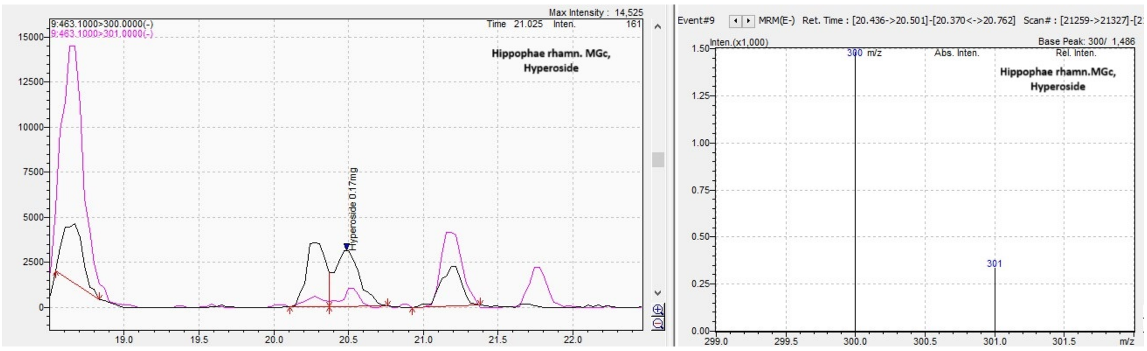

Figure S95. Sea buckthorn (Hrh) GTE chromatogram and associated MS spectrum of hyperoside

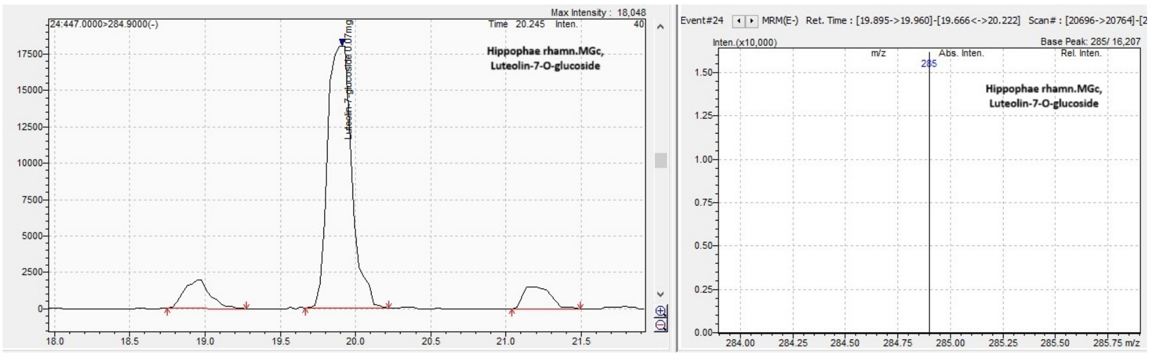

Figure S96. Sea buckthorn (Hrh) GTE chromatogram and associated MS spectrum of luteolin-7-O-glucoside

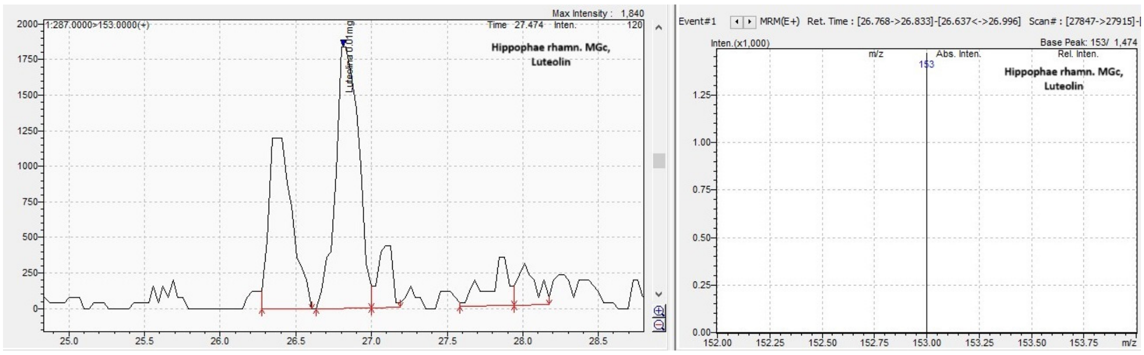

Figure S97. Sea buckthorn (Hrh) GTE chromatogram and associated MS spectrum of luteolin

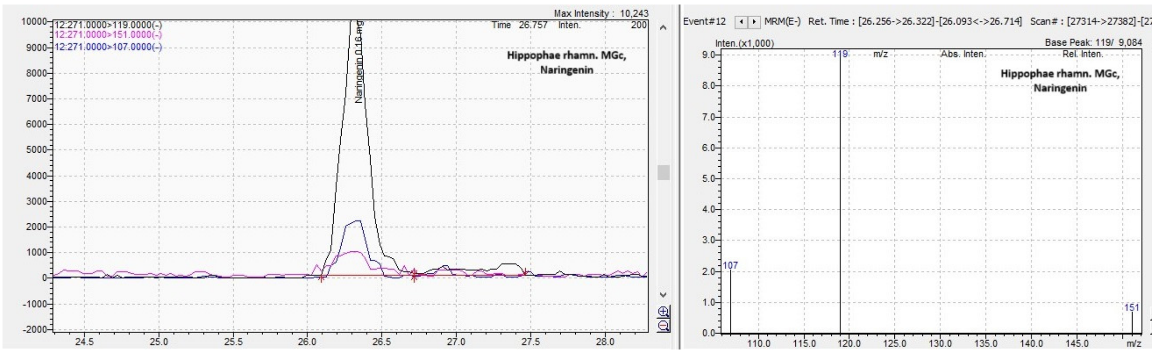

Figure S98. Sea buckthorn (Hrh) GTE chromatogram and associated MS spectrum of naringenin

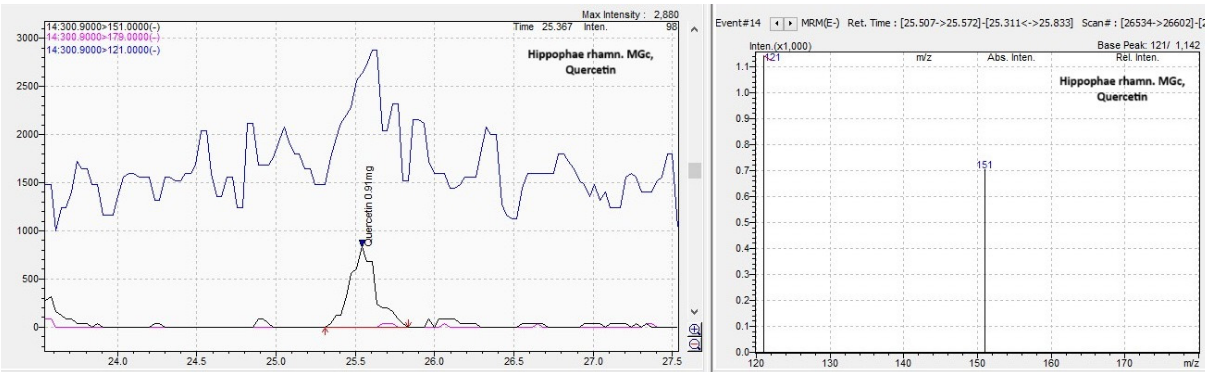

Figure S99. Sea buckthorn (Hrh) GTE chromatogram and associated MS spectrum of quercetin

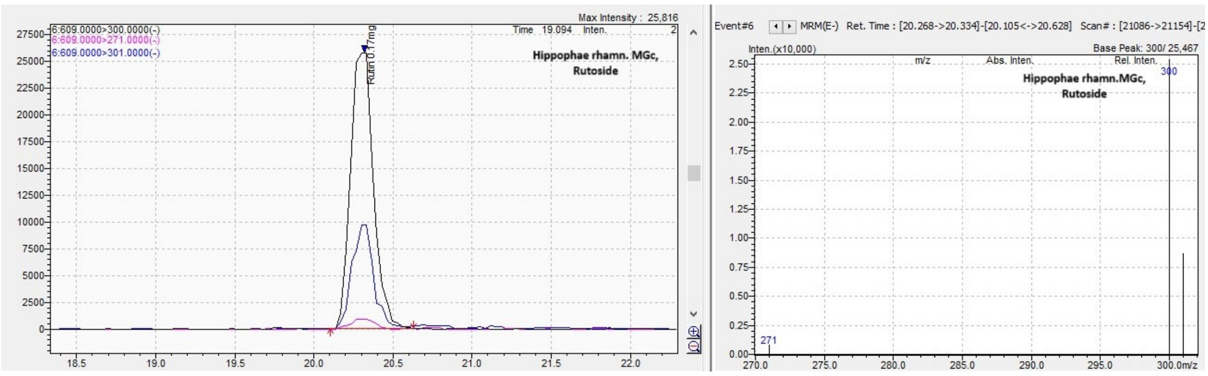

Figure S100. Sea buckthorn (Hrh) GTE chromatogram and associated MS spectrum of rutoside
